# Alkyltriphenylphosphonium Binding to Cardiolipin Triggers Oncosis in Cancer Cells

**DOI:** 10.1002/advs.202510237

**Published:** 2026-01-25

**Authors:** Jin Li, Hang Zheng, Yuxing Lin, Ziwei Zhao, Yijia Mai, Zhangrong Lou, Qiang Liu, Zhao Ma, Chengjun Wu

**Affiliations:** ^1^ Faculty of Medicine Dalian University of Technology Dalian Liaoning China; ^2^ Department of Medicinal Chemistry Key Laboratory of Chemical Biology (MOE) Shandong Key Laboratory of Druggability Optimization and Evaluation for Lead Compounds School of Pharmaceutical Sciences Cheeloo College of Medicine Shandong University Jinan Shandong China; ^3^ Institute of Medical Sciences The Second Hospital Cheeloo College of Medicine Shandong University Jinan Shandong China; ^4^ School of Life and Health Sciences Qingdao Central Hospital University of Health and Rehabilitation Sciences Qingdao Shandong China

**Keywords:** cancer cells, cardiolipin, mitochondria, oncosis, triphenylphosphonium

## Abstract

Mitochondria, pivotal for cellular bioenergetics and signaling, are attractive targets for cancer therapy. Triphenylphosphonium (TPP^+^) is a widely used mitochondrial‐targeting ligand, yet its intrinsic bioactivity and mechanism remain underexplored. Here we demonstrate that alkylated TPP^+^ derivatives exhibit chain length‐dependent anticancer activity, with TPP^+^‐C_14_ showing superior efficacy both in vitro and in vivo. Mechanistically, TPP^+^‐C_14_ selectively binds to cardiolipin, a key phospholipid in the inner mitochondrial membrane, through electrostatic and hydrophobic interactions, as validated by biolayer interferometry, competitive binding assays, and molecular dynamics simulations. This binding impairs cardiolipin function, leading to mitochondrial membrane potential collapse, adenosine triphosphate depletion, metabolic reprogramming, and ultimately mitochondrial dysfunction. Intriguingly, TPP^+^‐C_14_ induces oncosis in cancer cells, rather than apoptosis or autophagy, by activating the endoplasmic reticulum stress pathway. These findings reveal a novel bioactive mechanism for TPP^+^ beyond its intrinsic mitochondrial targeting property, providing a foundation for next‐generation mitochondrial‐targeted anticancer strategies that could precisely modulate mitochondrial functions.

## Graphical abstract

Alkyltriphenylphosphonium, exemplified by TPP^+^‐C_14_, preferentially accumulates in mitochondria and selectively binds to cardiolipin, a key phospholipid of the inner mitochondrial membrane, causing loss of mitochondrial membrane potential, severe cellular ATP depletion, and calcium imbalance. These disruptions lead to mitochondrial dysfunction and trigger endoplasmic reticulum stress, ultimately inducing oncosis in cancer cells.

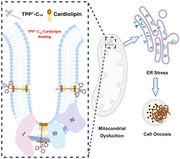

## 1 Introduction

Mitochondria, essential organelles in most eukaryotic cells, are structured into four functional regions: the outer mitochondrial membrane (OMM), intermembrane space, inner mitochondrial membrane (IMM), and matrix [1]. These specialized compartments coordinate critical cellular processes, including bioenergetics, biosynthesis, redox homeostasis, and programmed cell death, such as apoptosis, autophagic cell death, and oncosis [2, 3, 4]. In tumors, malignant cells orchestrate a profound mitochondrial reprogramming to support their heightened demands for energy and metabolic precursors, enabling mitochondria to be plausible, as yet underexploited targets for cancer treatment [5, 6]. The hallmark of mitochondrial function is adenosine triphosphate (ATP) production through oxidative phosphorylation (OXPHOS), which fuels cell survival, proliferation, and other vital processes [7]. This process depends on a robust mitochondrial membrane potential (MMP) across the IMM, typically ranging from −140 to −180 mV. The MMP drives the selective accumulation of lipophilic cations, such as triphenylphosphonium (TPP^+^), in the mitochondrial matrix at concentrations 100 to 500 times higher than in the cytosol [8]. This mitochondriotropicism enables TPP^+^‐based therapeutics, such as Mito‐Metformin and Mito‐Doxorubicin, to precisely target mitochondria, thereby enhancing drug efficacy [8, 9, 10]. These conjugates, typically composed of three key elements: TPP^+^ moiety, linker, and therapeutic payload, are thought to exert significantly enhanced therapeutic effects by altering the subcellular localization of molecules [11, 12]. While traditionally considered biologically inert, emerging evidence suggests that TPP^+^ could directly modulate mitochondrial functions beyond its mitochondriotropic property [13, 14, 15, 16]. Understanding the biological effects and mechanisms of TPP^+^ and its derivatives is imperative for refining mitochondrial targeting therapeutic modalities.

A key stabilizer of mitochondrial structure and function is cardiolipin, a dimeric phospholipid constituting ∼20% of the IMM's phospholipid content [17, 18]. Structurally, cardiolipin is a diphosphatidylglycerol with two phosphatidyl residues linked by a glycerol bridge and four acyl chains [19]. Its pronounced negative charge at physiological pH and inverted conical geometry promote negative membrane curvature, facilitating cristae folding and dynamic remodeling critical for mitochondrial architecture and function [20, 21]. Predominantly located in the matrix‐facing leaflet of the IMM, cardiolipin anchors electron transport chain (ETC) complexes and ATP synthase through electrostatic and hydrophobic interactions, ensuring efficient OXPHOS [22, 23, 24]. Disruption of cardiolipin homeostasis impairs MMP, destabilizes mitochondrial structure, and compromises energy production, typically triggering cell death pathways like apoptosis or autophagy [25, 26, 27, 28]. These characteristics establish cardiolipin as a compelling therapeutic target for modulating mitochondrial function and cell death pathways in diseases.

**FIGURE 1:**
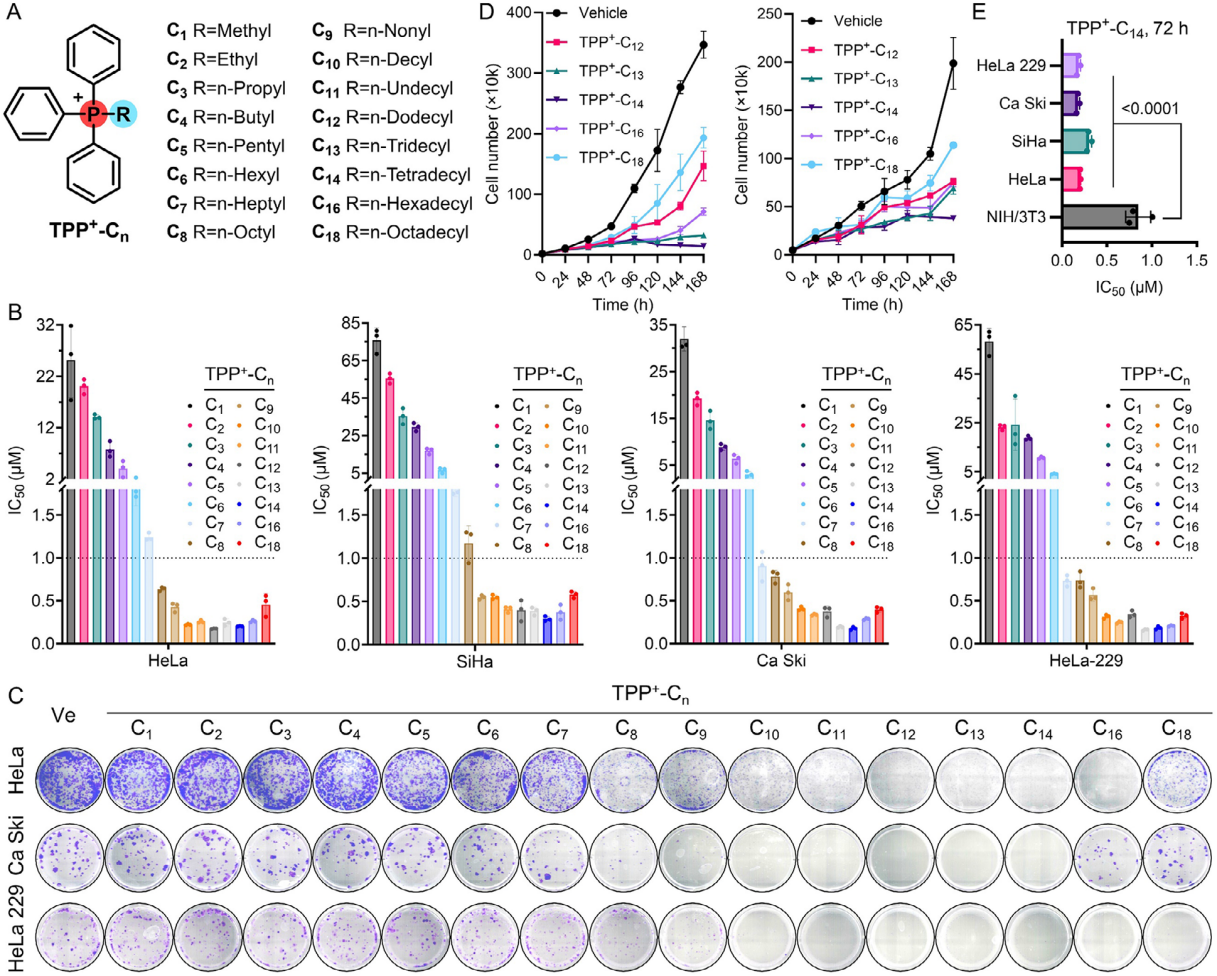
Antiproliferative effects of alkylated TPP^+^ derivatives on cervical cancer cells. (A) Chemical structures of TPP^+^‐C_n_. (B) IC_50_ values of TPP^+^‐C_n_ against various cervical cancer cell lines after a 72‐h treatment. (C) Clonogenic assay of the indicated cells that were treated with 100 nm TPP^+^‐C_n_ for about 14 days, respectively. (D) Proliferation curves of HeLa (left) and SiHa (right) cells treated with the corresponding compound (150 nm). (E) Cytotoxicity of TPP^+^‐C_14_ on NIH/3T3 murine embryonic fibroblasts versus cervical cancer cell lines. Data are shown as mean ± SD (n = 3). Statistical significance was determined using one‐way ANOVA by Dunnett's multiple comparisons test.

Given the colocalization of positively charged TPP^+^ in the mitochondrial matrix and negatively charged cardiolipin in the IMM, their electrostatic complementarity suggests potential interactions that could influence mitochondrial function and cell fate. To investigate this, we synthesized a series of alkyltriphenylphosphonium (alkylTPP^+^) derivatives, TPP^+^‐C_n_, with alkyl chains ranging from C_1_ to C_18_ (Figure 1A). Our studies revealed a strong correlation between the anticancer activity of these compounds and the length of their alkyl chains, with potency increasing as the chain lengthened. Importantly, the binding affinity to cardiolipin followed the same trend, indicating that their potency is dependent on cardiolipin binding. The representative compound TPP^+^‐C_14_, with exceptional affinity for cardiolipin, demonstrated potent tumor growth suppression in both in vitro and in vivo studies, highlighting its potential as an anticancer agent. Unlike typical cardiolipin‐mediated mitochondrial dysfunction, which induces apoptosis or autophagy, TPP^+^‐C_14_ triggered a distinct cell death pathway characterized by cellular vacuolization, mitochondrial dilation, and profound ATP depletion. These features, along with elevated reactive oxygen species (ROS), disrupted Ca^2+^ homeostasis, and metabolic reprogramming, align with oncosis, a programmed cell death mechanism driven by compromised cell membrane integrity, ATP depletion, mitochondrial dysfunction, and disrupted ion homeostasis [29]. Upregulation of the oncosis marker Porimin confirmed TPP^+^‐C_14_’s induction of oncotic cell death. Mechanistically, TPP^+^‐C_14_’s triggers oncosis by activation of the PERK/eIF2α/ATF4/CHOP endoplasmic reticulum (ER) stress pathway. Our work elucidates a novel cardiolipin‐targeted mechanism for alkylated TPP^+^ derivatives, offering new insights into anticancer drug development leveraging mitochondrial targeting ligands.

## 2 Results and Discussion

### 2.1 Alkylated TPP^+^ Derivatives Exhibit Alkyl Chain Length‐Dependent Antiproliferative Activities

Previous studies have established that the TPP^+^ moiety elicits potent antiproliferative activities when conjugated with a long alkyl chain, such as decyl (C_10_) or dodecyl (C_12_) [16]. However, the structure‐activity relationship (SAR) of this kind of compound remains inadequately characterized, limiting the rational structure optimization and mechanism analysis for therapeutic applications. To address this gap, we synthesized a comprehensive series of alkylated TPP^+^ derivatives with alkyl chain lengths ranging from C_1_ to C_18_ (Figure 1A; Scheme S1), whose chemical structures were well‐characterized using spectroscopic methods (Figures S22–S69). These compounds were subjected to the quantitative 72‐h cell viability assays in four cervical cancer cell lines (HeLa, SiHa, Ca Ski, and HeLa 229) to assess their cytotoxicity. The results revealed a clear trend of chain‐length‐dependent cytotoxicity, with antiproliferative potency increasing progressively with longer alkyl chains (Figure 1B). A critical inflection point was observed at the C_9_ derivative (TPP^+^‐C_9_), which achieved submicromolar IC_50_ values (∼0.5 µm) across all tested cell lines (Table S1). Further elongation of the alkyl chain beyond C_9_ resulted in only marginal improvements in potency, indicating a saturation of antiproliferative efficacy at this chain length. This trend was consistent across all four cell lines, indicating robust reproducibility of the SAR profile.

To further validate these findings, we then performed the clonogenic survival assays and cell growth inhibition studies. These experiments not only corroborated the observed chain‐length‐dependent antiproliferative trend of alkylated TPP^+^ compounds but also provided additional insights into the subtle differences among derivatives with chain lengths exceeding C_9_ (Figure 1C,D). Among the series, TPP^+^‐C_14_ emerged as the most efficacious compound across all assays, demonstrating superior antiproliferative activity. Moreover, the alkyl chain itself, as represented by n‐tetradecane (C_14_) and 1‐tetradecanol (C_14_‐OH), exhibits no detectable effect on tumor cell proliferation (Figure S1). To evaluate the therapeutic selectivity of TPP^+^‐C_14_, parallel cytotoxicity evaluations were also performed on NIH/3T3 mouse fibroblast cells, a non‐malignant cell model. The cells exhibited significantly higher tolerance to TPP^+^‐C_14_ exposure, with an IC_50_ value of ∼820 nm, compared to the IC_50_ values below 300 nm observed in the malignant cervical cancer cell lines (Figure 1E). This differential cytotoxicity suggests that TPP^+^‐C_14_ possesses a favorable therapeutic index, preferentially targeting cancer cells while sparing non‐cancerous cells. This selectivity is a critical attribute for minimizing off‐target toxicity in potential therapeutic applications.

### 2.2 TPP^+^‐C_14_ Potently Suppresses the Growth of Cervical Cancer Xenografts in Mice

**FIGURE 2:**
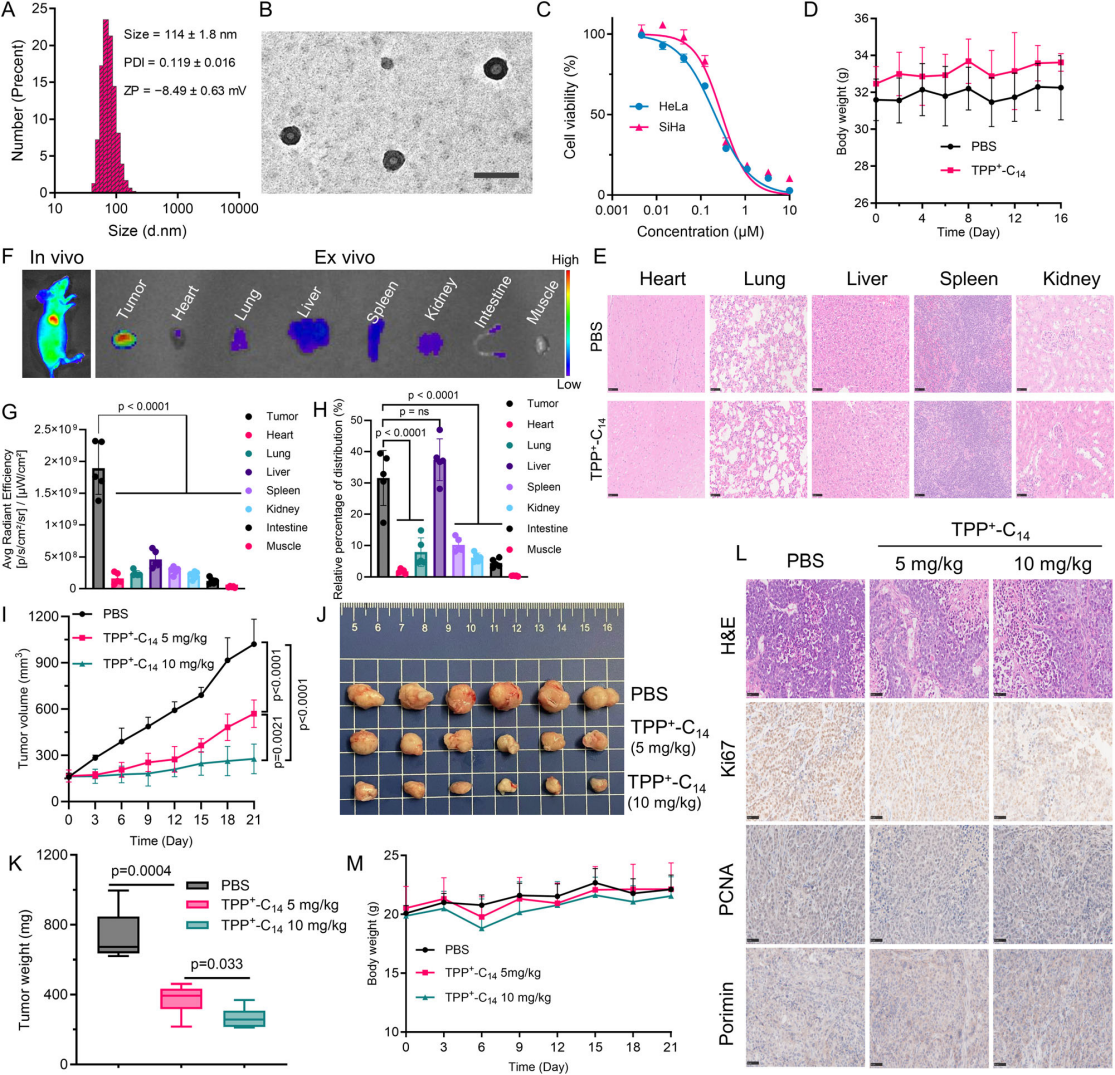
Characterization of liposomal TPP^+^‐C_14_ and its toxicity and antitumor activity in mice. (A) Size distribution and zetapotential of liposomal TPP^+^‐C_14_. (B) Representative image of liposomal TPP^+^‐C_14_ using transmission electron microscopy (TEM). Scale bar: 200 nm. (C) Dose‐dependent antiproliferation of liposomal TPP^+^‐C_14_ (72 h). (D,E) In vivo toxicity assessment of liposomal TPP^+^‐C_14_ in ICR mice (n = 6/group). Mice were injected by tail vein with TPP^+^‐C_14_ (10 mg/kg) every 3 days. D) Body weight changes (n = 6); E) Histopathological analysis of major organs, Scale bar: 50 µm. (F) In vivo and ex vivo biodistribution imaging of liposomal TPP^+^‐C_14_ in HeLa‐derived xenograft mouse model. Mice were injected intravenously with DiD‐labeled liposomal TPP^+^‐C_14_ (5 mg/kg). 24 h post‐injection, biodistribution was assessed through in vivo and ex vivo imaging using an excitation/emission wavelength of 640/700 nm. (G) Quantitative analysis of fluorescence signals in major organs and tumor tissue (n = 5). (H) Relative percentage of fluorescence intensity across major organs and tumor tissue (n = 5). (I–M) Antitumor efficacy of liposomal TPP^+^‐C_14_ in the HeLa‐derived xenograft models (n = 6 per group). I) Tumor growth curve; J) Image of tumors harvested at the endpoint; K) Tumor weight; L) Hematoxylin and eosin (H&E) and immunohistochemical (IHC) analysis of tumor section, Scale bar: 50 µm; M) Body weight curves during treatment. All data in the corresponding panel are shown as mean ± SD. Statistical significance was determined using a two‐tailed Student's t‐test for K, while using one‐way ANOVA by Dunnett's multiple comparisons test for G, H, and I.

Building on the promising in vitro antiproliferative activity, we wondered whether TPP^+^‐C_14_ has therapeutic effects against mouse xenografts of cervical cancer. To address the water solubility issue, we prepared a liposomal formulation for TPP^+^‐C_14_ (Figure 2A,B) [30, 31]. The surface charge of the liposomal TPP^+^‐C_14_ is approximately −8.5 mV (Figure 2A), notably less negative than that of blank liposomes (∼−25 mV) (Figure S2A). This shift suggests effective electrostatic interactions between the positively charged TPP^+^‐C_14_ and the negatively charged liposome membrane. Furthermore, this formulation demonstrates a melting enthalpy comparable to that of blank liposomes, indicative of similar crystallinity, as confirmed by differential scanning calorimetry analysis (Figure S2B,C), while preserving bioactivity equivalent to free TPP^+^‐C_14_ (Figure 2C). Repeated intravenous injections of liposomal TPP^+^‐C_14_ (10 mg/kg, every 3 days, 5 total doses) into ICR mice elicited no significant body weight loss (Figure 2D). Hematological parameters, comprehensive metabolic panel, and histopathological analysis of major organs revealed no abnormalities (Figure 2E; Figure S3), confirming the systemic tolerability of TPP^+^‐C_14_ by mice at the tested dosage.

To assess the therapeutic potential of TPP^+^‐C_14_ against tumors in vivo, the HeLa cell‐derived xenografts were established in nude mice. Biodistribution studies utilizing DiD‐labeled liposomal TPP^+^‐C_14_ (Figure S2D) in these models demonstrated selective accumulation at the tumor site through both in vivo and ex vivo fluorescence imaging (Figure 2F), confirming effective tumor targeting. Quantitative fluorescence analysis and relative percentage distribution across major organs (Figure 2G,H) further validated preferential tumor accumulation, providing robust evidence of TPP^+^‐C_14_’s tumor‐specific delivery and therapeutic potential. For the treatment study, mice were randomized into three groups (n = 6 per group): PBS control, low‐dose TPP^+^‐C_14_ (5 mg/kg), and high‐dose TPP^+^‐C_14_ (10 mg/kg) when the average tumor volume reached 100 mm^3^. TPP^+^‐C_14_ demonstrated dose‐dependent antitumor activity in mice, with high‐dose treatment achieving a 73% reduction in tumor volume and a 64% decrease in tumor mass versus controls at the endpoint (Figure 2I−K). Hematoxylin and eosin (H&E) staining and immunohistochemical (IHC) patterns revealed the decreased tumor cell density and a remarkable downregulation of the proliferation markers Ki67 and PCNA in TPP^+^‐C_14_ ‐treatment groups (Figure 2L; Figure S4), confirming the suppression of tumor growth. This therapeutic efficacy was accompanied by sustained murine health, as evidenced by stable body weight profiles during treatment (Figure 2M).

### 2.3 TPP^+^‐C_14_ Accumulates in Mitochondria and Binds to the Cardiolipin

**FIGURE 3:**
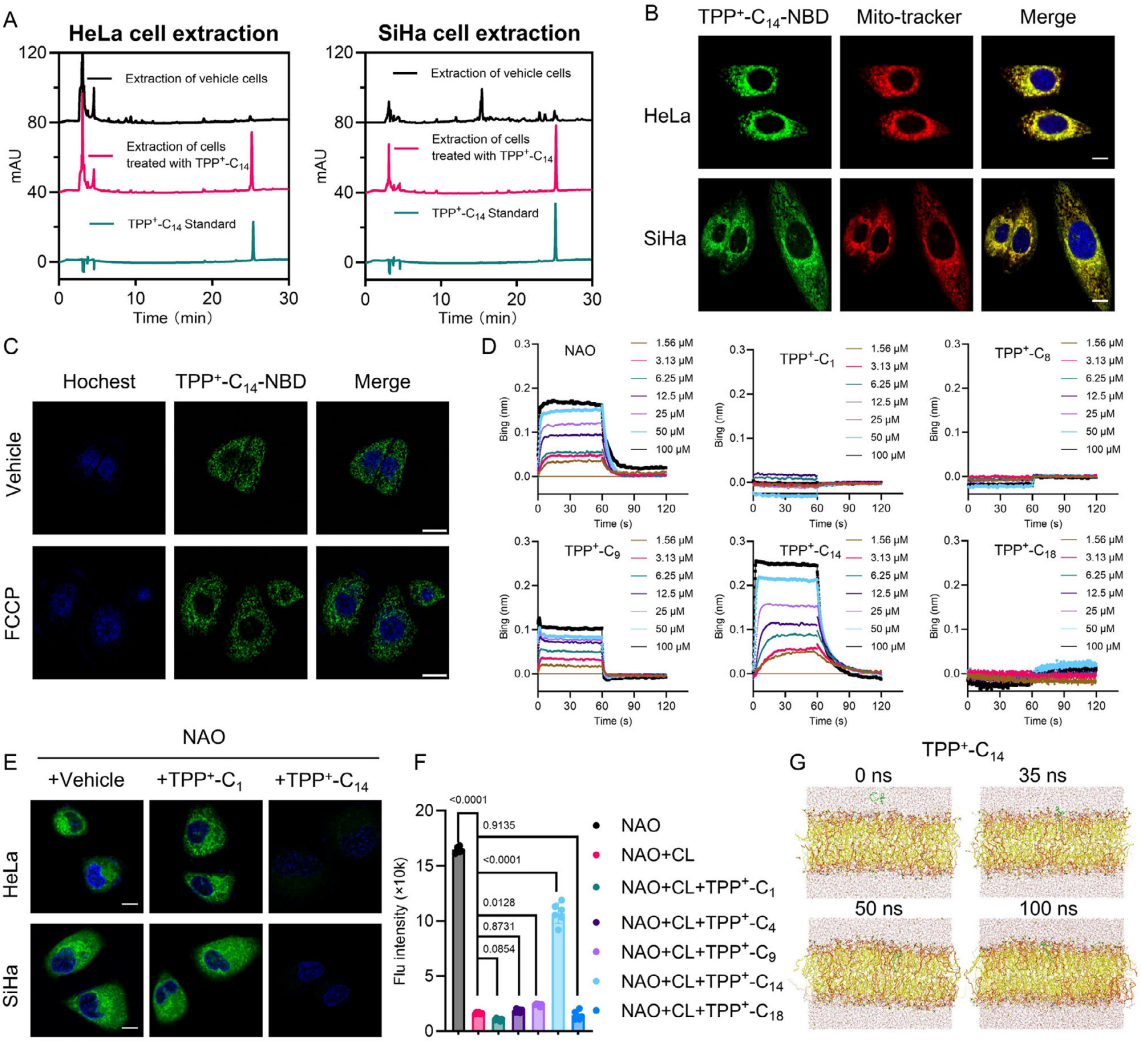
Mitochondrial targeting and cardiolipin‐binding properties of TPP^+^‐C_14_. (A) HPLC analysis of the mitochondrial accumulation of TPP^+^‐C_14_. Cells were treated with 20 µm TPP^+^‐C_14_ for 24 h, and their mitochondria were isolated, followed by the HPLC test. (B) Representative confocal microscopy images demonstrating colocalization of TPP^+^‐C_14_‐NBD (1 µm, 30 min) and MitoTracker Deep Red (200 nm, 30 min) in cells. Scale bar: 10 µm. (C) Mitochondrial membrane potential (MMP)‐independent mitochondrial accumulation of TPP^+^‐C_14_ ‐NBD. Cells were incubated with or without 10 µm FCCP for 2 h, followed by treatment with TPP^+^‐C_14_ ‐NBD (1 µm, 30 min). Scale bar: 10 µm. (D) Biolayer interferometry (BLI) binding kinetics of cardiolipin with NAO or representative TPP^+^‐C_n_ derivatives. (E) Competitive cell imaging of NAO showing binding of alkyTPP^+^ to cardiolipin. Cells were incubated with 500 nm NAO for 30 min and then exposed to 1 µm TPP^+^‐C_1_ or TPP^+^‐C_14_ for 30 min. Scale bar: 10 µm. (F) Competitive binding assay of NAO and alkyTPP^+^ in PBS showing binding to cardiolipin. Cardiolipin (CL, 20 µm) was incubated with NAO (20 µm) and then exposed to representative TPP^+^‐C_n_ (20 µm), followed by fluorescence intensity measurement (ex/em: 499/530 nm). Data were shown as mean ± SD (n = 6). Statistical significance was determined using one‐way ANOVA by Dunnett's multiple comparisons test. (G) Molecular dynamics (MD) simulations showing the interactions between TPP^+^‐C_14_ and cardiolipin. Phosphatidylcholine (DOPC, yellow), tetralinoleoyl cardiolipin (TLCL, ‐2e charge, brown), and TPP^+^‐C_14_ (Green).

The TPP^+^ cation is extensively employed as the mitochondrial‐targeting carrier, typically conjugated to the therapeutic payload through an alkyl linker to enhance the therapeutic efficacy [32, 33, 34]. Our experimental findings, as detailed above, have confirmed that the TPP^+^ portion, when coupled with an alkyl chain, exhibits intrinsic antitumor activity independent of a conjugated payload. This observation prompted further investigation into the molecular mechanisms underlying this biological effect. Through the high‐performance liquid chromatography (HPLC) analysis of mitochondrial extracts from HeLa and SiHa cells, treated with or without TPP^+^‐C_14_ (20 µm, 24 h), it was confirmed that this compound substantially accumulates within the mitochondria (Figure 3A). For further confirmation, we also synthesized a fluorescently labeled derivative, TPP^+^‐C_14_ ‐NBD, by conjugating TPP^+^‐C_14_ with nitrobenzoxadiazole (NBD) (Scheme S2, and Figures S70−S77). Co‐incubation of TPP^+^‐C_14_‐NBD and MitoTracker Deep Red in cells demonstrated excellent fluorescence overlapping with Pearson's R values of 0.98 in HeLa cells and 0.96 in SiHa cells, respectively, suggesting the mitochondrial targeting property of TPP^+^‐C_14_ (Figure 3B). In addition, TPP^+^‐C_14_ maintained mitochondrial retention under conditions of diminished MMP, a phenomenon that may be attributed to its specific electrostatic and hydrophobic interaction with the IMM as previously characterized (Figure 3C) [35, 36].

As described above, the IMM‐specific cardiolipin exhibits complementary electrostatic profiles with TPP^+^, theoretically supporting their direct binding effect. To quantitatively evaluate this interaction, we analyzed six representative alkylated TPP^+^ derivatives using biolayer interferometry (BLI), with the commercially available cardiolipin probe 10‐N‐nonyl acridine orange (NAO) serving as a reference control [37]. The results declared that these compounds have a distinct chain length‐dependent binding specificity for cardiolipin: TPP^+^‐C_1_, TPP^+^‐C_4_, TPP^+^‐C_8_, and TPP^+^‐C_18_ showed negligible binding responses, whereas TPP^+^‐C_9_ and TPP^+^‐C_14_ demonstrated robust concentration‐dependent binding properties (Figure 3D; Figure S5, and Table S2). This binding trend strongly correlates with their anti‐proliferative activity, suggesting that enhanced therapeutic efficacy is linked to tighter cardiolipin binding. It is worth noting that TPP^+^‐C_14_ exhibits superior binding kinetics compared to other derivatives and even NAO (Table S2), as reflected by its association constant (K_on_ = 7970 ± 91/Ms) and dissociation rate constant (K_off_ = 0.052 ± 0.003/s).

As a widely used cardiolipin‐specific sensor, NAO exhibits environment‐dependent fluorescence behaviors [38, 39, 40]. In aqueous solutions, NAO forms aggregates with cardiolipin, leading to significant fluorescence quenching. Conversely, in cells, where cardiolipin is embedded in the hydrophobic IMM, NAO binding enables the specific mitochondrial staining independent of MMP [40]. Leveraging this property, we employed NAO to competitively probe alkylTPP^+^‐cardiolipin interactions. In cells, TPP^+^‐C_14_ effectively displaced NAO from mitochondrial cardiolipin, reducing fluorescence labeling intensity regardless of treatment order (Figure 3E; Figure S6). In PBS buffer, the addition of TPP^+^‐C_14_ to preformed NAO‐cardiolipin complexes substantially restored the quenched fluorescence, while other tested alkylTPP^+^ derivatives and alkyl chain compounds (C_14_ and C_14_‐OH) showed minimal effects (Figure 3F; Figure S7). These competitive binding assays conclusively confirmed the exceptional binding affinity of TPP^+^‐C_14_ to mitochondrial cardiolipin. Furthermore, molecular dynamics (MD) simulations were performed using GROMACS 2024.4 to analyze the interactions between cardiolipin and TPP^+^‐C_1_ or TPP^+^‐C_14_ [40]. The results revealed that TPP^+^‐C_14_ directly inserts into the lipid bilayer and binds to cardiolipin, whereas TPP^+^‐C_1_ exhibits no detectable binding to cardiolipin (Figure 3G; Figure S8A,B). These simulations also demonstrated that the interaction energy governing the binding of TPP^+^‐C_14_ to cardiolipin is dominated by contributions from both the short‐range Lennard–Jones (LJ) potential and Coulombic interactions (Figure S8C). According to the interaction energy profiles, it could be observed that the binding of TPP^+^‐C_14_ with cardiolipin requires a significantly lower energetic barrier than that with dioleoyl phosphatidylcholine (DOPC), a lipid component of the mitochondrial membrane. This highlights the molecular basis for the selective targeting of cardiolipin by TPP^+^‐C_14_ via van der Waals forces and electrostatic attractions.

### 2.4 TPP^+^‐C_14_ Potently Disrupts Mitochondrial Function

**FIGURE 4:**
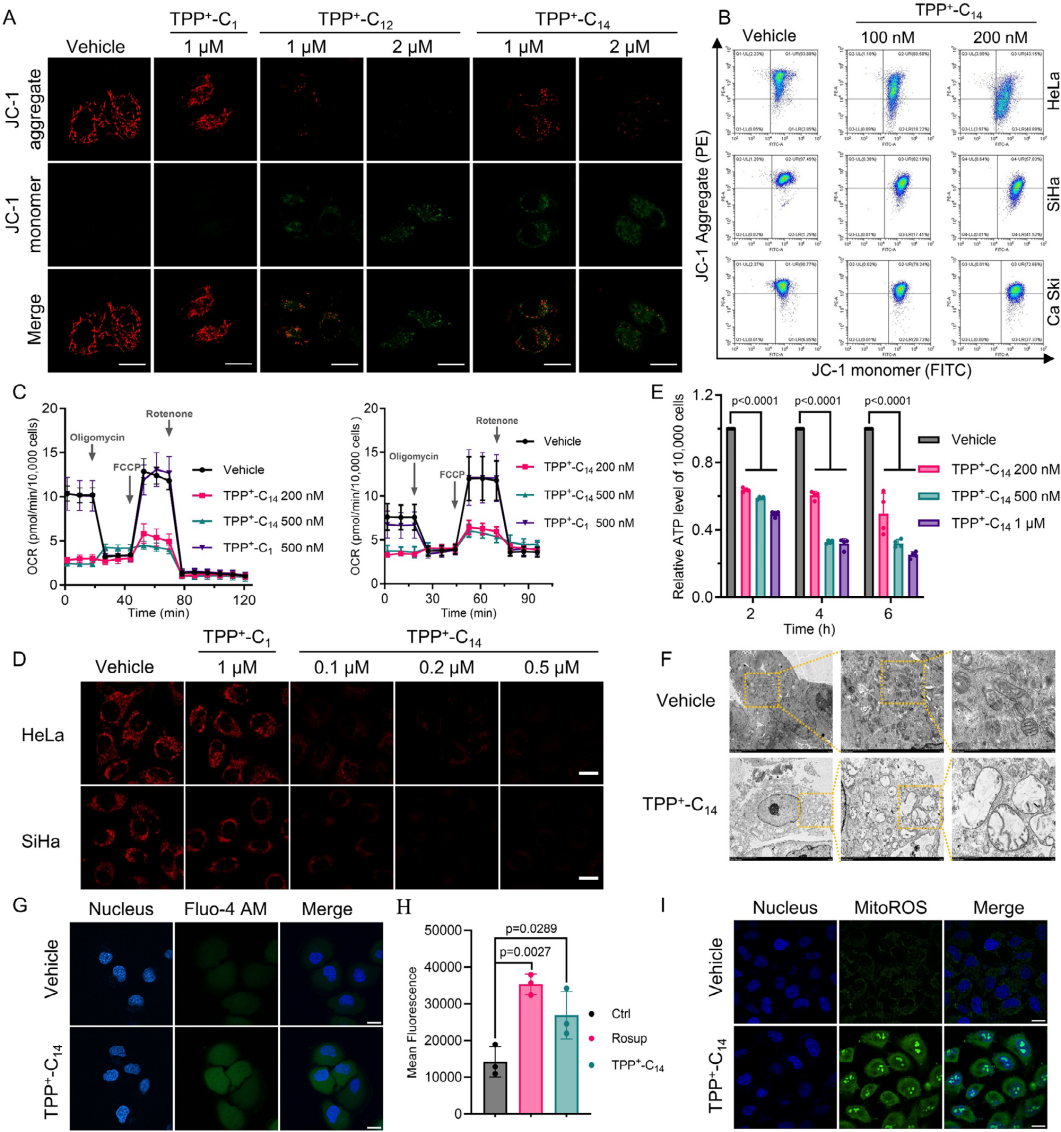
TPP^+^‐C_14_ induces mitochondrial dysfunction in cervical cancer cells. (A) JC‐1 imaging showing MMP changes. HeLa cells were treated as indicated for 6 h, followed by 30‐min staining with JC‐1. Scale bar: 20 µm. (B) Flow cytometric quantification of JC‐1 staining. Cells were treated with TPP^+^‐C_14_ for 12 h and subsequently stained with JC‐1 for analysis. (C) Oxygen consumption rate (OCR) analysis. HeLa (left) and SiHa (right) cells were treated as indicated, followed by OCR measurement. Data were normalized to the cell number of each group. (D) Intracellular ATP imaging. Cells were treated as indicated for 12 h and stained with 10 µM ATP‐Red 1 for 30 min. Scale bar: 10 µm. (E) Relative quantification of cellular ATP levels. Cells were treated as indicated, and 10 000 cells were collected for ATP measurement using the CellTiter‐Glo assay. Data are shown as mean ± SD (n = 4). (F) Representative micrographs showing mitochondrial disruption. HeLa cells were treated with 500 nm TPP^+^‐C_14_ for 24 h before TEM observation. Scale bar: 1 µm. (G) Calcium imaging. Cells were treated with 500 nm TPP^+^‐C_14_ for 24 h and stained with Fluo‐4 AM for 30 min. Scale bar: 10 µm. (H) Flow cytometric quantification of cellular ROS. HeLa cells were treated with 500 nm TPP^+^‐C_14_ or 50 µg/mL Rosup for 24 h, followed by incubation with 5 µm DCFH‐DA. Data are shown as mean ± SD (n = 3). (I) Mitochondrial ROS imaging. TPP^+^‐C_14_‐treated cells (500 nm, 24 h) were stained with MitoROS 580 for 30 min. Scale bar: 10 µm. Statistical significance in E and H was determined using one‐way ANOVA by Dunnett's multiple comparisons test.

Given the cardiolipin's critical role in maintaining mitochondrial structure and bioenergetic capacity, we investigated whether TPP^+^‐C_14_ exposure compromises mitochondrial homeostasis. Confocal imaging of JC‐1 staining, combined with quantitative flow cytometric analysis, revealed a concentration‐dependent collapse of MMP in HeLa and SiHa cells treated with TPP^+^‐C_14_ (Figure 4A,B; Figures S9). In contrast, the control compounds, including the TPP^+^ head (TPP^+^‐C_1_) and the linker (C_14_ or C_14_‐OH), showed no such effect, highlighting the structure‐specific bioactivity of TPP^+^‐C_14_ (Figure S10). Seahorse metabolic analysis demonstrated severe impairment of OXPHOS, with TPP^+^‐C_14_‐treated cells exhibiting dramatically reduced oxygen consumption rates (OCR) compared to controls (Figure 4C). This respiratory chain disruption corresponded with marked ATP depletion in TPP^+^‐C_14_‐treated cells, while TPP^+^‐C_1_, C_14,_ and C_14_‐OH had minimal impact on cellular ATP levels (Figure 4D,E; Figures S11 and S12). These indicators of mitochondrial dysfunction were supported morphologically, with ultrastructural analysis by TEM showing cells exposed to TPP^+^‐C_14_ displayed severe cristae destruction and mitochondrial swelling (Figure 4F). Additionally, TPP^+^‐C_14_ treatment induced aberrant calcium accumulation, as detected by Fluo‐4 AM staining (Figure 4G), and significantly elevated cytosolic and mitochondrial ROS, measured by DCFH‐DA and MitoROS 580, respectively (Figure 4H,I; Figure S13).

To elucidate the metabolic consequences of mitochondrial dysfunction, we conducted time‐resolved untargeted metabolomic profiling. This analysis revealed progressive metabolic reprogramming in the cells treated with TPP^+^‐C_14_, with dysregulated metabolites increasing from 59 at 12 h to 85 at 24 h, of which 46 showed consistent dysregulation across both time points (Figure S14A,B). KEGG pathway enrichment analysis of these metabolites indicated significant disruptions in amino acid metabolism and central carbon metabolism (Figure S14C). These findings demonstrate that TPP^+^‐C_14_ induces profound mitochondrial dysfunction in tumor cells, driving reprogramming of key metabolic pathways. Collectively, the evidence underscores that TPP^+^‐C_14_ exerts potent disruptive effects on mitochondrial homeostasis upon binding to cardiolipin.

### 2.5 TPP^+^‐C_14_ Selectively Induces Oncosis Rather Than Apoptosis or Autophagy

**FIGURE 5:**
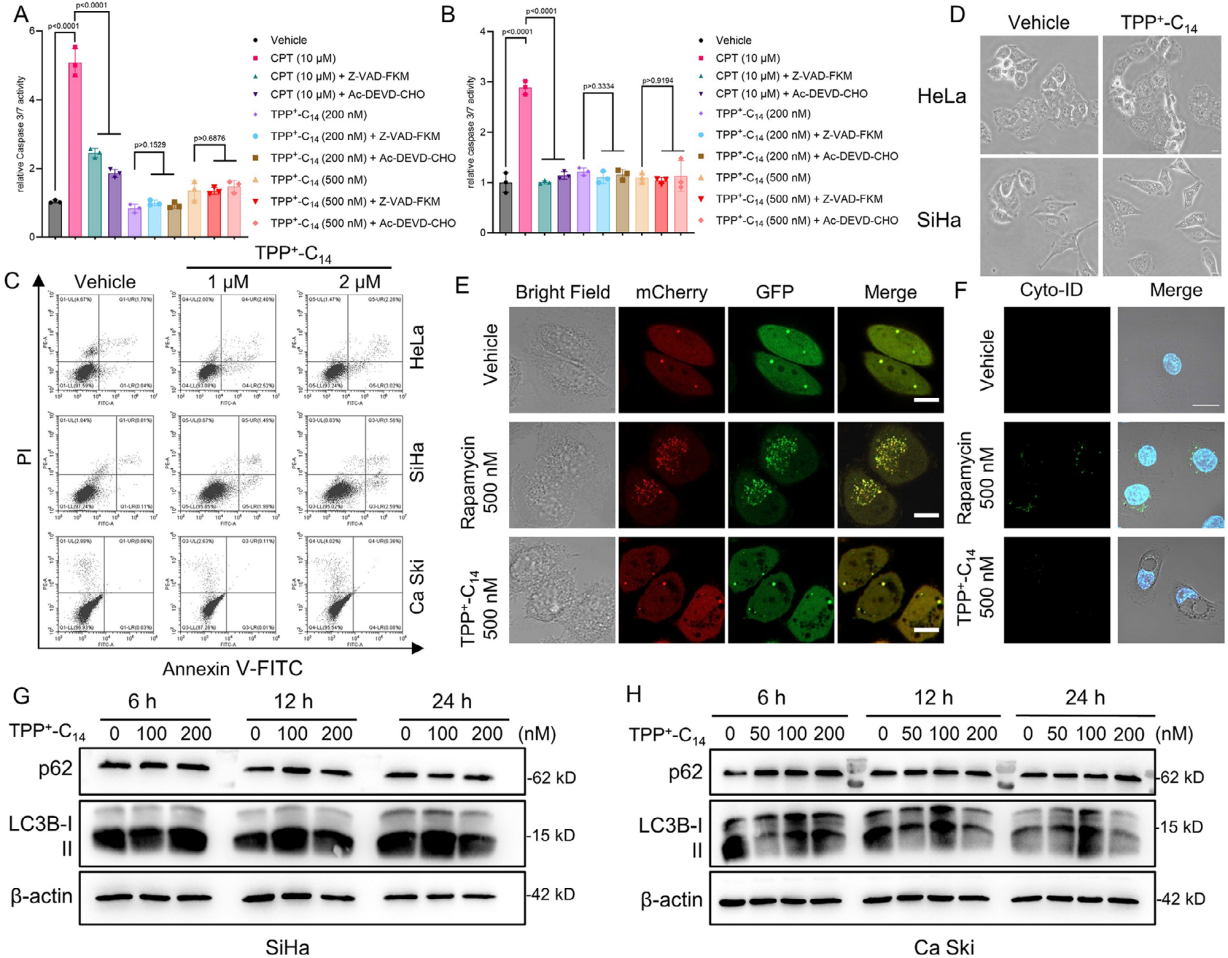
TPP^+^‐C_14_ does not induce apoptotic or autophagic cell death in cervical cancer cells. A, B) Caspase 3/7 activity assay. (A) HeLa and (B) SiHa cells were treated as indicated, and the caspase 3/7 activity was measured after incubation with 5 µm substrate for 30 min (Ex/Em: 485/515 nm). Data are shown as mean ± SD(n = 3). Statistical significance was determined using one‐way ANOVA by Dunnett's multiple comparisons test. (C) Cell apoptosis assay. Cells were treated with TPP^+^‐C_14_ (1 or 2 µm) for 48 h, and then were analyzed by Flow Cytometry after being stained with Annexin V‐FITC and propidium iodide (PI). D) Representative images showing cytoplasmic vacuolation. HeLa or SiHa cells were treated with TPP^+^‐C_14_ (500 nm) for 8 and 12 h, respectively. Scale bar: 10 µm. (E) Autophagic flux assay. The mCherry‐GFP‐LC3B‐transfected SiHa cells were treated with Rapamycin (500 nm) or TPP^+^‐C_14_ (500 nm) for 24 h. Autolysosomes: red puncta, autophagosomes: yellow puncta. Scale bar: 10 µm. (F) Cyto‐ID autophagy detection. HeLa cells treated with rapamycin (500 nm) or TPP^+^‐C_14_ (500 nm) for 24 h were stained with Cyto‐ID Green dye. (G,H) Immunoblotting of autophagy markers. Cells were treated with increasing concentrations of TPP^+^‐C_14_ for 6, 12, or 24 h, respectively, before immunoblotting analysis.

Considering the established role of mitochondrial dysfunction in driving cell death, we next sought to characterize the cell death modality triggered by TPP^+^‐C_14_. Initially, apoptosis appeared to be a likely mechanism; therefore, we first measured the caspase‐3/7 activity using a commercially available kit. However, it was found that, unlike Camptothecin (CPT), which significantly increased caspase‐3/7 activity, TPP^+^‐C_14_ did not substantially activate caspase‐3/7 activation at the tested concentrations, showing no notable difference in fluorescence signal from the control (Figure 5A,B). Flow cytometric analysis with Annexin V‐FITC/propidium iodide (PI) dual staining further revealed a negligible apoptotic cell population (<6% Annexin V^+^ cells) across all the tested TPP^+^‐C_14_ concentrations (Figure 5C), definitively ruling out apoptosis as the primary death pathway. Subsequently, we explored autophagic cell death as TPP^+^‐C_14_‐treated cells exhibited numerous cytoplasmic vacuoles suggestive of autophagosomes (Figure 5D). A series of experiments were performed to assess whether autophagy was activated upon TPP^+^‐C_14_ exposure. In the mCherry‐EGFP‐LC3B puncta formation assay, TPP^+^‐C_14_‐treated cells displayed a negligible number of autophagosome‐associated yellow puncta, comparable to vehicle‐treated cells, while rapamycin‐treated cells showed a marked increase (Figure 5E; Figure S15). Similarly, Cyto‐ID staining revealed no remarkable increase in autophagic vacuole fluorescence in TPP^+^‐C_14_‐treated cells, in stark contrast to the rapamycin group (Figure 5F). Immunoblotting further corroborated these findings, showing no increase in the LC3‐II/LC3‐I ratio or degradation of p62 in TPP^+^‐C_14_‐treated cells (Figure 5G,H; Figure S16). Collectively, these results substantiate that autophagic cell death does not underlie the antiproliferative activity of TPP^+^‐C_14_.

**FIGURE 6:**
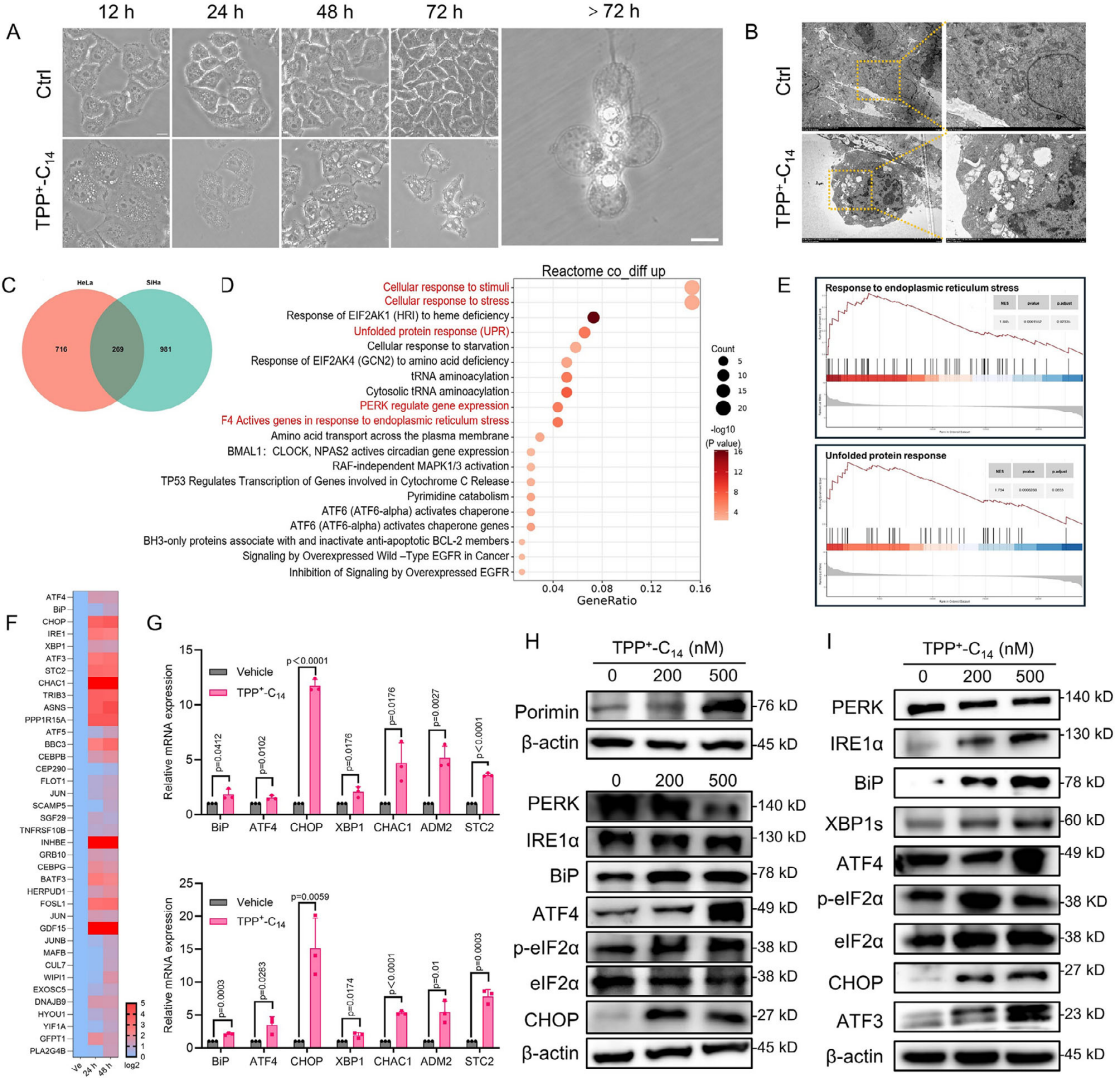
TPP^+^‐C_14_ triggers oncosis by activating the endoplasmic reticulum (ER) stress pathway. (A) Representative micrographs showing morphological alterations of HeLa cells exposed to 500 nm TPP^+^‐C_14_. Scale bar: 10 µm. (B) Representative TEM images of HeLa cells treated with TPP^+^‐C_14_ (500 nm, 24 h). Scale bar: 1 µm. (C) Venn diagram of co‐upregulated differentially expressed genes (DEGs). HeLa and SiHa cells were treated with TPP^+^‐C_14_ (500 nm, 24 h) and subjected to RNA sequencing analysis (FDR<0.05, log2 fold change>1.2). (D) Reactome pathway enrichment analysis of co‐upregulated DEGs (top 20 terms, *p*< 0.05). E) Gene set enrichment analysis (GSEA) of significant pathways (|NES|>1, *p*<0.05, adjusted *p*<0.25). (F) Heatmap of ER stress‐related genes in HeLa cells treated with TPP^+^‐C_14_ (500 nm). (G) qPCR validation of ER stress genes in HeLa (upper) and SiHa (lower) cells that were treated with TPP^+^‐C_14_ 500 nm, 48 h. Data are shown as mean ± SD(n = 3). Statistical significance was determined using a two‐tailed Student's t‐test. H) Immunoblotting of oncosis marker protein Porimin and ER stress markers of HeLa cells that were treated with TPP^+^‐C_14_ for 48 h. (I) Immunoblotting of ER stress markers in SiHa cells that were treated with TPP^+^‐C_14_ for 48 h.

Consecutive negative results prompted a comprehensive re‐evaluation of existing data, which revealed that TPP^+^‐C_14_‐treated cells exhibited distinct morphological changes, including persistent vacuolization and heterogeneous chromatin condensation (Figure 6A,B). These alterations coincided with significant ATP depletion (Figure 4D,E; Figures S11 and S12), indicative of oncosis, a distinct form of programmed cell death characterized by ATP depletion, cytoplasmic vacuolization, and organelle swelling [29, 41]. It is typically triggered by compromised plasma membrane integrity, severe ATP loss, mitochondrial dysfunction, and disrupted ion homeostasis [42]. Although the precise molecular mechanisms remain elusive, Porimin, a membrane‐associated receptor, has been identified as a key mediator of membrane‐driven oncosis [43]. Notably, dose‐dependent upregulation of Porimin was observed in TPP^+^‐C_14_‐treated HeLa and SiHa cells, whereas Porimin levels in TPP^+^C_1_‐ C_14_, or C_14_‐OH‐treated cells remained comparable to control, providing molecular evidence for oncotic pathway activation (Figure 6H; Figures S17 and S18) [44]. Similar results were observed in the TPP^+^‐C_14_‐treated tumor xenografts (Figure 2L; Figure S4B). These findings conclusively demonstrate that TPP^+^‐C_14_ preferentially induces oncotic cell death through bioenergetic collapse, distinct from the canonical apoptotic or autophagic pathways.

### 2.6 TPP^+^‐C_14_ Activates ER Stress in Cancer Cells

To shed light on the molecular events linking TPP^+^‐C_14_‐induced mitochondrial dysfunction to the eventual cell oncosis, the RNA sequencing (RNA‐seq) analysis was conducted. Co‐upregulated differentially expressed genes (DEGs) were aligned with the Reactome database and Gene Ontology (GO), revealing key pathways primarily associated with ER stress, including cellular stress responses and the unfolded protein response (UPR) (Figure 6C,D; Figure S19). Gene set enrichment analysis (GSEA) further corroborated these findings, showing significant enrichment of ER stress‐related genes (Figure 6E; Figure S20). This effect was further validated by sequential RNA‐seq analysis of HeLa cells treated with TPP^+^‐C_14_ for 24 and 48 h (Figure S21), in which representative genes, such as BiP, ATF4, and CHOP, were notably increased at both time points (Figure 6F). When ER stress occurs, the ER chaperone protein BiP dissociates from PERK (PKR‐like ER kinase), enabling PERK dimerization and autophosphorylation, which activates downstream signaling pathways to initiate the UPR [45, 46]. TPP^+^‐C_14_ treatment induced ER stress as evidenced by elevated BiP transcripts and protein levels in both HeLa and SiHa cells (Figure 6G−I). Following BiP induction, PERK levels decreased, while phosphorylated eIF2α (p‐eIF2α), ATF4, and CHOP were upregulated, confirming the activation of the PERK/eIF2α/ATF4/CHOP pathway. Severe or prolonged ER stress, driven by TPP^+^‐C_14_‐induced mitochondrial dysfunction, resulted in irreversible cellular damage and cell oncosis.

## 3 Conclusions

The TPP^+^ cation is distinguished by its lipophilicity, positive charge, and consequent mitochondrial targeting. Prior studies have highlighted its critical role in enhancing the anticancer efficacy of drug payloads by facilitating their selective accumulation in mitochondria. Typically, TPP^+^ is arbitrarily considered biologically inert. However, its alkyl derivatives have been reported to exhibit antiproliferative effects, with their structure‐activity relationship and mechanism of action remaining elusive. In this study, we first systematically investigated the in vitro antiproliferative activity of the synthetic alkylTPP^+^ derivatives with chain lengths varying from C_1_ to C_18_. A distinct structure‐activity relationship emerged, where biological activity shows progressive enhancement with increasing alkyl chain elongation up to 9 carbons, beyond which a plateau effect becomes evident. Subsequently, we evaluated the in vivo activity using TPP^+^‐C_14_ as the representative, which showed tolerable toxicity to mice and excellent anticancer efficacy.

**FIGURE 7:**
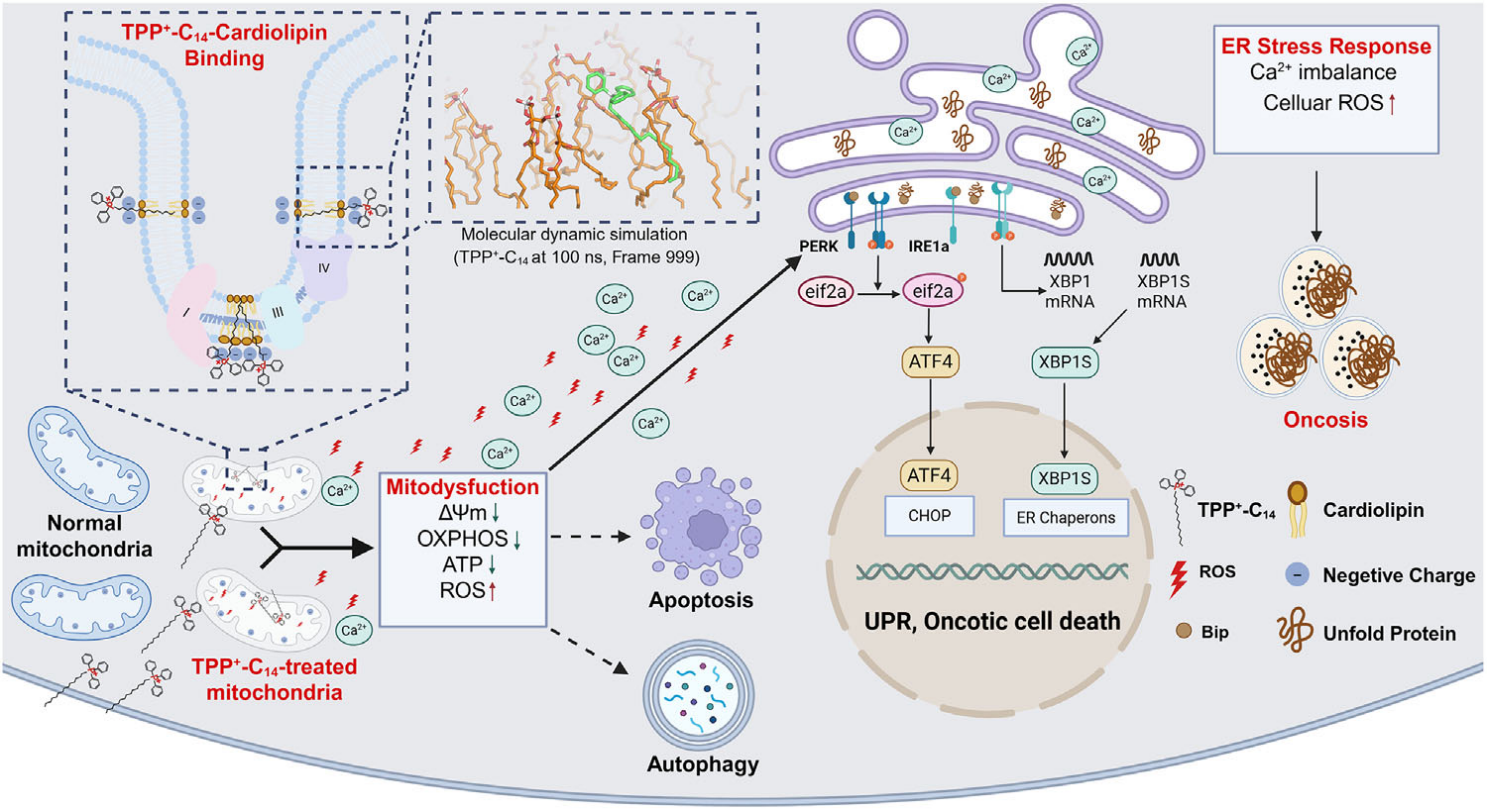
Schematic illustration demonstrating the mechanism of action of TPP^+^‐C_14_ in cancer cells. TPP^+^‐C_14_ preferentially accumulates in mitochondria and selectively binds to cardiolipin through electrostatic and hydrophobic interactions. This binding disrupts cardiolipin function, leading to MMP collapse, oxidative phosphorylation (OXPHOS) inhibition, ATP depletion, ROS enhancement, and Ca^2^^+^ homeostasis imbalance. These mitochondrial dysfunctions predominantly induce oncosis in cancer cells by activating the PERK/eIF2α/ATF4/CHOP pathway in ER stress.

Simple alkylation of TPP^+^ enables potent tumor suppression both in vitro and in vivo, prompting an investigation into its mechanism of action. Notably, long‐alkyl‐chain TPP^+^ derivatives exhibit enhanced efficacy, suggesting their biological activity arises from embedding into the mitochondrial membrane, particularly the IMM. This hypothesis directed our attention to cardiolipin, a unique diphosphatidylglycerol phospholipid predominantly found in the IMM, essential for mitochondrial membrane organization, ETC stability, energy production, and cell death regulation. With its four acyl chains and a negatively charged headgroup, cardiolipin likely forms both hydrophobic and electrostatic interactions with alkylated TPP^+^ cations. According to the binding affinity measurements, competitive binding assays, and MD simulations, it was confirmed that alkyTPP^+^ derivatives display distinct chain length‐dependent binding specificity for cardiolipin, with TPP^+^‐C_14_ showing the highest affinity. This binding trend aligns with the observed structure‐activity relationship, suggesting that the antitumor effects of alkyTPP^+^ derivatives are mediated through targeting cardiolipin. Disruption of cardiolipin function by binding to TPP^+^‐C_14_ leads to MMP collapse, increased ROS, calcium dysregulation, ATP depletion, and metabolic reprogramming, ultimately culminating in mitochondrial dysfunction (Figure 7). Interestingly, while mitochondrial damage typically triggers apoptosis or autophagy, TPP^+^‐C_14_ predominantly induces oncosis in cancer cells by activating the PERK/eIF2α/ATF4/CHOP pathway in ER stress.

Using mitochondrial targeting ligands, such as TPP^+^, to develop drug conjugates or probes has emerged as a cutting‐edge paradigm in cancer theranostics. Unlike prior studies that utilized TPP^+^ primarily as a mitochondrial delivery vehicle, this study focuses on investigating the intrinsic biological activity of TPP^+^ and its mechanistic basis. Our findings demonstrate that alkylation of TPP^+^ confers potent anticancer efficacy in a cardiolipin‐dependent manner. By targeting cardiolipin, alkylTPP^+^ derivatives, notably TPP^+^‐C_14_, disrupt mitochondrial homeostasis and induce oncosis via ER stress pathways. These insights redefine the role of TPP^+^ in mitochondria‐targeted theranostics and offer a promising foundation for developing next‐generation anticancer therapies that modulate mitochondrial function precisely. However, the molecular interplay between mitochondrial dysfunction and ER stress, as well as the relationship between cardiolipin and oncosis, remains poorly elucidated and warrants further investigation.

## 4 Experimental Section

### 4.1 Liposome Preparation and Characterization

The liposomal formulation was prepared using thin‐film hydration followed by extrusion. Briefly, lecithin (10 mg), cholesterol (2.2 mg), DSPE‐mPEG2000 (2.2 mg), and TPP^+^‐C_14_ (1.0 mg) were dissolved in 3 mL chloroform in a 25 mL round‐bottom flask. The solvent was evaporated under reduced pressure at 35 °C using a rotary evaporator to form a thin lipid film. The film was then hydrated with 1 mL of PBS (10 mm, pH 7.4) and gently vortexed for 30 min, followed by shaking for 60 min at room temperature. The resulting milky suspension was extruded nine times through a 100 nm polycarbonate membrane using an Avanti mini‐extruder to yield uniform liposomal TPP^+^‐C_14_. Blank liposomes were prepared similarly, omitting TPP^+^‐C_14_. For DiD‐labeled liposomal TPP^+^‐C_14_, 0.05 mg of DiD was included in the chloroform solution during the initial lipid dissolution step. The size distribution and zeta potential size of the liposomes were characterized using dynamic light scattering (DLS, Zetasizer Nano ZS90, Malvern), and their morphology was assessed by transmission electron microscopy (TEM, HT7800, Hitachi). Thermal analysis of lyophilized liposome samples was performed using a differential scanning calorimeter (TA DSC250, USA). The samples were weighed (5−10 mg), placed in aluminum pans, firmly pressed, and heated from 0 °C to 100 °C at a rate of 10 °C /min, with an empty aluminum box as a control.

### 4.2 Cell Lines

All cell lines, including HeLa (RRID: CVCL_0030), SiHa (RRID: CVCL_0032), Ca Ski (RRID: CVCL_1100), HeLa 229 (RRID: CVCL_1276), and NIH/3T3 (RRID: CVCL_0594), were purchased from Wuhan Pricella Biotechnology Co., Ltd. and verified to be free of contamination. Cells were maintained in Dulbecco's modified Eagle's medium (Viva Cell Bioscience), supplemented with 10% (v/v) fetal bovine serum, 100 U mL^−1^ penicillin, and 100 µg mL^−1^ streptomycin, and were cultured in a humid atmosphere containing 5% CO_2_ at 37 °C.

### 4.3 Cyto‐ID Autophagy Detection

Tumor cells were grown on tissue culture‐treated plates to 50% confluence and treated with rapamycin or TPP^+^‐C_14_ for 24 h. Afterward, cells were washed twice with the assay buffer and stained with 100 µL of dual detection reagent (Enzo Life, #ENZ‐51031) for 30 min at 37 °C in the dark. Cells were then gently washed with assay buffer and were observed using confocal microscopy (Zeiss LSM800).

### 4.4 Mitochondrial Targeting Analysis

The treated tumor cells were harvested, centrifuged at 600 g for 5 min at 4 °C, and the supernatant was discarded. Mitochondria were then isolated using a mitochondrial isolation kit (Beyotime, #C3601) according to the manufacturer's protocol. The isolated mitochondria were treated with methanol and analyzed by high‐performance liquid chromatography (Agilent 1260). For living cell imaging, cells were incubated with TPP^+^‐C_14_‐NBD and MitoTracker Deep Red (Beyotime, #C1034) for 30 min, followed by fluorescence imaging using a confocal microscope (LSM800, Zeiss).

### 4.5 Cellular ATP Imaging

Tumor cells were grown in confocal dishes for adherence and then treated with TPP^+^‐C_14_ or TPP^+^‐C_1_ as indicated for 12 h. Afterward, cells were incubated with ATP Red1 (GlpBio, #GC30265) working solution at room temperature in the dark for 30 min, and the fluorescence imaging was performed using a confocal microscope (LSM800, Zeiss).

### 4.6 Oxygen Consumption Rate (OCR) Analysis

Mitochondrial respiratory capacity was assessed by using Agilent Seahorse XF Cell Mito Stress Test Kit (Agilent, #103015‐100) according to the manufacturer's instructions. Briefly, tumor cells (15 000 cells/well) were seeded into XFe‐24 well cell culture plates and incubated overnight. Then, cells were treated with TPP^+^‐C_1_ (500 nm, 24 h), or TPP^+^‐C_14_ (200 or 500 nm, 24 h). After treatments, the medium was replaced with Seahorse assay medium supplemented with 10 mm glucose, 1 mm Pyruvate, and 2 mm l‐glutamine, and then incubated at 37 °C without CO_2_ for 60 min. OCR was measured using a Seahorse XFe24 Analyzer (Agilent) under basal conditions and after sequential injection of oligomycin (1.5 µm), FCCP (2 µm), and rotenone/antimycin A (0.5 µM). Data were normalized according to the cell number of each group.

### 4.7 Biolayer Interferometry (BLI)

The binding interaction between alkylTPP^+^ derivatives and cardiolipin (Aladdin, #C130308) was quantified with a 16‐channel Octet RH16 system (Sartorius). Aminopropylsilane (APS) biosensors were pre‐wetted in PBS for 10 min and then immobilized with 1 mm cardiolipin. These biosensors were exposed to serial two‐fold dilutions of alkylTPP^+^ derivatives or 10‐N‐nonyl acridine orange (NAO), starting from 100 µm in Buffer I (1× PBS, pH 7.4, 0.1% DMSO), for 60 s association, and then transferred to fresh Buffer II (1× PBS, pH 7.4, 0.05% Tween) for 60 s dissociation. Kinetic binding data were analyzed using a 1:1 binding model to determine the affinity constant (KD), association rate constant (K_on_), and dissociation rate constant (K_off_).

### 4.8 Competitive Cell Imaging with NAO

Tumor cells were seeded in confocal dishes and allowed to adhere overnight at 37 °C. The cells were treated with NAO alone or co‐incubated with NAO and TPP^+^‐C_1_ or TPP^+^‐C_14_ together. The co‐incubation group was subjected to two sequential treatment protocols: incubation with 500 nm NAO for 30 min, followed by 1 µm TPP^+^‐C_1_ or TPP^+^‐C_14_ for an additional 30 min, or pretreated with 1 µm TPP^+^‐C_1_ or TPP^+^‐C_14_ for 30 min, followed by 500 nm NAO for another 30 min. After treatment, fluorescence images were captured using a confocal laser scanning microscope (LSM800, Zeiss)

### 4.9 MD Simulations

Through using CHARMM scripts provided by the CHARMM‐GUI server Membrane Builder, the bilayer membrane model was built from a DOPC (dioleoyl phosphatidylcholine) and TLCL (tetralinoleoyl cardiolipin, ‐2e charge) randomly distributed bilayer lipid membrane. The top and bottom leaflets of lipid have the same composition, containing 102 DOPC and 26 TLCL molecules in each layer. The membrane model containing 20% CL was constructed according to the previous study by Mohammadyani et al., The aqueous phase has a 22.5 Å thickness on each side of the membrane. The net charge was neutralized by Cl^−^, and a 0.15 m concentration of NaCl was kept in the aqueous phase. The prepared system with a membrane in the middle and water phases on both sides along the z‐axis was a 102 × 102 × 85 Å^3^ rectangular. Topology files of TPP^+^‐C_1_ and TPP^+^‐C_14_ were obtained from the CGenFF server. To better describe the charge distribution of TPP^+^‐C_1_ and TPP^+^‐C_14_, DFT calculation was carried out with Gaussian 16 to get the advanced restrained electrostatic potential (RESP2) atomic partial charges with B3LYP‐D3(BJ) functional and def2‐TZVP basis set. Then, the RESP2 atomic charge replaced the previous charge generated by CGenFF.

Atomistic MD simulations were performed using the GROMACS 2024.4 package with the CHARMM36 forcefield, and CGenFF forcefield, and the TIP3P explicit water model. For the membrane system, energy minimization and equilibration steps were performed according to the CHARMM‐GUI guidelines: first, steepest‐descent minimization for 5000 steps; second, two rounds of canonical ensemble (NVT) equilibration for 125 ps each with a 1 fs timestep; third, one round of isothermal‐isobaric (NPT) ensemble equilibration for 125 ps with a 1 fs timestep; and finally, three rounds of NPT ensemble equilibration for 125 ps each with a 2 fs timestep. Position and dihedral restraints on the phospholipid were gradually released to 0. The V‐rescale thermostat was used to control the temperature, whereas the C‐rescale barostat was used in semi‐isotropic pressure coupling. The system achieved constant temperature (310.15 K) and constant pressure (1.0 bar). H‐bonds were constrained using the default linear constraint (LINCS) solver algorithm. Periodic boundary conditions (PBC) were applied in all directions. The Particle‐Mesh Ewald (PME) method was employed to deal with long‐range interactions, and a 1.2 nm cutoff was used for van der Waals interactions. Unrestrained MD simulations were run for 100 ns for the membrane system using a time step of 2 fs to fully relax the membrane structure. Then, TPP^+^‐C_1_ or TPP^+^‐C_14_ was inserted into the system by replacing water molecules randomly. An additional chloride ion was incorporated into the system subsequently through stochastic substitution of a water molecule to maintain charge neutrality. After that, three independent 100 ns production MD simulations were performed for each probe‐phospholipid bilayer system. After the simulation, trajectories and interactions between probe and lipids were analyzed. Trajectory visualization was performed using PyMOL 3.1 software package (Schrödinger, LLC).

### 4.10 Quantitative RT‐PCR (qPCR)

Total RNA was purified from tumor cells using the universal RNA extraction kit (Accurate Biology, #AG21017), following the manufacturer's recommended protocol. Reverse transcription was conducted with the reverse transcriptase kit (Accurate Biology) to synthesize complementary DNA (cDNA). Quantitative polymerase chain reaction (qPCR) was performed using the SYBR Green Pro Taq HS Premix qPCR Kit (Accurate Biology, AG11701) on a real‐time quantitative PCR system (QuantStudio 5, Thermo Fisher Scientific). The primers are shown in Table S3.

### 4.11 Untargeted Metabolomics

Untargeted metabolomics was conducted by KaiTai Biotechnology (Hangzhou, China) as follows: Cell samples were transferred into a 2 mL centrifuge tube along with 100 mg of glass beads. A mixed solution of acetonitrile (ACN), methanol, and H_2_O in a 2:2:1 ratio (V/V/V) was then added, totaling 1000 µL, and the mixture was vortexed for 30 s. The centrifuge tube was subsequently placed into a matching 2 mL adapter and immersed in liquid nitrogen for rapid freezing for 5 min. Afterward, the tube was removed and allowed to thaw at room temperature. It was then reinserted into the adapter and ground in a tissue grinder at 60 Hz for 2 min. This freezing and grinding procedure was repeated two additional times. Following the final grinding, the samples were centrifuged at 12 000 rpm for 10 min at 4 °C, and the supernatant was carefully collected and transferred to a new 2 mL centrifuge tube for concentration and drying. To redissolve the dried sample, 300 µL of a solution containing acetonitrile and 2‐amino‐3‐(2‐chloro‐phenyl)‐propionic acid (4 ppm) prepared with 0.1% formic acid (1:9, V/V) was added. The resulting supernatant was filtered through a 0.22 µm membrane and transferred to a detection bottle for subsequent LC‐MS analysis.

The LC analysis was performed on a Vanquish UHPLC System (Thermo Fisher Scientific, USA). Chromatography was carried out with an ACQUITY UPLC HSS T3 (2.1 × 100 mm, 1.8 µm) (Waters, Milford, MA, USA). The column was maintained at 40°C. The flow rate and injection volume were set at 0.3 mL/min and 2 µL, respectively. For LC‐ESI (+)‐MS analysis, the mobile phases consisted of (B2) 0.1% formic acid in acetonitrile (v/v) and (A2) 0.1% formic acid in water (v/v). Separation was conducted under the following gradient: 0∼1 min, 8% B2;1∼8 min, 8%∼98% B2; 8∼10 min,98% B2; 10∼10.1 min, 98%∼8% B2; 0.1∼12 min, 8% B2. For LC‐ESI (‐)‐MS analysis, the analytes were carried out with (B3) acetonitrile and (A3) ammonium formate (5 mm). Separation was conducted under the following gradient: 0∼1 min, 8% B3; 1∼8 min, 8%∼98% B3; 8∼10 min, 98% B3; 10∼10.1 min, 98%∼8% B3; 10.1∼12 min, 8% B3. Mass spectrometric detection of metabolites was performed on an Orbitrap Exploris 120 (Thermo Fisher Scientific, USA) with an ESI ion source. Simultaneous MS1 and MS/MS (Full MS‐ddMS2 mode, data‐dependent MS/MS) acquisition was used. The parameters were as follows: sheath gas pressure, 40 arb; aux gas flow, 10 arb; spray voltage, 3.50 and −2.50 kV for ESI (+) and ESI (‐), respectively; capillary temperature, 325 °C; MS1 range, m/z 100–1000; MS1 resolving power, 60 000 FWHM; number of data dependant scans per cycle, 4; MS/MS resolving power, 15 000. Two different multivariate statistical analysis models, unsupervised and supervised, were applied to discriminate the groups (PCA; PLS‐DA; OPLS‐DA) by the R ropls (v1.22.0) package. The statistical significance of the p‐value was obtained by a statistical test between groups. Finally, combined with *p*‐value, VIP (OPLS‐DA variable projection importance), and FC (multiple of the difference between groups) to screen biomarker metabolites. By default, when the *p*‐value <0.05 and VIP value > 1, we think that the metabolite was considered to have significant differential expression.

### 4.12 Pathway Analysis

Differential metabolites were subjected to pathway analysis by MetaboAnalyst, which combines results from powerful pathway enrichment analysis with the pathway topology analysis. The identified metabolites in metabolomics were then mapped to the KEGG pathway for biological interpretation of higher‐level systemic functions. The metabolites and corresponding pathways were visualized using the KEGG Mapper tool.

### 4.13 Immunoblotting

The tumor cells treated with TPP^+^‐C_14,_ as indicated were lysed in RIPA buffer containing a protease inhibitor cocktail, and protein concentrations were quantified using a BCA protein assay kit (GlpBio, GK10009). Protein samples were denatured at 100 °C for 10 min in a loading buffer containing 150 mm DTT. Equal amounts of protein (30 µg per sample) were resolved on 4%–15% SDS‐PAGE gels (Bio‐Rad) and then transferred to a 0.45 µm polyvinylidene difluoride (Millipore, #IPVH08130) membrane.

Primary antibodies were applied at a 1:1000 dilution overnight at 4 °C after saturation in 3% BSA. Antibodies, including CHOP (#2895T,), ATF‐4 (#11815S), ATF‐3 (#18665S), XBP‐1s (#12782S), IRE1α (#3294T), BiP (#3177T), PERK (#5683T), eIF2α (#5324T), p‐eIF2α (#3597S), p62 (#5114), LC3B (#2775S), and β‐actin (#3700S), were purchased from Cell Signaling Technology. Porimin antibody (#377189) was obtained from Santa Cruz Biotechnology. Another β‐actin antibody (#20536‐1‐AP) was purchased from Proteintech. After incubation, the membranes were treated with HRP‐conjugated secondary antibodies (1:3000 dilution) for 1 h at room temperature. Proteins were visualized using Western Lightning Chemiluminescence Reagent Plus (PerkinElmer), and images were captured using the ChemiDoc Touch Imaging System (Bio‐Rad).

### 4.14 RNA Sequencing

The tumor cells, treated as indicated, were harvested and lysed with Trizol reagent (Accurate Biology) for RNA extraction. RNA sequencing and data analysis were performed by KaiTai Biotechnology (Hangzhou, China). Total RNA was extracted using the RNAprep Pure Cell/Bacteria Kit (Tiangen) and further purified with the RNAclean XP Kit (Beckman Coulter) and the RNase‐Free DNase Set (Qiagen) to remove genomic DNA contamination. Library preparation was performed using the U‐mRNAseq Library Prep Kit (Kaitai Bio) in combination with the Ribo‐off rRNA Depletion Kit (Vazyme) for mRNA enrichment. The prepared libraries were pooled and sequenced on an Illumina NovaSeq platform, generating 150‐bp paired‐end reads. Raw sequencing reads were quality‐filtered using Fastp 0.23.0 to trim low‐quality bases and reads shorter than 50 bp. The reference genome and gene annotation files for Homo sapiens were retrieved from Ensembl v101. The cleaned reads were aligned to the reference genome using HISAT2 (v2.2.1, http://daehwankimlab.github.io/hisat2/). Mapped reads were assembled using StringTie (v1.3.6) via a reference‐based approach, and transcript abundance was quantified as Fragments Per Kilobase Million Mapped Reads (FPKM), calculated as: FPKM = 10^6^ × F / (N × 10^−3^), where F represents the number of fragments assigned to a specific gene, N is the total number of mapped reads, and L is the gene length in kilobases.

Differential expression analysis was performed using edgeR, with thresholds set at FDR <0.05 and |log2 fold change| > 1.2 to identify differentially expressed genes. Functional enrichment analysis, including Gene Ontology (GO) and Reactome pathway analysis, was conducted to determine significantly enriched biological functions and pathways (*p* <0.05). GSEA was performed using the R package cluster Profiler 4.14.6. Normalized expression values were ranked based on log_2_ fold changes from differential expression analysis. The reference genome annotation file for Homo sapiens (Ensembl v101) was used to map gene identifiers to Entrez IDs for pathway enrichment analysis. Curated gene sets, including KEGG pathways, Reactome pathways, and GO terms, were retrieved from the Molecular Signatures Database. Statistical significance was determined via 1000 phenotype label permutations, with gene sets meeting FDR <0.25 and nominal *p*‐value <0.05 considered significantly enriched. Enrichment plots generated by clusterProfiler displayed the normalized enrichment score (NES), FDR‐adjusted p‐values, and leading‐edge gene distributions for each enriched gene set.

### 4.15 Animal Study

Animal experiments were performed following the National Institute Guide for the Care and Use of Laboratory Animals, with the protocols (No. 230011) approved by the Animal Ethics Committee of the School of Pharmaceutical Sciences, Shandong University. The cervical tumor xenograft model was established in 6‐week‐old female BALB/c nude mice by subcutaneous injection of HeLa cells (2 × 10^6^ cells in 100 µL PBS) into the axillary region. When the tumor volume reached ∼100 mm^3^, mice were randomly grouped (n  =  6 per group) and were treated as indicated. Tumor dimensions were measured every three days using a caliper, and volumes were calculated using the formula: volume  =  (L × W^2^)/2, where L is the longest diameter, and W is the shortest. Body weight was recorded every three days throughout the treatment period.

For the biodistribution study, five nude mice bearing the HeLa cell‐derived xenograft (∼200 mm^3^) were administered DiD‐labeling liposomal TPP^+^‐C_14_, which contains 0.5 mg/kg TPP^+^‐C_14_ and 0.05 mg/kg DiD, via tail vein injection. After 24 h, the in vivo imaging was performed using the IVIS Spectrum CT imaging system. Subsequently, major organs and tumor tissues were harvested for ex vivo imaging.

To assess the safety profile of TPP^+^‐C_14_, a toxicity study was conducted in ICR mice (n = 6 mice per group). At the end of the study, blood samples were collected via eyeball for hematological and comprehensive metabolic panel analysis (n = 3). In the meantime, major organs were also harvested for histopathological examination.

### 4.16 Statistical Analysis

Statistical analysis was performed using GraphPad Prism 10.2.3. Data are presented as mean values ± SD, *n*  =  biological replicates or independent nanoparticle sample replicates. One‐way ANOVA with Dunnett's multiple comparisons test or two‐tailed Student's t‐test was used to calculate the *p‐value*.

## Conflicts of Interest

The authors declare no conflicts of interest.

## Supporting information




**Supporting File**: advs73998‐sup‐0001‐SuppMat.docx

## Data Availability

The data that support the findings of this study are available from the corresponding author upon reasonable request.
