# Supplementary material for: Alkyltriphenylphosphonium Binding to Cardiolipin Triggers Oncosis in Cancer Cells
Source: Adv Sci (Weinh). 2026 Jan 25;13(18):e10237. doi: 10.1002/advs.202510237 (PMC13042706; doi:10.1002/advs.202510237)
Supplement: Supplementary file 1 — Supporting File: advs73998‐sup‐0001‐SuppMat.docx [file ADVS-13-e10237-s001.docx]

Supporting Information

**Alkyltriphenylphosphonium Binding to Cardiolipin Triggers Oncosis in Cancer Cells**

*Jin Li, Hang Zheng, Yuxing Lin, Ziwei Zhao, Yijia Mai, Zhangrong Lou, Qiang Liu, Zhao Ma*, Chengjun Wu**

J. Li, Z. Lou, Q. Liu, C. Wu

Faculty of Medicine, Dalian University of Technology, Dalian, Liaoning 116024, China

E-mail: wcj5532@126.com

H. Zheng, Z. Zhao, Y. Mai, Z. Ma

Department of Medicinal Chemistry, Key Laboratory of Chemical Biology (MOE), Shandong Key Laboratory of Druggability Optimization and Evaluation for Lead Compounds, School of Pharmaceutical Sciences, Cheeloo College of Medicine, Shandong University, Jinan, Shandong 250012, China

E-mail: [mazhao@sdu.edu.cn](mailto:mazhao@sdu.edu.cn)

Y. Lin

Institute of Medical Sciences, The Second Hospital, Cheeloo College of Medicine, Shandong University, Jinan, Shandong 250033, China.

C. Wu

School of Life and Health Sciences, Qingdao Central Hospital, University of Health and Rehabilitation Sciences, Qingdao, Shandong 266113, China

**Chemical synthesis.**

**Scheme S1.** Synthetic route of TPP^+^-C_n_.

**General synthetic method of TPP^+^-C_n_.** Triphenylphosphine (525 mg, 2 mmol) and the corresponding n-alkyl Iodide (2 mmol) were dissolved in MeCN (30 mL) and refluxed for 72 h. The solvent was removed under vacuum, and the residue was purified using a silica column with EA/MeOH as the eluent to afford the light yellow solid.

**TPP^+^-C_1_.** ^1^HNMR (DMSO-*d_6_*) δ 7.93–7.88 (m, 3 H), 7.80–7.74 (m, 12 H), 3.19 (d, 3 H, J=14.4 Hz). ^13^CNMR (DMSO-*d_6_*) δ 135.3, 135.3, 133.8, 133.7, 130.7, 130.5, 120.8, 119.9, 8.1, 7.5. ESI-HRMS [M-I]^+^ m/z calcd for C_19_H_18_P^+^, 277.11, found 277.11. HPLC purity 99.8%, t_R_=14.550 min, C_8_ reversed-phase column (5 μm, 250×4.60 mm, Phenomenex), Water: MeCN from 100:0 to 0:100, 1.0 mL/min.

**TPP^+^-C_2_.** ^1^HNMR (DMSO-*d_6_*) δ 7.94–7.76 (m, 15 H), 3.67–3.58 (m, 2 H), 1.27 (m, 3 H). ^13^CNMR (DMSO-*d_6_*) δ 135.4, 135.4, 134.2, 134.1, 130.8, 130.7, 119.2, 118.3, 15.2, 14.7, 6.8, 6.7. ESI-HRMS [M-I]^+^ m/z calcd for C_20_H_20_P^+^, 291.1297, found 291.1296. HPLC purity 99.8%, t_R_=15.091 min, C_8_ reversed-phase column (5 μm, 250×4.60 mm, Phenomenex), Water: MeCN from 100:0 to 0:100, 1.0 mL/min.

**TPP^+^-C_3_****.** ^1^HNMR (DMSO-*d_6_*) δ 7.93–7.76 (m, 15 H), 3.62–3.55 (m, 2 H), 1.61–1.53 (m, 2 H), 1.10-1.07 (m, 3 H). ^13^CNMR (DMSO-*d_6_*) δ 135.4, 135.4, 134.1, 134.0, 130.8, 130.7, 119.5, 118.6, 22.7, 22.2, 16.4, 16.3, 15.5, 15.3. ESI-HRMS [M-I]^+^ m/z calcd for C_21_H_22_P^+^, 305.1454, found 305.1453. HPLC purity 99.7%, t_R_=16.039 min, C_8_ reversed-phase column (5 μm, 250×4.60 mm, Phenomenex), Water: MeCN from 100:0 to 0:100, 1.0 mL/min.

**TPP^+^-C_4_.** ^1^HNMR (DMSO-*d_6_*) δ 7.93–7.76 (m, 15 H), 3.62–3.56 (m, 2 H), 1.50–1.48 (m, 4 H), 0.91 (m, 3 H). ^13^CNMR (DMSO-*d_6_*) δ 135.4, 135.4, 134.1, 134.0, 130.8, 130.7, 119.5, 118.6, 24.3, 24.3, 23.7, 23.5, 20.8, 20.3, 13.8. ESI-HRMS [M-I]^+^ m/z calcd for C_22_H_24_P^+^, 319.1610, found 319.1609. HPLC purity 99.8%, t_R_=16.926 min, C_8_ reversed-phase column (5 μm, 250×4.60 mm, Phenomenex), Water: MeCN from 100:0 to 0:100, 1.0 mL/min.

**TPP^+^-C_5_.** ^1^HNMR (DMSO-*d_6_*) δ 7.93–7.76 (m, 15 H), 3.63-3.35 (m, 2 H), 1.58–1.41 (m, 4 H), 1.35–1.26 (m, 2 H), 0.83 (t, 3 H, J=7.2 Hz). ^13^CNMR (DMSO-*d_6_*) δ 135.4, 135.4, 134.1, 134.0, 130.8, 130.7, 119.5, 118.6, 32.5, 32.3, 21.9, 21.9, 21.8, 20.9, 20.4, 14.1. ESI-HRMS [M-I]^+^ m/z calcd for C_23_H_26_P^+^, 333.1767, found 333.1765. HPLC purity 99.8%, t_R_=17.770 min, C_8_ reversed-phase column (5 μm, 250×4.60 mm, Phenomenex), Water: MeCN from 100:0 to 0:100, 1.0 mL/min.

**TPP^+^-C_6_.** ^1^HNMR (DMSO-*d_6_*) δ 7.93–7.76 (m, 15 H), 3.63-3.56 (m, 2 H), 1.54–1.45 (m, 4 H), 1.28–1.19 (m, 4 H), 0.85 (t, 3 H, J=6.8 Hz). ^13^CNMR (DMSO-*d_6_*) δ 135.4, 135.4, 134.1, 134.0, 130.8, 130.7, 119.5, 118.6, 30.8, 30.1, 29.9, 22.3, 22.2, 22.2, 20.9, 20.4, 14.3. ESI-HRMS [M-I]^+^ m/z calcd for C_24_H_28_P^+^, 347.1923, found 347.1922. HPLC purity 99.7%, t_R_=18.606 min, C_8_ reversed-phase column (5 μm, 250×4.60 mm, Phenomenex), Water: MeCN from 100:0 to 0:100, 1.0 mL/min.

**TPP^+^-C_7_.** ^1^HNMR (DMSO-*d_6_*) δ 7.92–7.78 (m, 15 H), 3.62–3.55 (m, 2 H), 1.54-1.45 (m, 4 H), 1.31–1.17 (m, 6 H), 0.85 (t, 3 H, J=6.8 Hz). ^13^CNMR (DMSO-*d_6_*) δ 135.4, 135.3, 134.1, 134.0, 130.8, 130.7, 119.5, 118.6, 31.4, 30.3, 30.2, 28.3, 22.4, 22.2, 22.2, 20.9, 20.4, 14.4. ESI-HRMS [M-I]^+^ m/z calcd for C_25_H_30_P^+^, 361.2080, found 361.2079. HPLC purity 99.8%, t_R_=19.417 min, C_8_ reversed-phase column (5 μm, 250×4.60 mm, Phenomenex), Water: MeCN from 100:0 to 0:100, 1.0 mL/min.

**TPP^+^-C_8_.** ^1^HNMR (DMSO-*d_6_*) δ 7.93–7.75 (m, 15 H), 3.62–3.5 (m, 2 H), 1.54-1.42 (m, 4 H), 1.27–1.21 (m, 8 H), 0.85 (t, 3 H, J=6.8 Hz). ^13^CNMR (DMSO-*d_6_*) δ 135.4, 135.3, 134.1, 134.0, 130.8, 130.7, 119.5, 118.6, 31.6, 30.4, 30.2, 28.8, 28.5, 22.5, 22.2, 22.2, 20.9, 20.4, 14.4. ESI-HRMS [M-I]^+^ m/z calcd for C_26_H_32_P^+^, 375.2236, found 375.2235. HPLC purity 99.2%, t_R_=20.204 min, C_8_ reversed-phase column (5 μm, 250×4.60 mm, Phenomenex), Water: MeCN from 100:0 to 0:100, 1.0 mL/min.

**TPP^+^-C_9_.** ^1^HNMR (DMSO-*d_6_*) δ 7.93–7.75 (m, 15 H), 3.68-3.54 (m, 2 H), 1.53–1.43 (m, 4 H), 1.27–1.21 (m, 10 H), 0.86 (t, 3 H, J=6.0 Hz). ^13^CNMR (DMSO-*d_6_*) δ 135.4, 135.3, 134.1, 134.0, 130.8, 130.7, 119.5, 118.6, 31.7, 30.4, 30.2, 29.1, 29.0, 28.6, 22.5, 22.2, 22.2, 20.9, 20.4, 14.4. ESI-HRMS [M-I]^+^ m/z calcd for C_27_H_34_P^+^, 389.2393, found 389.2395. HPLC purity 99.5%, t_R_=20.934 min, C_8_ reversed-phase column (5 μm, 250×4.60 mm, Phenomenex), Water: MeCN from 100:0 to 0:100, 1.0 mL/min.

**TPP^+^-C_10_.** ^1^HNMR (DMSO-*d_6_*) δ 7.93–7.75 (m, 15 H), 3.63-3.56 (m, 2 H), 1.53–1.44 (m, 4 H), 1.26–1.21 (m, 12 H), 0.86 (t, 3 H, J=6.4 Hz). ^13^CNMR (DMSO-*d_6_*) δ 135.4, 135.3, 134.1, 134.0, 130.8, 130.7, 119.5, 118.6, 31.7, 30.4, 30.2, 29.3, 29.2, 29.1, 28.6, 22.6, 22.2, 22.2, 20.9, 20.4, 14.4. ESI-HRMS [M-I]^+^ m/z calcd for C_28_H_36_P^+^, 403.2549, found 403.2548. HPLC purity 99.5%, t_R_=21.676 min, C_8_ reversed-phase column (5 μm, 250×4.60 mm, Phenomenex), Water: MeCN from 100:0 to 0:100, 1.0 mL/min.

**TPP^+^-C_11_.** ^1^HNMR (DMSO-*d_6_*) δ 7.93–7.75 (m, 15 H), 3.62–3.55 (m, 2 H), 1.53-1.42 (m, 4 H), 1.26–1.21 (m, 14 H), 0.86 (t, 3 H, J=6.4 Hz). ^13^CNMR (DMSO-*d_6_*) δ 135.4, 135.3, 134.1, 134.0, 130.8, 130.7, 119.5, 118.6, 31.7, 30.4, 30.2, 29.4, 29.4, 29.2, 29.2, 28.6, 22.6, 22.2, 22.2, 20.9, 20.4, 14.4. ESI-HRMS [M-I]^+^ m/z calcd for C_29_H_38_P^+^, 417.2706, found 147.2704. HPLC purity 99.9%, t_R_=22.330 min, C_8_ reversed-phase column (5 μm, 250×4.60 mm, Phenomenex), Water: MeCN from 100:0 to 0:100, 1.0 mL/min.

**TPP^+^-C_12_.** ^1^HNMR (DMSO-*d_6_*) δ 7.93–7.75 (m, 15 H), 3.62-3.55 (m, 2 H), 1.53–1.41 (m, 4 H), 1.26–1.22 (m, 16 H), 0.86 (t, 3 H, J=6.4 Hz). ^13^CNMR (DMSO-*d_6_*) δ 135.4, 135.3, 134.1, 134.0, 130.8, 130.7, 119.5, 118.6, 31.8, 30.4, 30.2, 29.5, 29.4, 29.2, 29.2, 28.6, 22.6, 22.2, 22.2, 20.9, 20.4, 14.4. ESI-HRMS [M-I]^+^ m/z calcd for C_30_H_40_P^+^, 431.2862, found 431.2860. HPLC purity 99.3%, t_R_=22.953 min, C_8_ reversed-phase column (5 μm, 250×4.60 mm, Phenomenex), Water: MeCN from 100:0 to 0:100, 1.0 mL/min.

**TPP^+^-C_13_.** ^1^HNMR (DMSO-*d_6_*) δ 7.92–7.77 (m, 15 H), 3.62–3.55 (m, 2 H), 1.53–1.45 (m, 4 H), 1.22 (m, 18 H), 0.86 (t, 3 H, J=6.4 Hz). ^13^CNMR (DMSO-*d_6_*) δ 135.4, 135.3, 134.1, 134.0, 130.8, 130.6, 119.5, 118.6, 31.8, 30.4, 30.2, 29.5, 29.5, 29.4, 29.4, 29.2, 28.6, 22.6, 22.2, 22.2, 20.9, 20.4, 14.4. ESI-HRMS [M-I]^+^ m/z calcd for C_31_H_42_P^+^, 445.3019, found 445.3016. HPLC purity 96.9%, t_R_=23.563 min, C_8_ reversed-phase column (5 μm, 250×4.60 mm, Phenomenex), Water: MeCN from 100:0 to 0:100, 1.0 mL/min.

**TPP^+^-C_14_.** ^1^HNMR (DMSO-*d_6_*) δ 7.93–7.75 (m, 15 H), 3.62–3.55 (m, 2 H), 1.53–1.45 (m, 4 H), 1.38–1.23 (m, 20 H), 0.86 (t, 3 H, J=6.4 Hz). ^13^CNMR (DMSO-*d_6_*) δ 135.4, 135.3, 134.1, 134.0, 130.8, 130.6, 119.5, 118.6, 31.8, 30.4, 30.2, 29.5, 29.5, 29.5, 29.4, 29.2, 28.6, 22.6, 22.2, 22.2, 20.9, 20.4, 14.4. ESI-HRMS [M-I]^+^ m/z calcd for C_32_H_44_P^+^, 459.3175, found 459.3173. HPLC purity 99.2%, t_R_=24.089 min, C_8_ reversed-phase column (5 μm, 250×4.60 mm, Phenomenex), Water: MeCN from 100:0 to 0:100, 1.0 mL/min.

**TPP^+^-C_16_.** ^1^HNMR (DMSO-*d_6_*) δ 7.91–7.79 (m, 15 H), 3.57–3.53 (m, 2 H), 1.51–1.45 (m, 4 H), 1.23 (m, 24 H), 0.87 (t, 3 H, J=6.0 Hz). ^13^CNMR (DMSO-*d_6_*) δ 135.4, 135.4, 134.1, 134.0, 130.8, 130.6, 119.5, 118.6, 31.8, 30.4, 30.2, 29.5, 29.5, 29.4, 29.4, 29.2, 28.6, 22.6, 22.2, 22.2, 20.9, 20.4, 14.4. ESI-HRMS [M-I]^+^ m/z calcd for C_34_H_48_P^+^, 487.3488, found 487.3486. HPLC purity 98.0%, t_R_=25.018 min, C_8_ reversed-phase column (5 μm, 250×4.60 mm, Phenomenex), Water: MeCN from 100:0 to 0:100, 1.0 mL/min.

**TPP^+^-C_18_.** ^1^HNMR (DMSO-*d_6_*) δ 7.93–7.75 (m, 15 H), 3.61–3.54 (m, 2 H), 1.53–1.43 (m, 4 H), 1.26–1.23 (m, 28 H), 0.86 (t, 3 H, J=6.4 Hz). ^13^CNMR (DMSO-*d_6_*) δ 135.4, 135.3, 134.1, 134.0, 130.8, 130.6, 119.5, 118.6, 31.8, 30.4, 30.2, 29.5, 29.5, 29.5, 29.4, 29.2, 28.6, 22.6, 22.2, 22.2, 20.9, 20.4, 14.4. ESI-HRMS [M-I]^+^ m/z calcd for C_36_H_52_P^+^, 515.3801, found 515.3799. HPLC purity 98.3%, t_R_=25.758 min, C_8_ reversed-phase column (5 μm, 250×4.60 mm, Phenomenex), Water: MeCN from 100:0 to 0:100, 1.0 mL/min.

**Scheme S2**. Synthesis route of TPP^+^-C_14_-NBD.

**Synthesis of** **1,14-diiodotetradecane.** Imidazole (2.0 g, 30 mmol), triphenylphosphine (7.9 g, 30 mmol), and Iodine (7.6 g, 30 mmol) were added into the THF (50 mL) solution of 1,14-tetradecanediol (2.3 g, 10 mmol) at -10 °C, which was allowed to be stirred for 1 h at 0 °C and then for 12 h at room temperature. The mixture was purified using a silica column with petroleum ether (PE) as the eluent to afford the white solid (4.2 g). Yield 93.3%. ^1^HNMR (CDCl_3_) δ 3.17 (t, 4 H, J=7.2 Hz), 1.86–1.78 (m, 4 H), 1.43-1.26 (m, 20 H).

**Synthesis of** **PhtN-C_14_-I.** To the suspension of phthalimide potassium salt (0.56 g, 3 mmol) in 60 mL DMF was added 1,14-diiodotetradecane (4.05 g, 9 mmol). The mixture was then stirred for 24 h at room temperature. The reaction solution was diluted with 100 mL of ethyl acetate and then washed with water and brine, dried with anhydrous sodium sulfate overnight. The organic solution was collected and concentrated under vacuum. The residue was purified using a silica column with PE/EA as the eluent to afford the white solid (1.1 g). Yield 78.6%. ^1^HNMR (CDCl_3_) δ 7.85–7.83 (m, 2 H), 7.72–7.70 (m, 2 H), 3.66 (t, 2 H, J=7.2 Hz), 3.17 (t, 2 H, J=6.8 Hz), 1.85–1.78 (m, 2 H), 1.69–1.65 (m, 2 H), 1.40–1.25 (m, 20 H).

**Synthesis of TPP^+^-C_14_-NPht****.** The solution of triphenylphosphine (525 mg, 2 mmol) and PhtN-C_14_-I (0.94 g, 2 mmol) in MeCN (30 mL) was refluxed for 72 h. The solvent was removed under vacuum, and the residue was purified using a silica column with EA/MeOH as the eluent to afford the light yellow solid (0.88 g). Yield 60%. ^1^HNMR (CDCl_3_) δ 7.86–7.69 (m, 19 H), 3.72–3.65 (m, 4H), 1.66–1.62 (m, 6H), 1.31–1.18 (m, 6H). ESI-MS [M-I]^+^ m/z calcd for C_40_H_47_NO_2_P^+^, 604.33, found 604.87.

**Synthesis of TPP^+^-C_14_-NH_2_.** To the ethanol solution (50 mL) of TPP^+^-C_14_-NPht (1.1 g, 1.5 mmol) was added hydrazine monohydrate (221 μL, 4.5 mmol). The mixture was refluxed at 85 °C for 4 h. The solution was cooled to room temperature, resulting in the precipitation of a substantial amount of white solid. After removing the solid by filtration, the filtrate was concentrated to afford the yellow oil (500 mg). Yield 66.7%. ^1^HNMR (CD_3_OD) δ 7.93–7. 52 (m, 15 H), 3.47–3.40 (m, 2H), 2.76 (t, 2H, J=7.2 Hz), 1.71–1. 54 (m, 6H), 1.34–1.28 (m, 18H). ESI-MS [M-I]^+^ m/z calcd for C_32_H_45_NP^+^, 474.33, found 474.53.

**Synthesis of** **TPP^+^-C_14_-NBD.** To the solution of TPP^+^-C_14_-NH_2_ (118.6 mg, 0.25 mmol) in anhydrous ethanol (10 mL) was added triethylamine (105 μL, 0.75 mmol) and 4-chloro-7-nitrobenzofurazan (50 mg, 0.25 mmol), which was stirred at room temperature for 12 h, followed by dilution with 75 mL of dichloromethane. The organic phase was washed with water and brine, respectively. After drying with anhydrous sodium sulfate, the mixture was purified using a silica column with EA/MeOH as the eluent to afford the yellow solid (85 mg). Yield 43%. ^1^HNMR (DMSO-*d_6_*) δ 9.57 (s, 1H), 8.52 (d, 1H, J=8.8 Hz), 7.90–7.78 (m, 15 H), 6.42 (d, 1H, J=8.4 Hz), 3.46–3.55 (m, 4H), 1.67–1.18 (m, 24H). ESI-MS [M-I]^+^ m/z calcd for C_38_H_46_N_4_O_3_P^+^, 637.78, found 637.75.

**Cell viability.** Cells were seeded in 96-well plates at 2,000 cells per well and incubated overnight at 37 °C. After treatment with dilutions of the indicated compounds for 72 h, cell viability was assessed using the CellTiter-Glo kit (Promega) according to the manufacturer’s instructions. Luminescence was measured with a microplate reader (Cytation 5, Bio-Tek).

**Colony formation.** Tumor cells were seeded in 6-well plates at 500 cells per well and incubated overnight, followed by the treatment with the indicated compounds (100 nM) for ~14 days. During this period, the culture medium was replaced regularly. Colonies were stained with crystal violet and recorded.

**Cell growth** **assay.** Cells were grown in 6-well plates and were treated as indicated. The cell number in each well was counted every 24 h by using an automatic cell counter (iCytal, Jimbio).

**Apoptosis assay.** Cells were treated with indicated compounds for 48 h, followed by harvesting and staining with Annexin V-FITC and propidium iodide (Beyotime, #C1062M) for 15 min. The apoptotic population was analyzed by a flow cytometer (CytoFLEX, Beckman Coulter).

**Caspase 3/7 activity.** Caspase 3/7 activity was assessed using a fluorogenic substrate-based assay. Tumor cells (10,000 cells/well) were seeded into black, flat-bottom 96-well plates and cultured overnight under standard conditions (37 °C, 5% CO_2_, complete growth medium). Cells were then treated for 48 h with Camptothecin (CPT, 10 μM, positive control), TPP^+^-C_14_ (200 nM or 500 nM), or their combinations with Z-VAD-FMK (20 μM, pan-caspase inhibitor) or Ac-DEVD-CHO (20 μM, caspase-3 inhibitor). Untreated cells served as the negative control. Following treatment, the culture medium was removed and replaced with PBS containing 5 μM Caspase 3/7 substrate (Beyotime, #C1168M). Plates were incubated in the dark at room temperature for 30 min to allow substrate cleavage. Fluorescence intensity was measured at an excitation/emission wavelength of 485/515 nm using a microplate reader (Cytation 5, BioTek). Data were normalized to cell viability, and all experiments were performed in triplicate.

**Puncta formation assay of** **mCherry-GFP-LC3B.** Tumor cells were transfected with mCherry-GFP-LC3B adenovirus (Beyotime, #C3011) according to the manufacturer's instructions. After being treated as indicated, the cells were observed through confocal microscopy (LSM800, Zeiss).

**Cellular ROS measurement.** The detection was performed by using the reactive oxygen species assay kit (Beyotime, #S1105) according to the manufacturer's instructions. Briefly, tumor cells were incubated with 500 nM TPP^+^-C_14_ for 24 h. The cells were then harvested and incubated with 10 µM DCFH-DA at 37°C for 30 min, followed by analysis using a flow cytometer (Cytoflex, Beckman Coulter).

**Mitochondrial ROS measurement.** Tumor cells were grown in a confocal dish, and then treated with 500 nM TPP^+^-C_14_ for 24 h, followed by a 30-minute incubation with MitoROS 580 (AAT Bioquest, #16052) at 37°C in the dark. After staining, cells were washed three times with HHBS. Imaging was performed using a confocal microscope (FV4000, Olympus).

**Cellular Calcium imaging.** Tumor cells were seeded in a confocal plate and incubated overnight to ensure optimal adhesion. After incubation with TPP^+^-C_14_ for 24 h and Fluo-4 AM (GlpBio, #GC30231) for 30 min, respectively, cells were observed using a confocal microscope (FV4000, Olympus).

**Mitochondrial membrane potential measurement using JC-1.** According to the manufacturer's protocol, the cells treated as indicated were incubated with JC-1 (Beyotime, #C2006) for 30 min and were analyzed by confocal microscopy (LSM800, Zeiss) and flow cytometer (Cytoflex, Beckman Coulter), respectively.

**Relative quantitation of ATP*.*** Tumor cells were seeded at a density of 15,0000 cells per well in 6-well plates and were subjected to the indicated treatments. After treatment, the cells were harvested, and 10,000 cells per group were analyzed for ATP detection using the CellTiter-Glo Luminescent Assay (Promega, #G7572). Luminescence was measured with a microplate reader (Cytation 5, BioTek). Data was normalized to the untreated group.

**Competitive Binding with NAO in PBS solution.** This assay was performed in black 96-well plates with a total volume of 100 μL per well. Cardiolipin (CL), NAO, and the tested compounds (TPP^+^-C_n,_ n-tetradecane, or 1-tetradecanol) were diluted to the specified concentration in cold PBS (10 mM, pH 7.4) and stored on ice before use. Three experimental groups were established: NAO (20 μM), a mixture of NAO (20 μM) and CL (20 μM), and a mixture of NAO (20 μM), CL (20 μM), and the test compound (20 μM). For the co-incubation group, NAO and CL were added to the wells first, followed by the tested compound. Fluorescence intensity was measured using a microplate reader (Cytation 5, BioTek) with excitation/emission wavelengths of 499/530 nm.

**Competitive cell imaging with NAO.** Tumor cells were seeded in confocal dishes and allowed to adhere overnight at 37°C. The cells were treated with NAO alone or co-incubated with NAO and TPP^+^-C_1_ or TPP^+^-C_14_ together. The co-incubation group was subjected to two sequential treatment protocols: incubation with 500 nM NAO for 30 min, followed by 1 μM TPP^+^-C_1_ or TPP^+^-C_14_ for an additional 30 min, or pretreated with 1 μM TPP^+^-C_1_ or TPP^+^-C_14_ for 30 min, followed by 500 nM NAO for another 30 min. After treatment, fluorescence images were captured using a confocal laser scanning microscope (LSM800, Zeiss)

**TEM imaging of cells.** Tumor cells were treated as indicated and were fixed in pre-warmed electron microscopy fixative at room temperature for 5 min in the dark. Cells were then gently detached using a cell scraper and transferred to centrifuge tubes. After low-speed centrifugation (1500 rpm, 3 min), the resulting pellet was resuspended in fresh fixative and incubated at 4 °C for 24 h. Samples were processed and imaged by TEM (HT7800, Hitachi ) in Servicebio Technology (Wuhan, China).

**Immunohistochemical (IHC) analysis.** Tumor tissues from the *in vivo* treatment study were harvested and fixed in 4% paraformaldehyde (PFA) at a tissue-to-fixative ratio of 1:7 for 48 h. Paraffin-embedded sections were sent to Servicebio Technology (Wuhan, China) for IHC staining. The percentage of positively stained cells was quantitatively analyzed using the ImageJ software.

**Hematoxylin and eosin (H&E) staining.** Major organs for the toxicity study or tumor tissues from the *in vivo* treatment study were harvested from ICR mice and fixed in 4% paraformaldehyde (1:7, w/v) for 48 h, following the same protocol as for immunohistochemistry. Hematoxylin and eosin (H&E) staining was subsequently performed by Servicebio Technology (Wuhan, China).

**Hematological and comprehensive metabolic panel analysis****.** Whole blood samples were obtained through the eyeball and collected into blood collection tubes. Subsequently, a mini 5-part hematology analyzer (Dymond) was employed to assess the blood parameters. The whole blood samples were centrifuged to separate the serum, which was analyzed using a comprehensive metabolic panel.


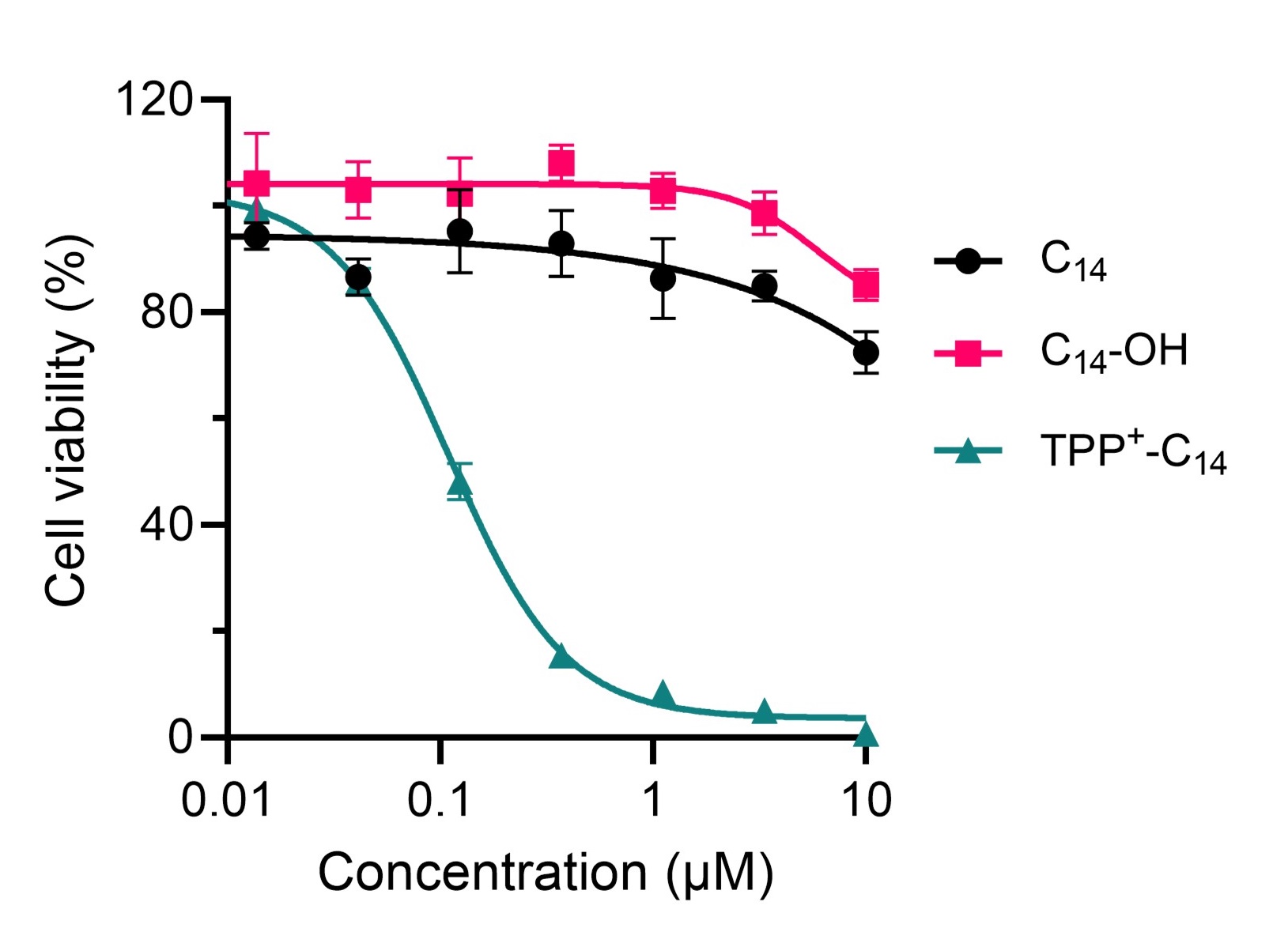


**Figure S1**. Viability curves of HeLa cells treated with various concentrations of TPP^+^-C_14_, C_14_ (n-tetradecane), or C_14_-OH (1-tetradecanol) for 72 h.


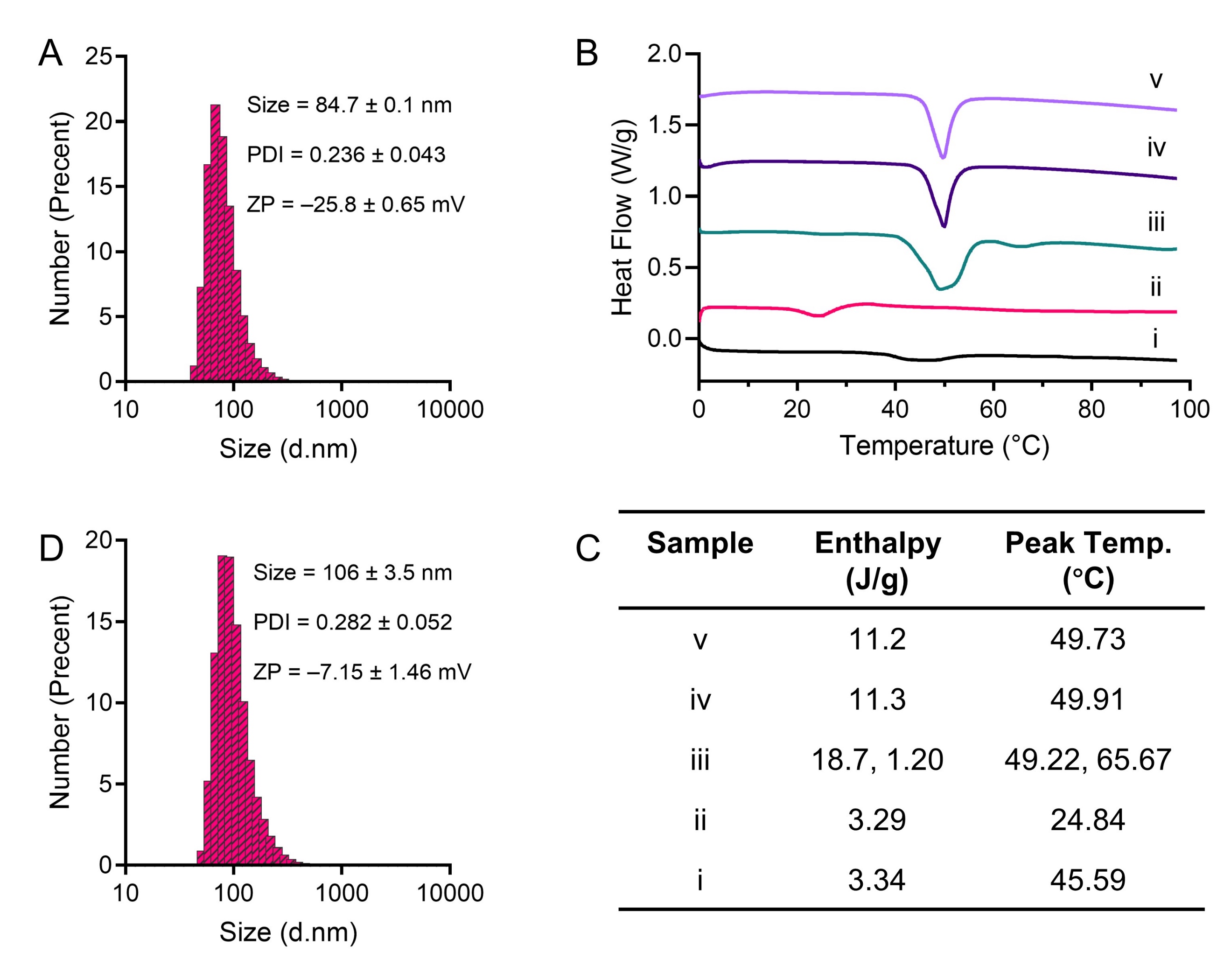


**Figure S2**. A) Size distribution and zeta potential of blank liposomes. B) Differential scanning calorimetry (DSC) chromatogram of (i) lecithin, (ii) cholesterol, (iii) physical mixture of lecithin, cholesterol, DSPE-mPEG 2000, and TPP^+^-C_14_, (iv) blank liposome, and (v) liposomal TPP^+^-C_14_. C) Summary of melting enthalpy and peak temperature values determined from the DSC curves. D) Size distribution and zeta potential of DiD-labeled liposomal TPP^+^-C_14_, formulated 0.5 mg/mL TPP^+^-C_14_ and 0.05 mg/mL DiD.


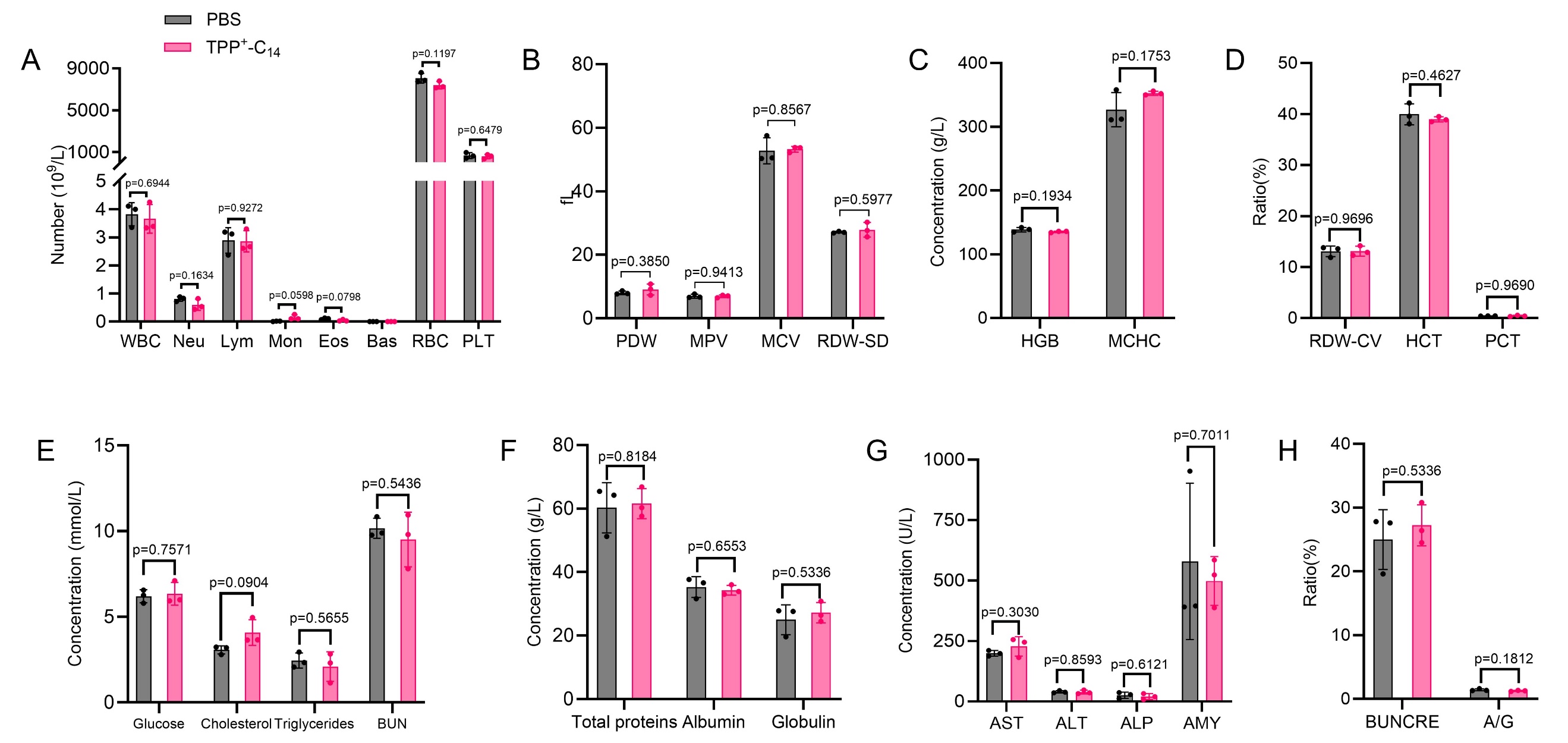


**Figure S3**. Hematological parameters (A−D) and the comprehensive metabolic panel (E−H) of blood samples from ICR mice treated with vehicle or TPP^+^-C_14_ (10 mg/kg). Data represent mean ± SD (n = 3). Statistical significance was determined using a two-tailed Student’s t-test.


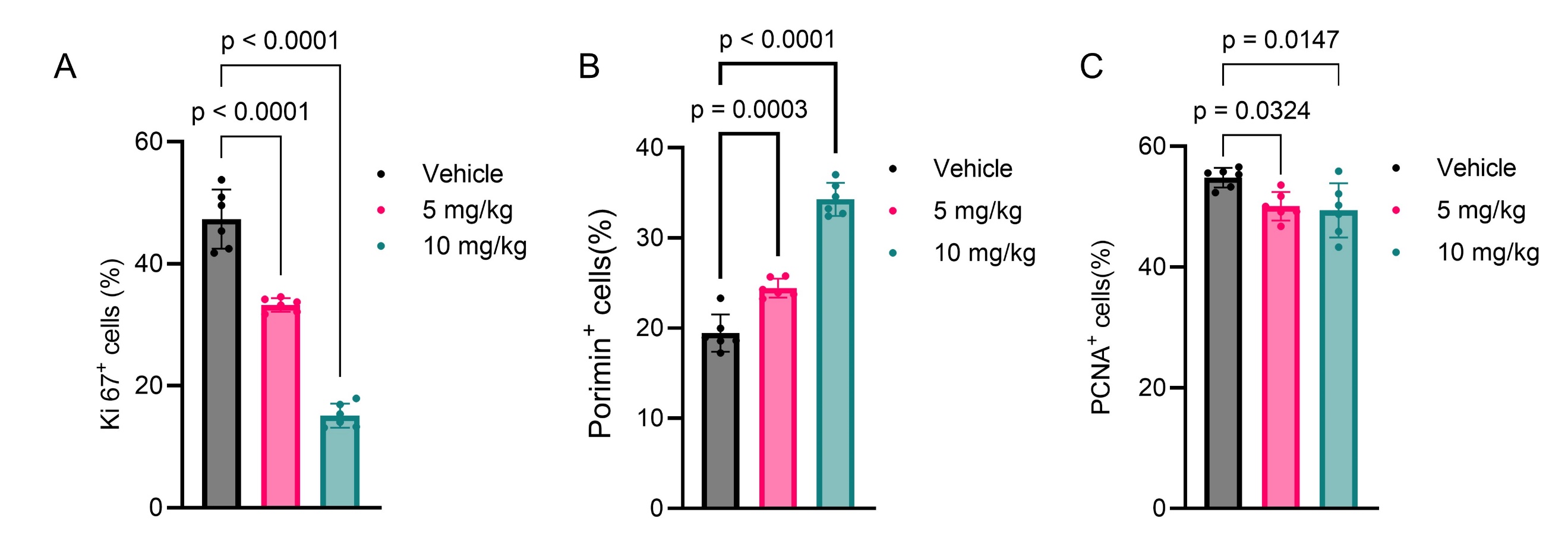


**Figure S4.** Quantitative immunohistochemistry (IHC) analysis was performed to determine the percentages of positive cells using ImageJ software. The analysis included: A) Ki 67. B) Porimin. C) PCNA. Data are presented as mean ± SD (n = 6). Statistical significance was determined using one-way ANOVA by Dunnett's multiple comparisons test.


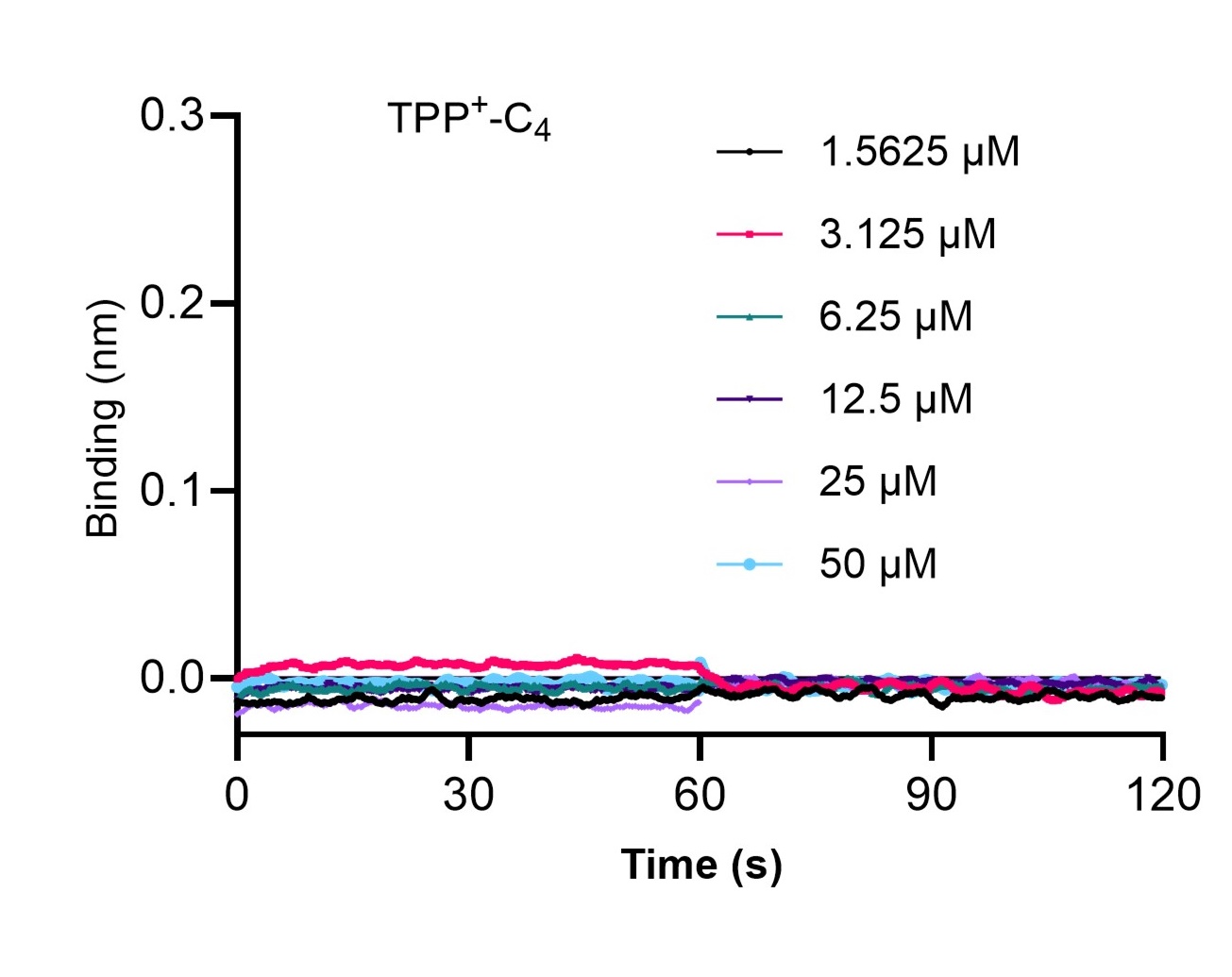


**Figure S5.** BLI binding kinetics of TPP^+^-C_4_ with cardiolipin.


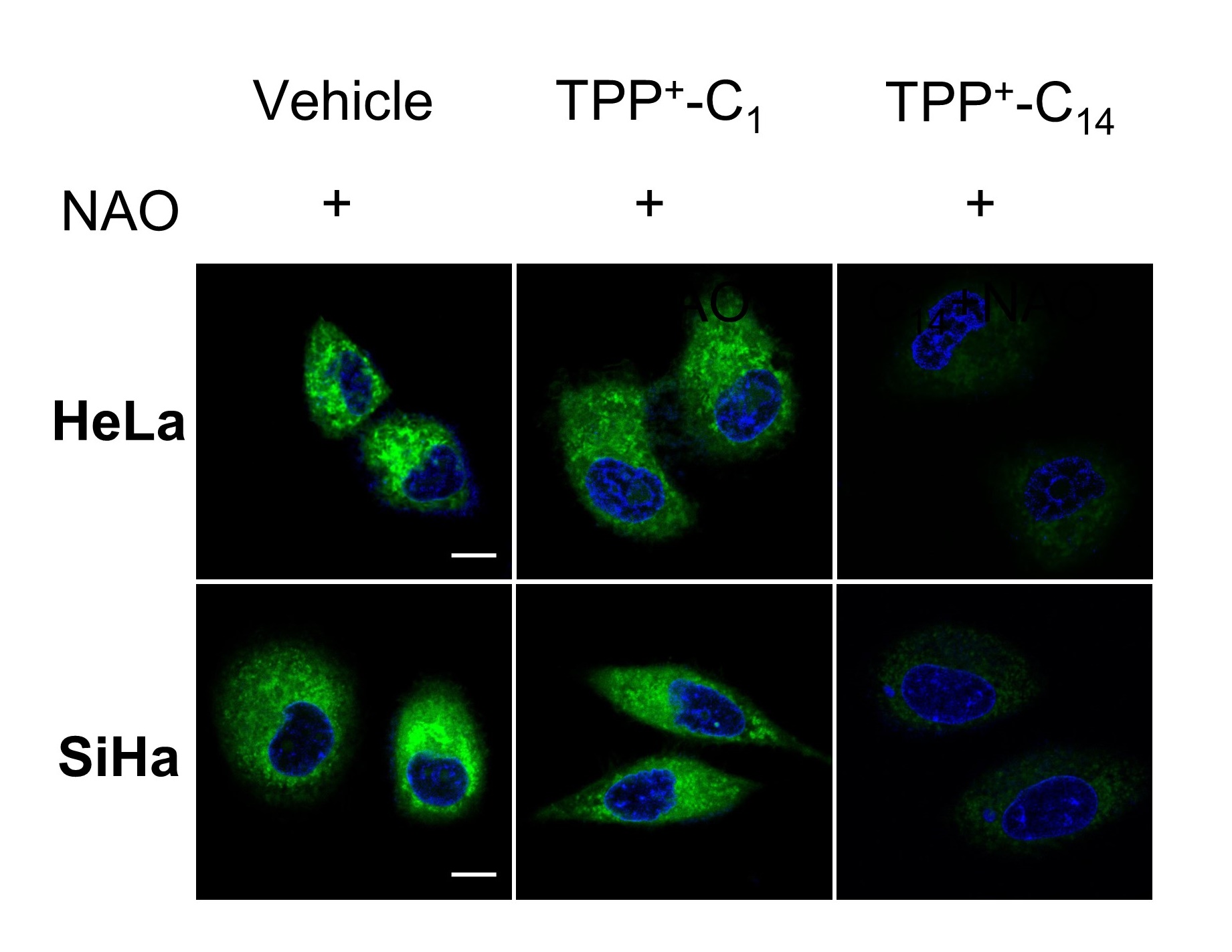


**Figure S6.** Representative images showing competitive binding to cardiolipin of NAO and TPP^+^-C_1_ or TPP^+^-C_14_. Cells were pretreated with TPP^+^-C_1_ or TPP^+^-C_14_ (500 nM, 30 min) followed by staining with NAO (500 nM, 30 min). Scale bar: 10 μm.


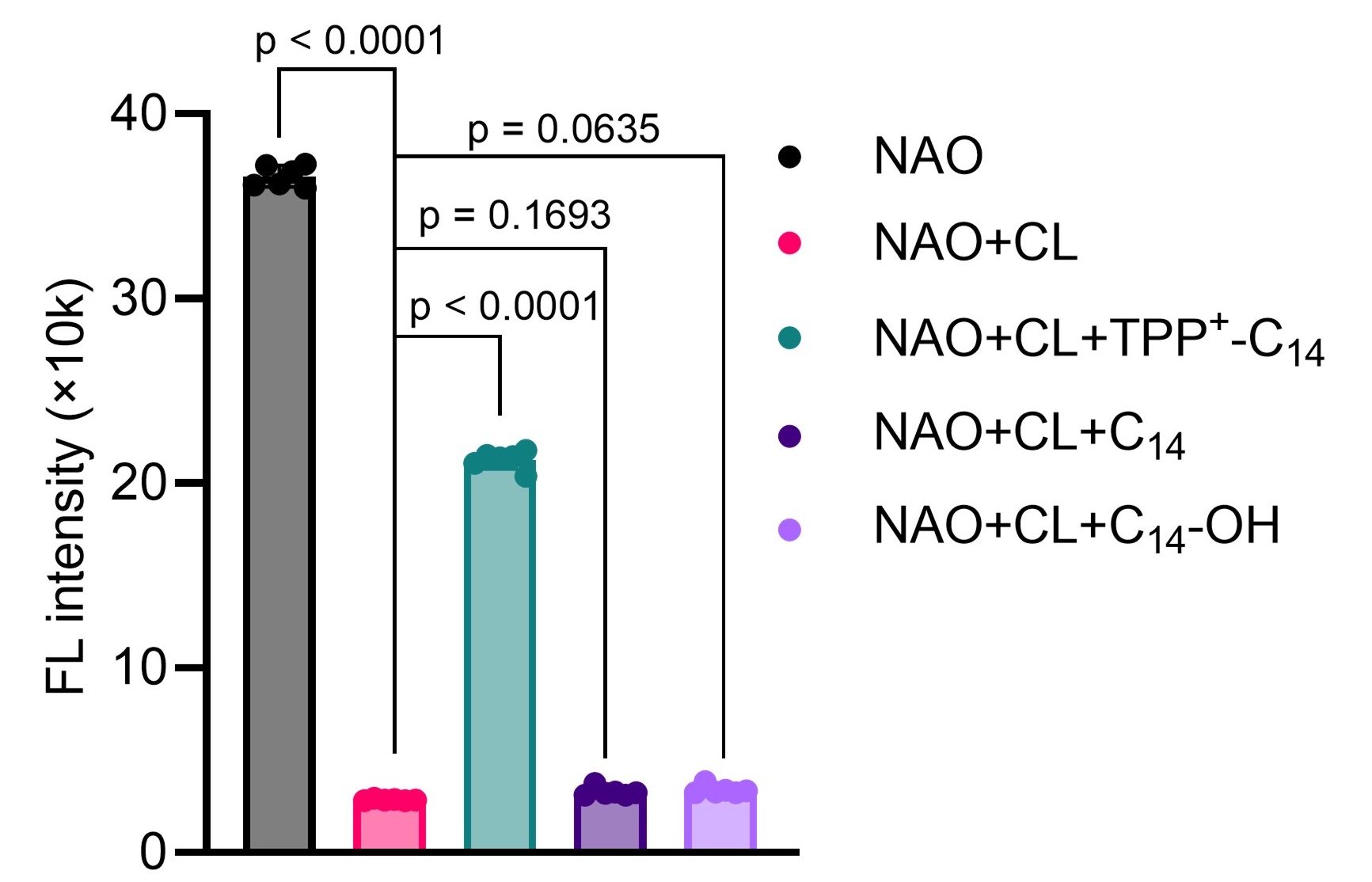


**Figure S7.** Competitive binding assay to cardiolipin (CL) in PBS. NAO (20 μM) was incubated with CL (20 μM) before exposure to 20 μM of C_14_ (n-tetradecane), C_14_-OH (1-tetradecanol), or TPP^+^-C_14_. Fluorescence intensity was measured at an excitation/emission wavelength of 499/530 nm. Data represent mean ± SD (n = 6). Statistical significance was determined using one-way ANOVA by Dunnett’s multiple comparisons test.


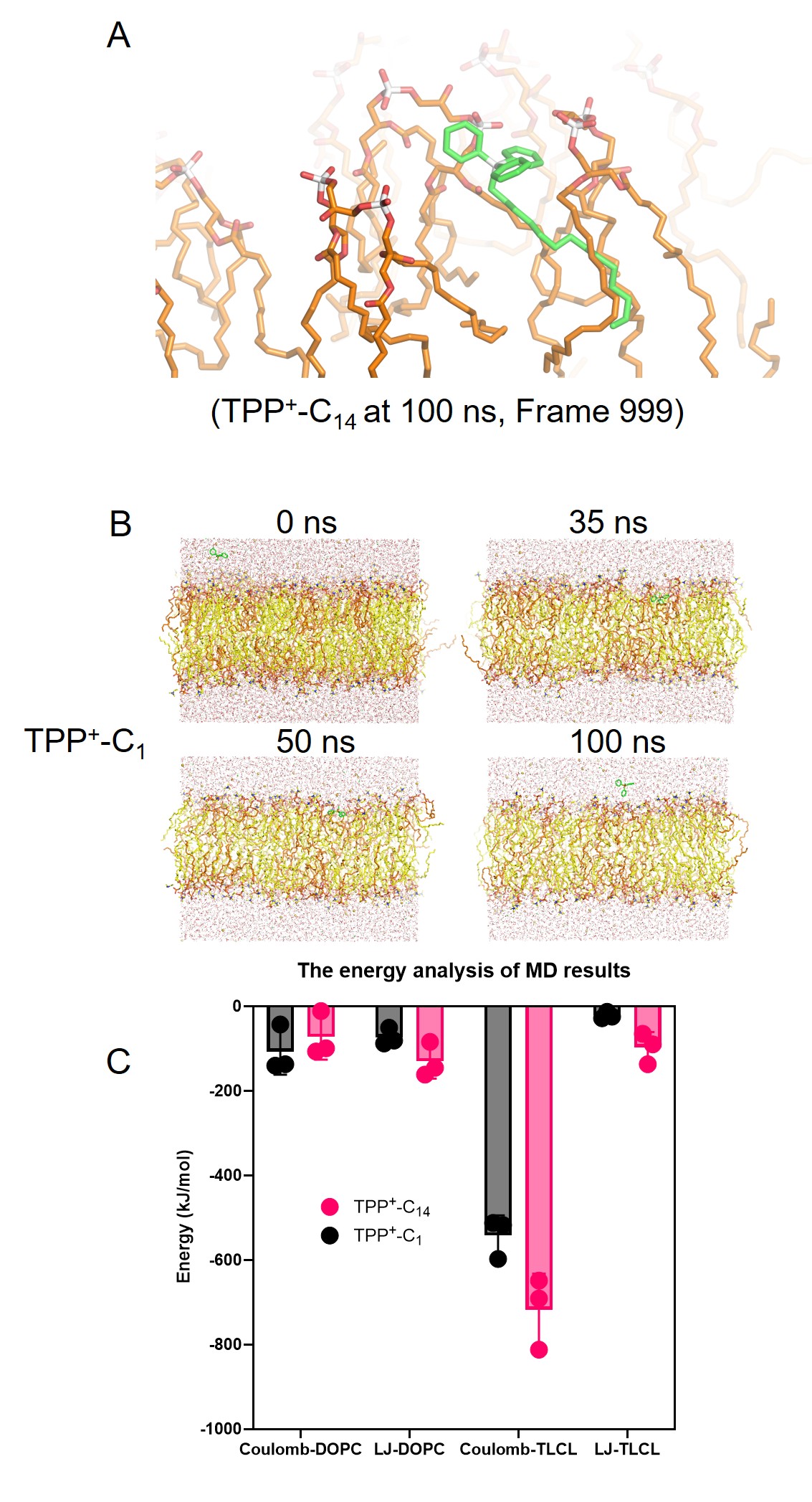


**Figure S8.** A, B) MD simulations showing the interactions of cardiolipin and TPP^+^-C_14._ A) or TPP^+^-C_1._ B) DOPC: Yellow, TLCL (-2*e* charge): Brown, TPP^+^-C_14_ or TPP^+^-C_1_: Green. C) Coulombic energy analysis of the corresponding interaction of TPP^+^-C_14_ or TPP^+^-C_1_ with DOPC and TLCL. Data are shown as mean ± SD（n=3). Statistical significance was determined using a two-tailed Student’s t-test.


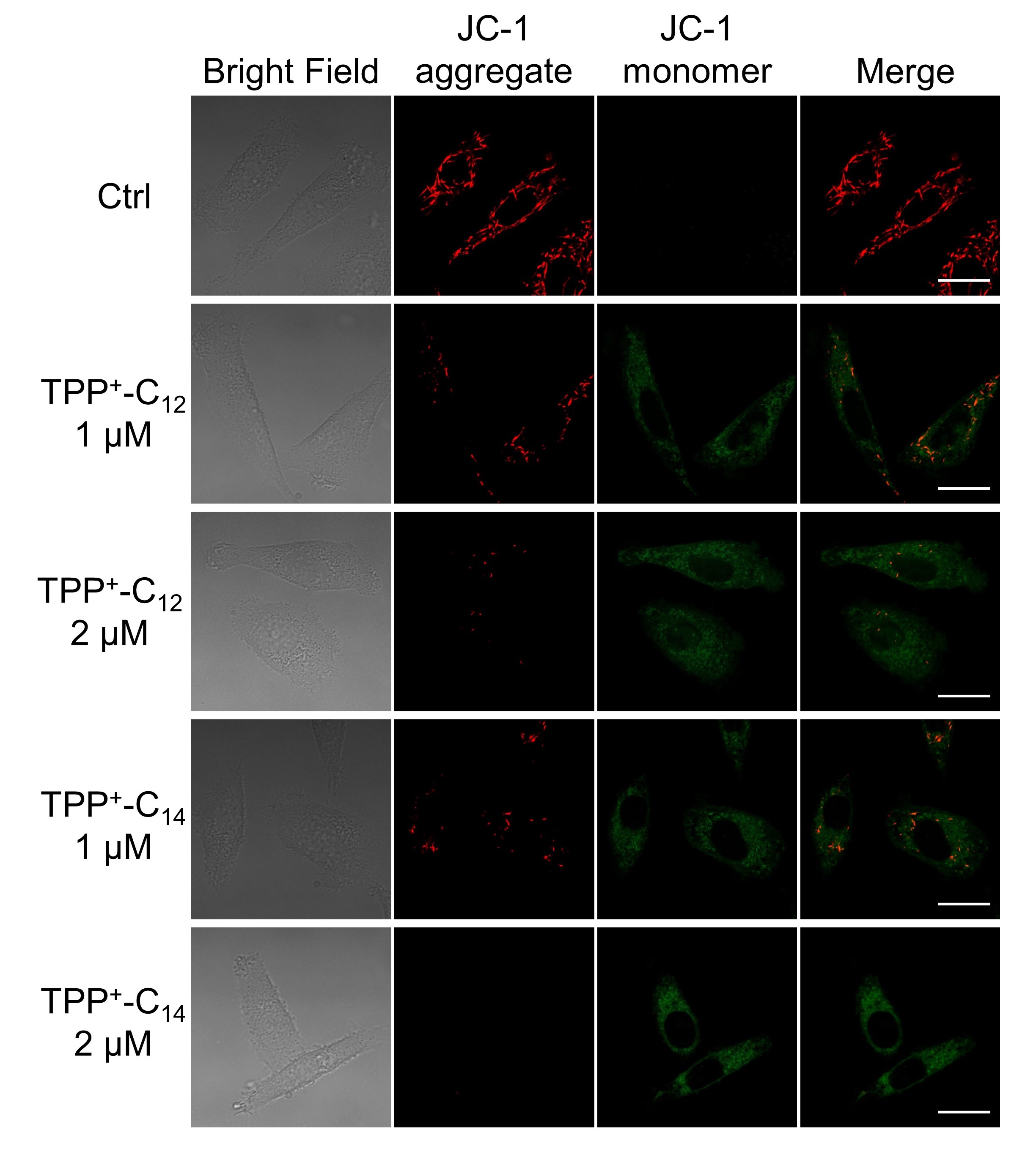


**Figure S9**. JC-1 imaging of SiHa cells that were treated with TPP^+^-C_12_ or TPP^+^-C_14_ for 6 h, and subsequently stained with JC-1 for 30 min. Scale bar: 20 μm.


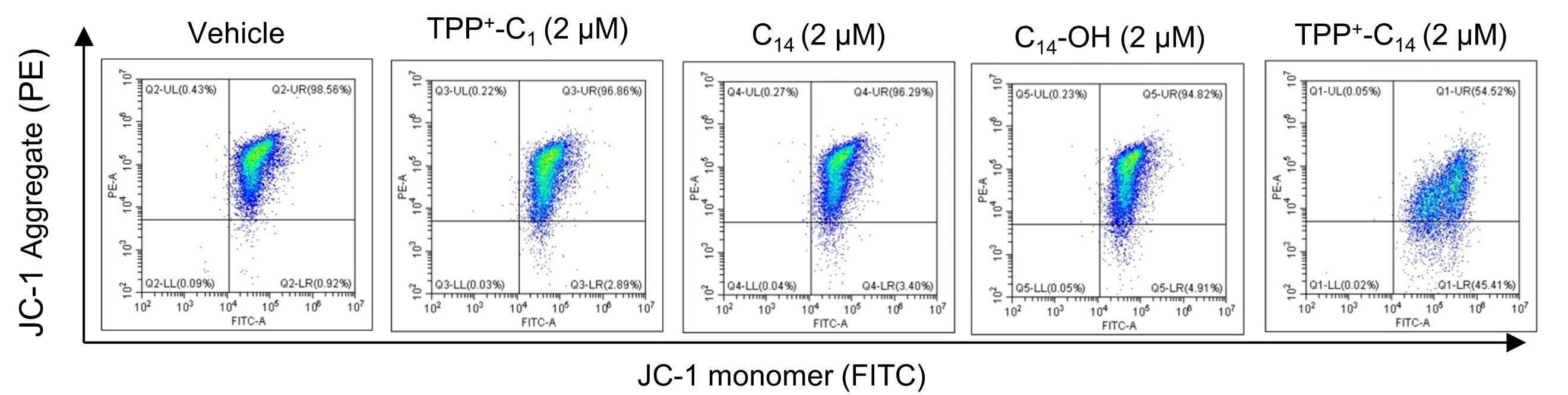


**Figure S10.** Flow cytometric quantification of JC-1 staining. HeLa cells were treated with the indicated concentration of C_14_ (n-tetradecane), C_14_-OH (1-tetradecanol), or TPP^+^-C_14_ for 6 h, harvested, and stained with JC-1.


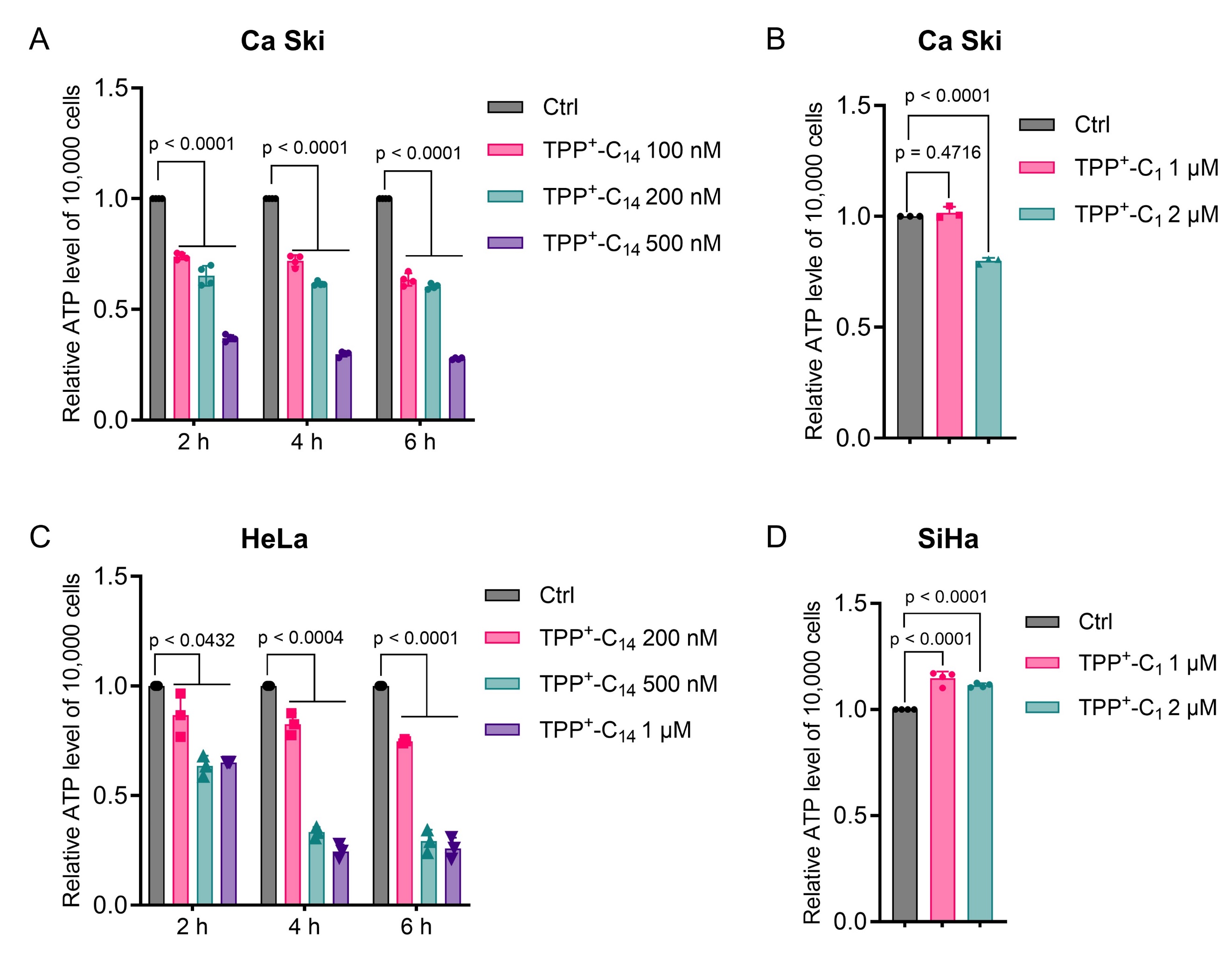


**Figure S11.** Relative quantification of ATP levels in cervical cancer cells that were treated with TPP^+^-C_14_ or TPP^+^-C_1_ for the indicated durations. The treatment time with TPP^+^-C_1_ is 6 h for B and D. Data are shown as mean ± SD (n=3). Statistical significance was determined using one-way ANOVA by Dunnett’s multiple comparisons test.


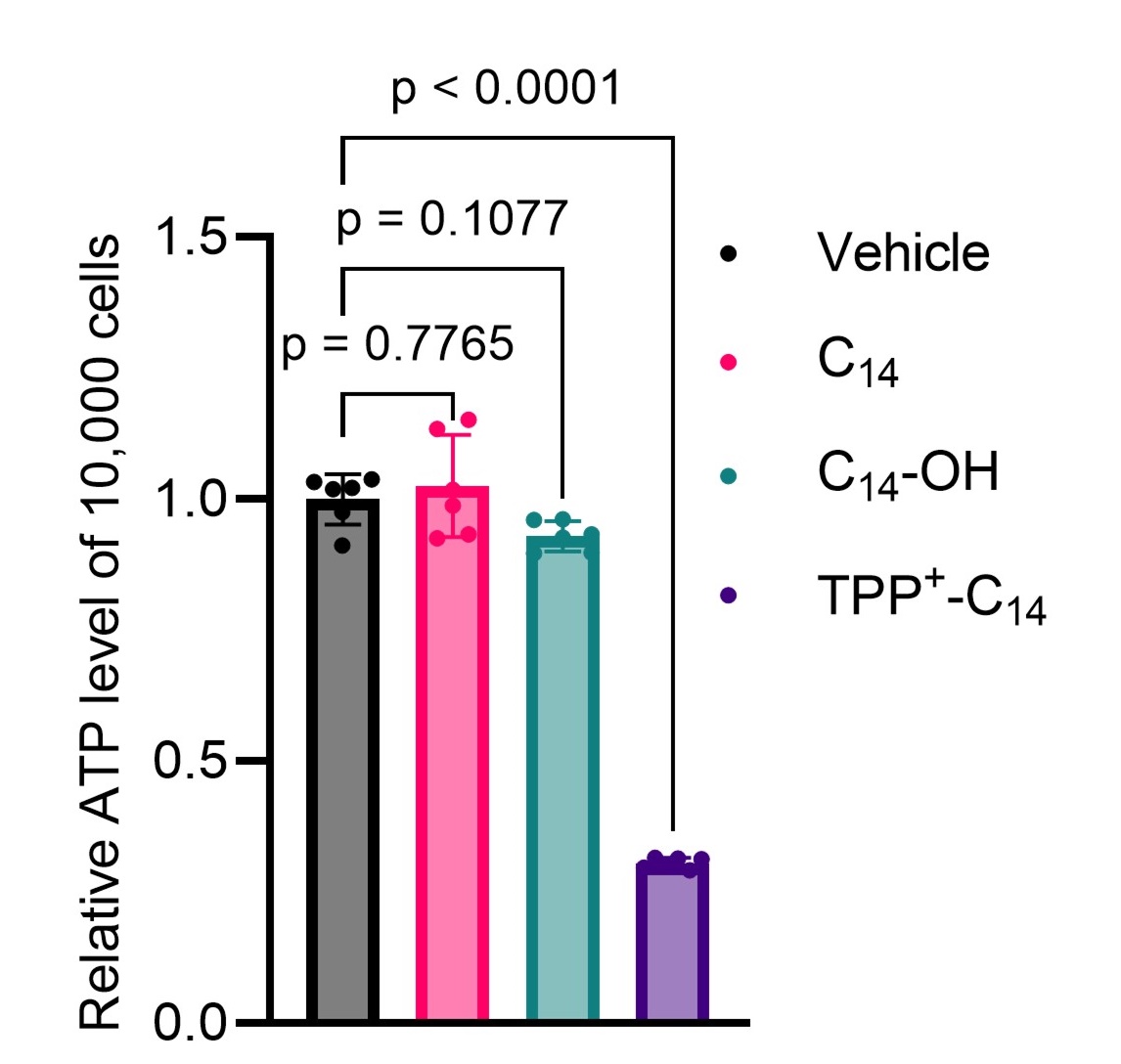


**Figure S12.** Relative quantification of cellular ATP levels. HeLa cells were treated with 1 μM of C_14_ (n-tetradecane), C_14_-OH (1-tetradecanol), or TPP^+^-C_14_ for 6 h. Subsequently, 10000 cells per group were collected for ATP quantification. Data are displayed as mean ± SD (n = 6). Statistical significance was determined using one-way ANOVA by Dunnett’s multiple comparisons test.


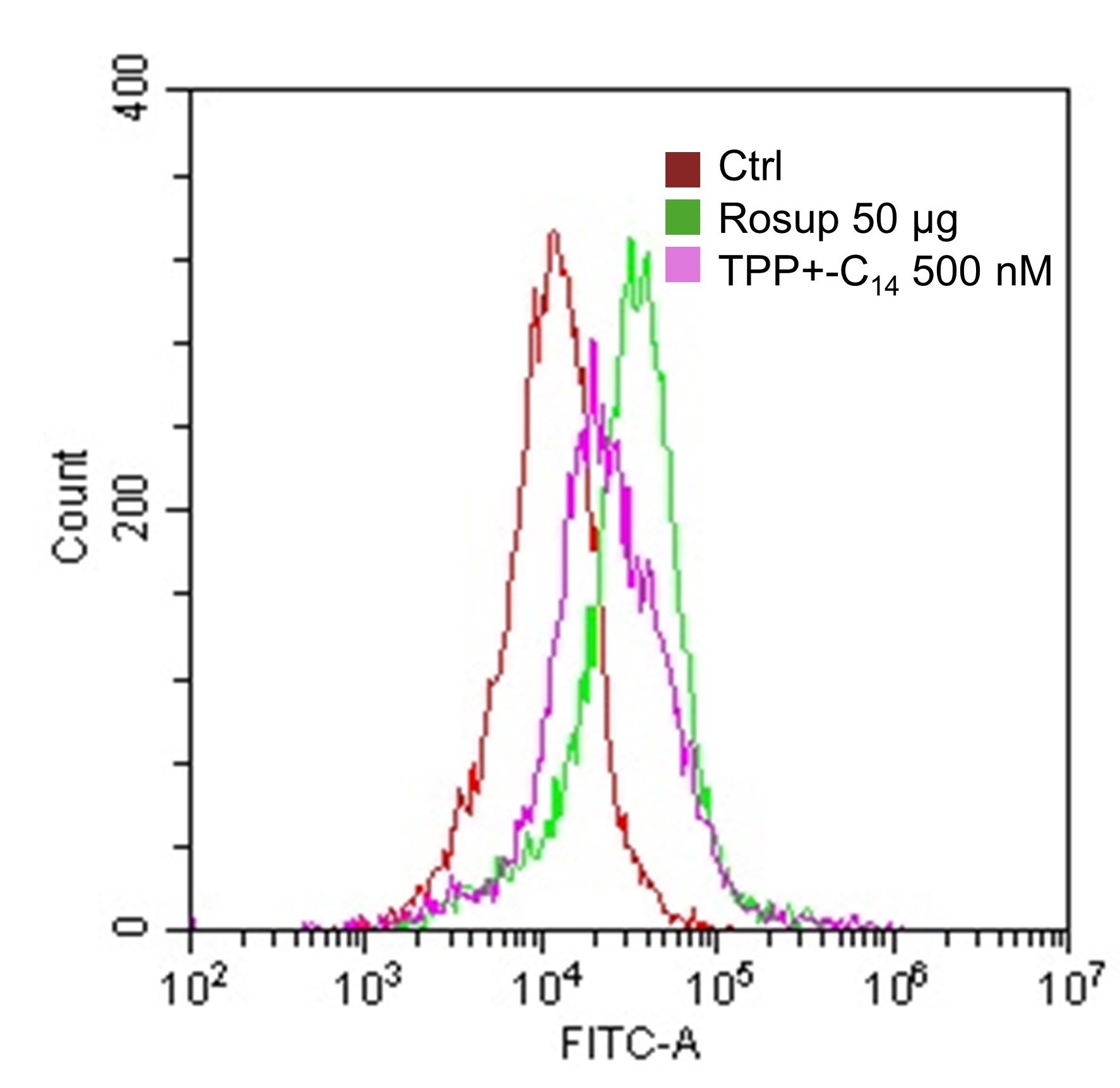


**Figure S13.** Flow cytometric analysis of ROS in HeLa cells that were treated with TPP^+^-C_14_ (500 nM) for 24 h or Rosup® (50 μg/mL, positive control), followed by incubation with DCFH-DA (5 μM, 30 min).


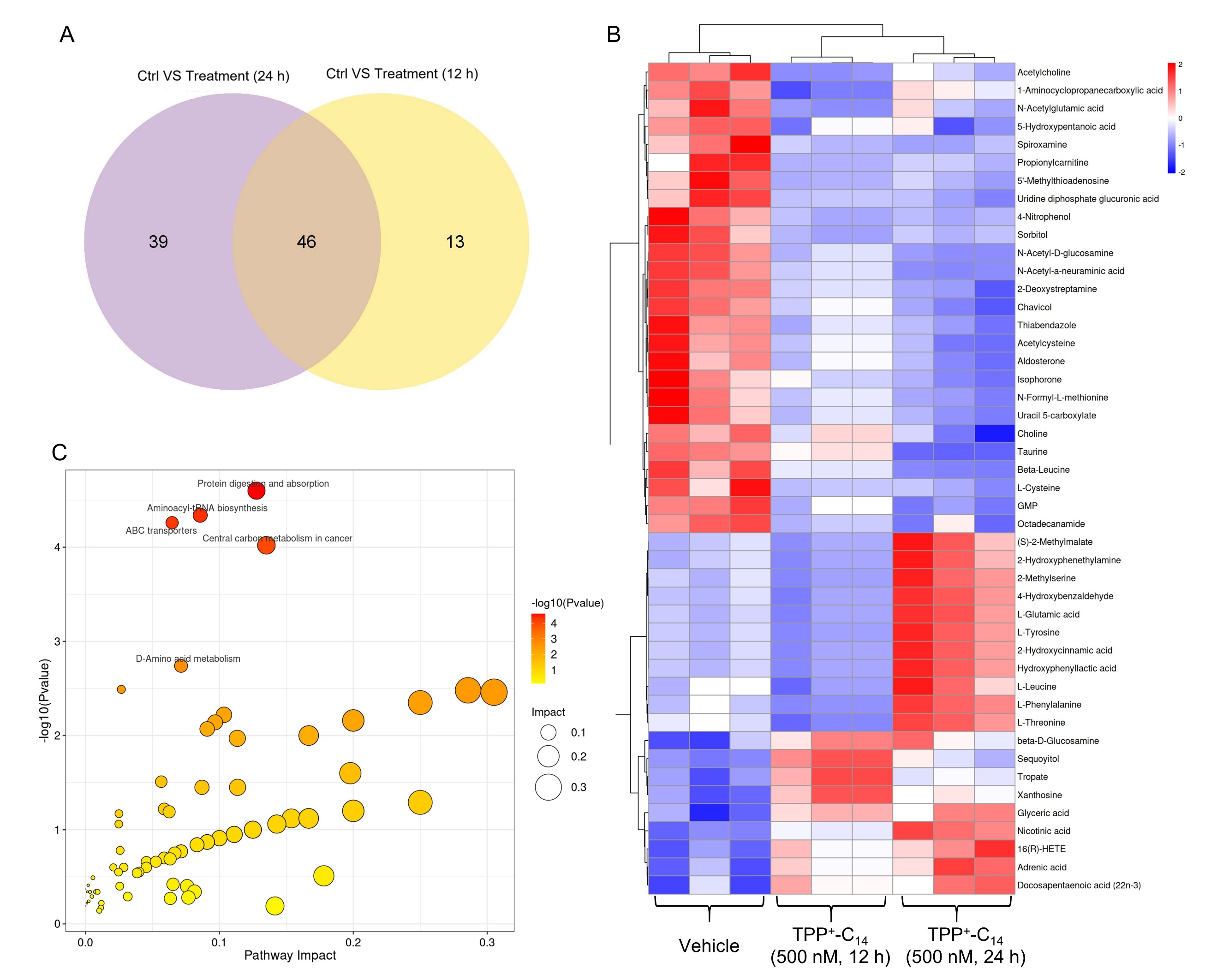


**Figure S14.** Metabolic reprogramming of HeLa cells that were treated with Vehicle, 500 nM TPP^+^-C_14_ for 12 h**,** or 500 nM TPP^+^-C_14_ for 24 h, respectively. A) Venn diagram depicting the differential expression of metabolites across the treatment groups. B) Identification of co-differentially expressed metabolites between treatment conditions. C) KEGG pathway enrichment analysis of co-differentially expressed metabolites.


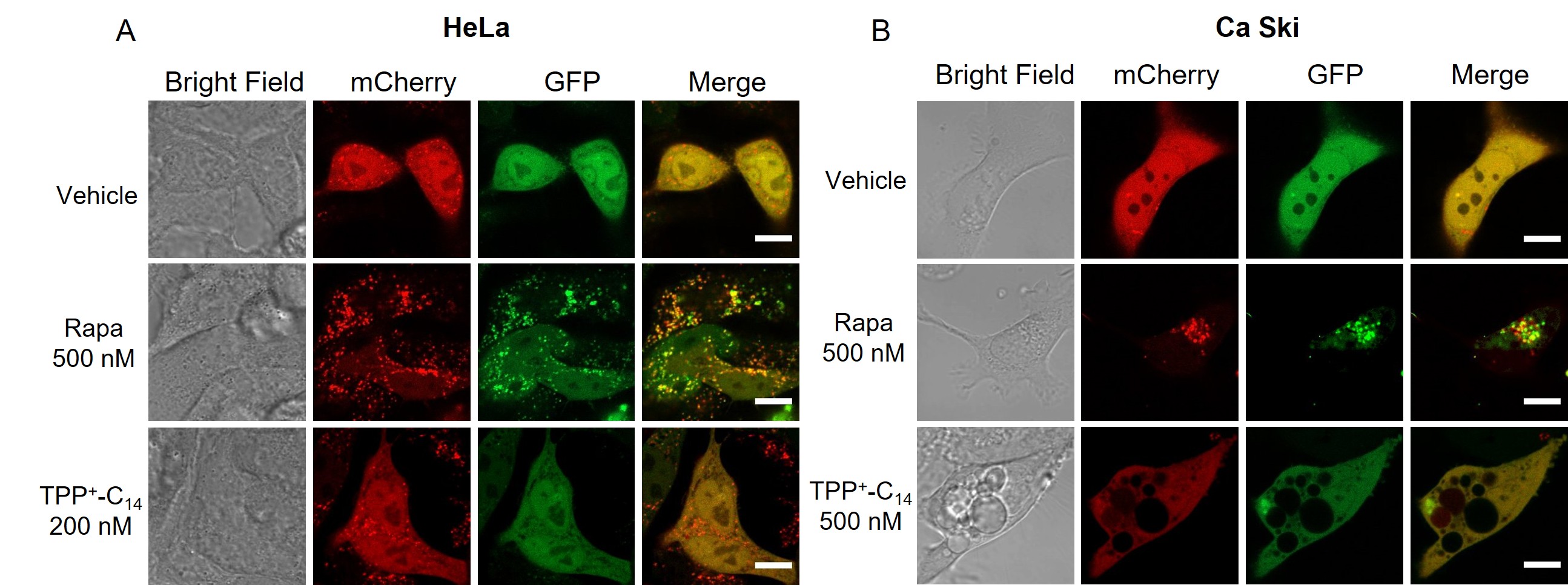


**Figure S15.** Confocal imaging of mCherry-GFP-LC3B-transfected A) HeLa cells and B) Ca Ski cells. The transfected cells were treated with the indicated concentration of Rapamycin or TPP^+^-C_14_ for 24 h. Autolysosomes: red puncta, Autophagosomes: yellow puncta. Scale bar: 10 μm.


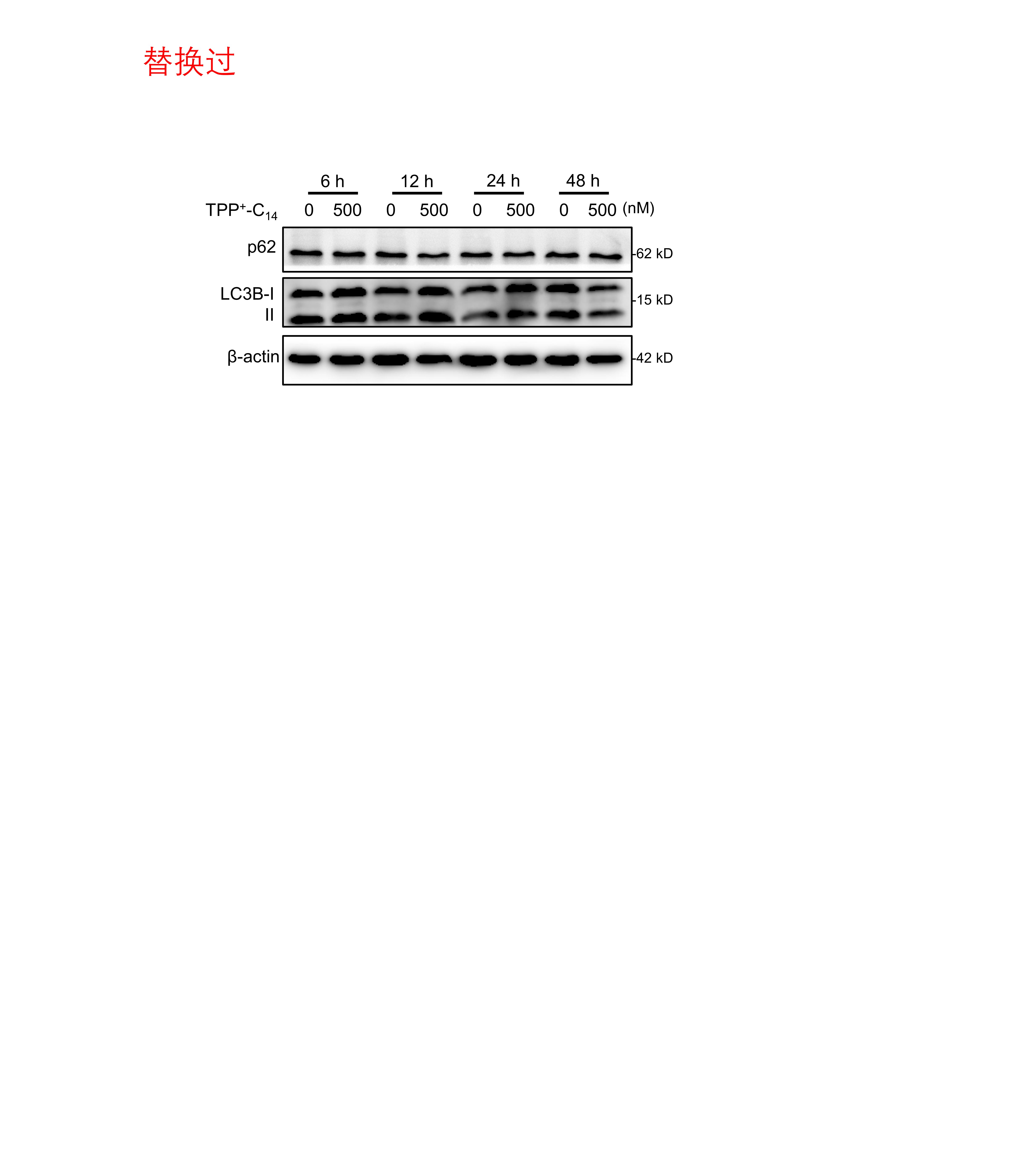


**Figure S16.** Immunoblot analysis of autophagy-related markers. HeLa cells were treated with TPP^+^-C_14_ (500 nM) for 6 h, 12 h, 24 h, or 48 h, followed by cell lysis and immunoblotting for LC3B, p62, and β-actin.


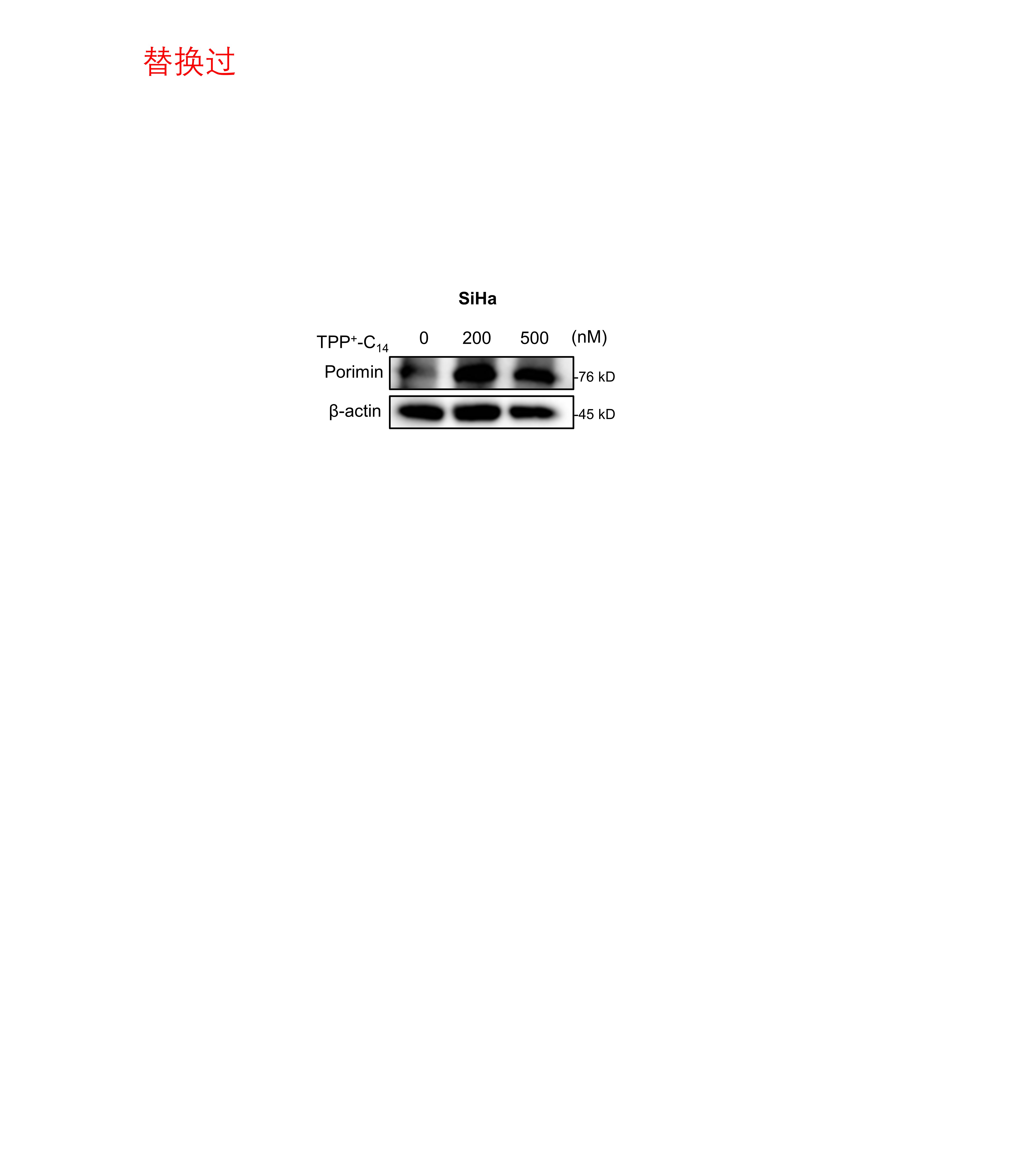


**Figure S17.** Immunoblot analysis of Porimin in SiHa cells after exposure to the corresponding treatment for 48 h.


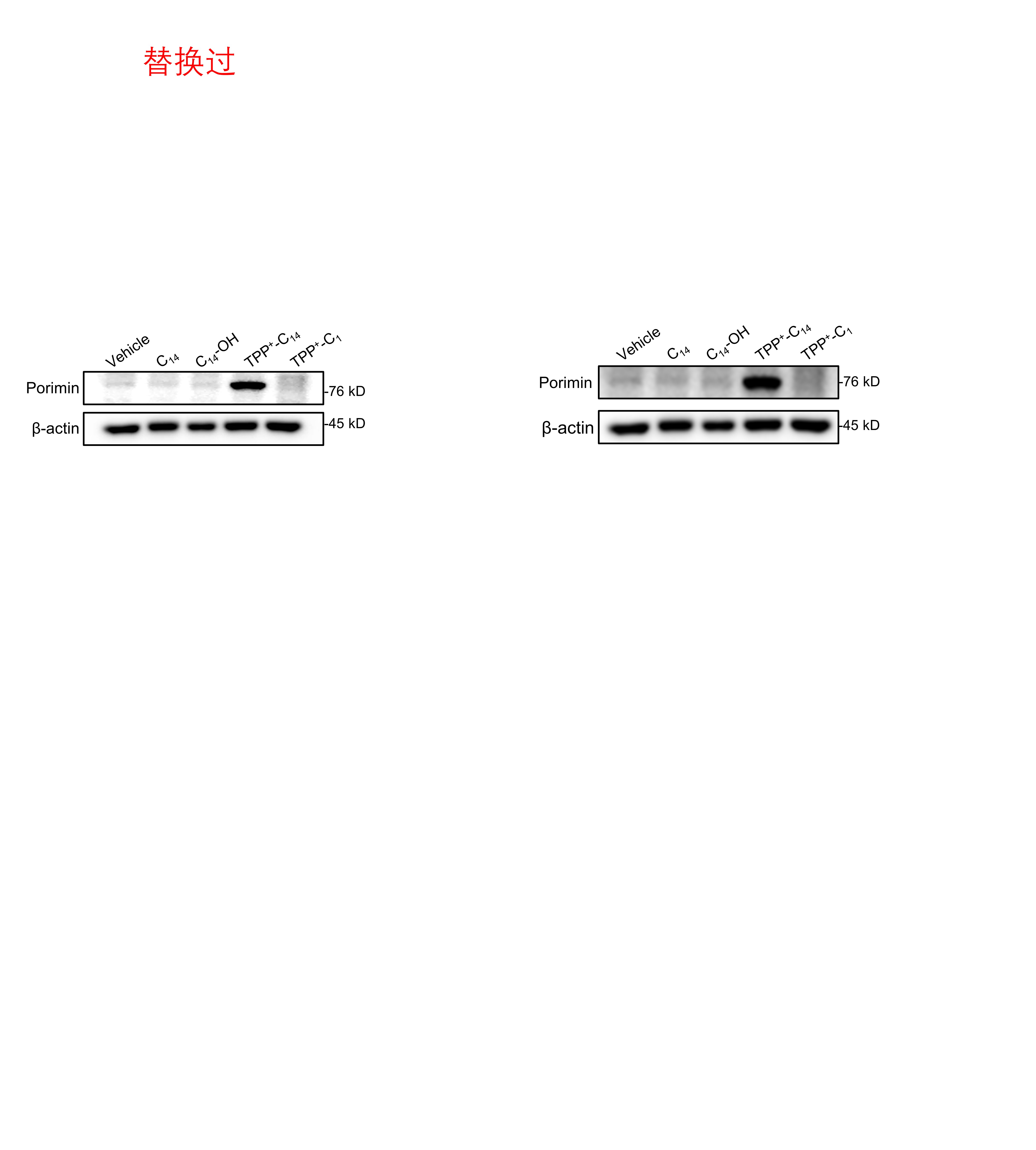


**Figure S18.** Western blot analysis of Porimin. HeLa cells were treated with 500 nM TPP^+^-C_14_, TPP^+^-C_1_, C_14_ (n-tetradecane), or C_14_-OH (1-tetradecanol) for 48 hours and then were lysed for immunoblotting.


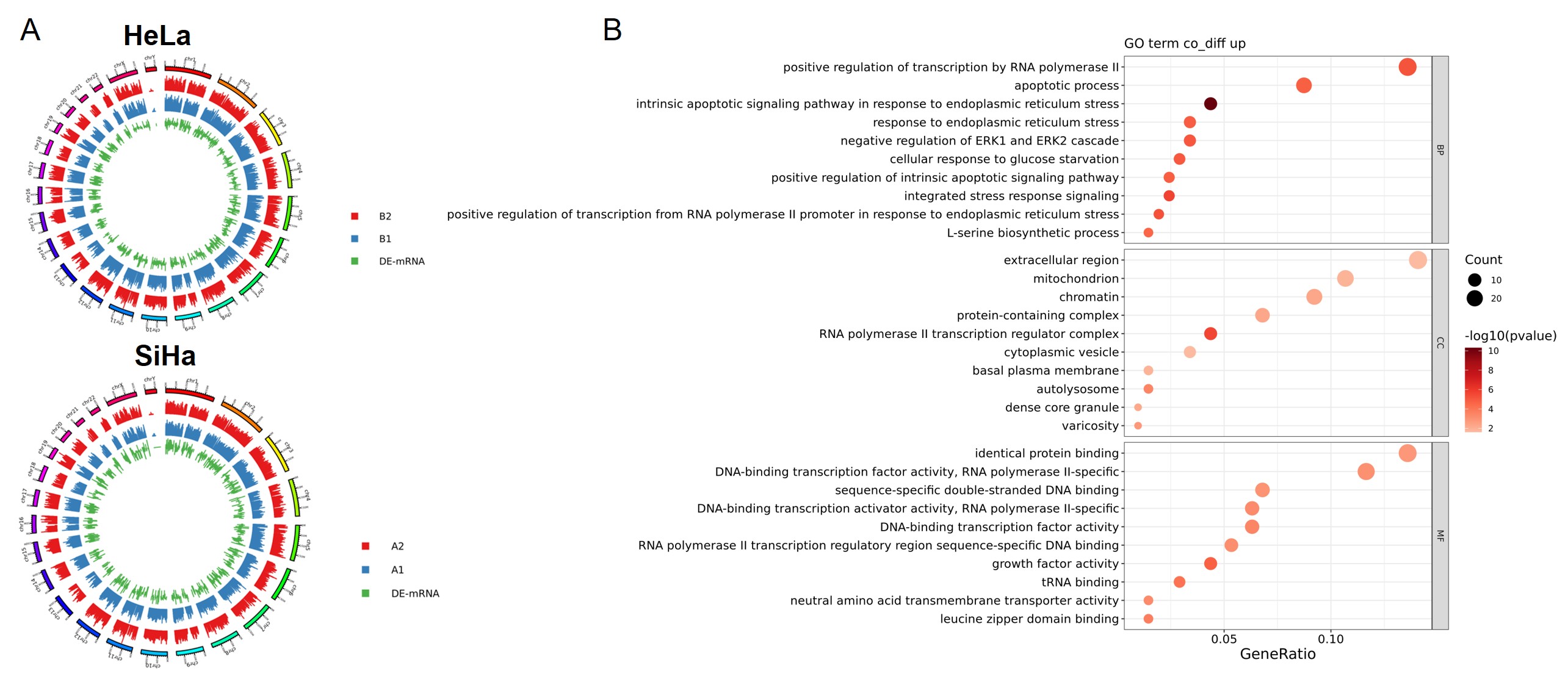


**Figure S19.** Transcriptomic analysis of HeLa and SiHa cells following TPP^+^-C_14_ treatment. A) Differentially expressed genes (DEGs) were identified in HeLa and SiHa cells treated with TPP^+^-C_14_ (500 nM) for 24 h. RNA sequencing was performed using the Illumina platform, and data were analyzed with edgeR (FDR < 0.05, |log_2_ fold change| >1.2). B) Gene Ontology (GO) biological process enrichment analysis of co-upregulated DEGs (top 20 terms, p < 0.05).


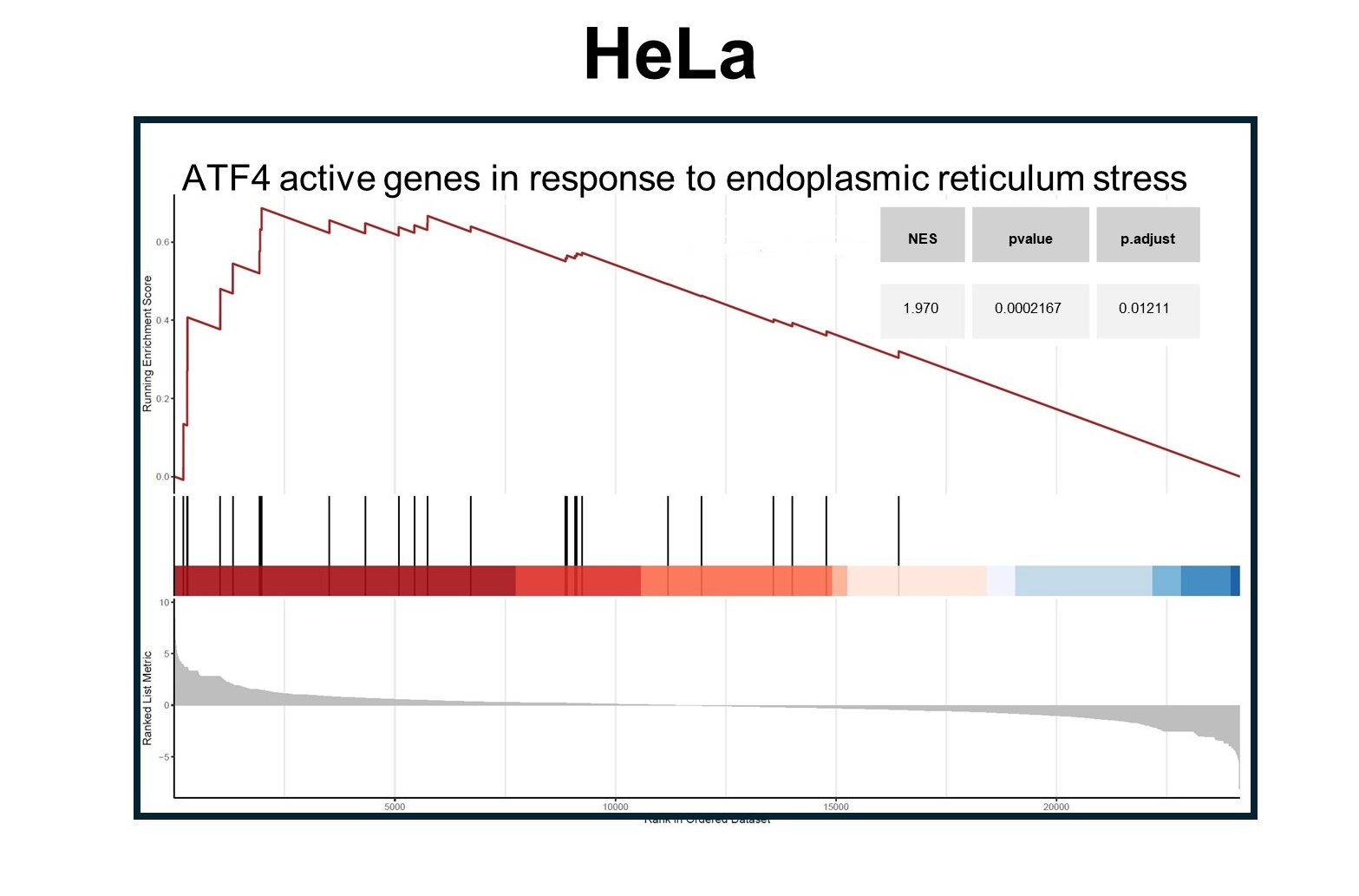


**Figure S20.** GSEA of significant pathways in HeLa cells that were treated with TPP^+^-C_14_ (500 nM, 24 h). ATF4 active genes in response to the endoplasmic reticulum stress pathway in HeLa cells. (|NES| > 1, p < 0.05, adjusted p < 0.25).


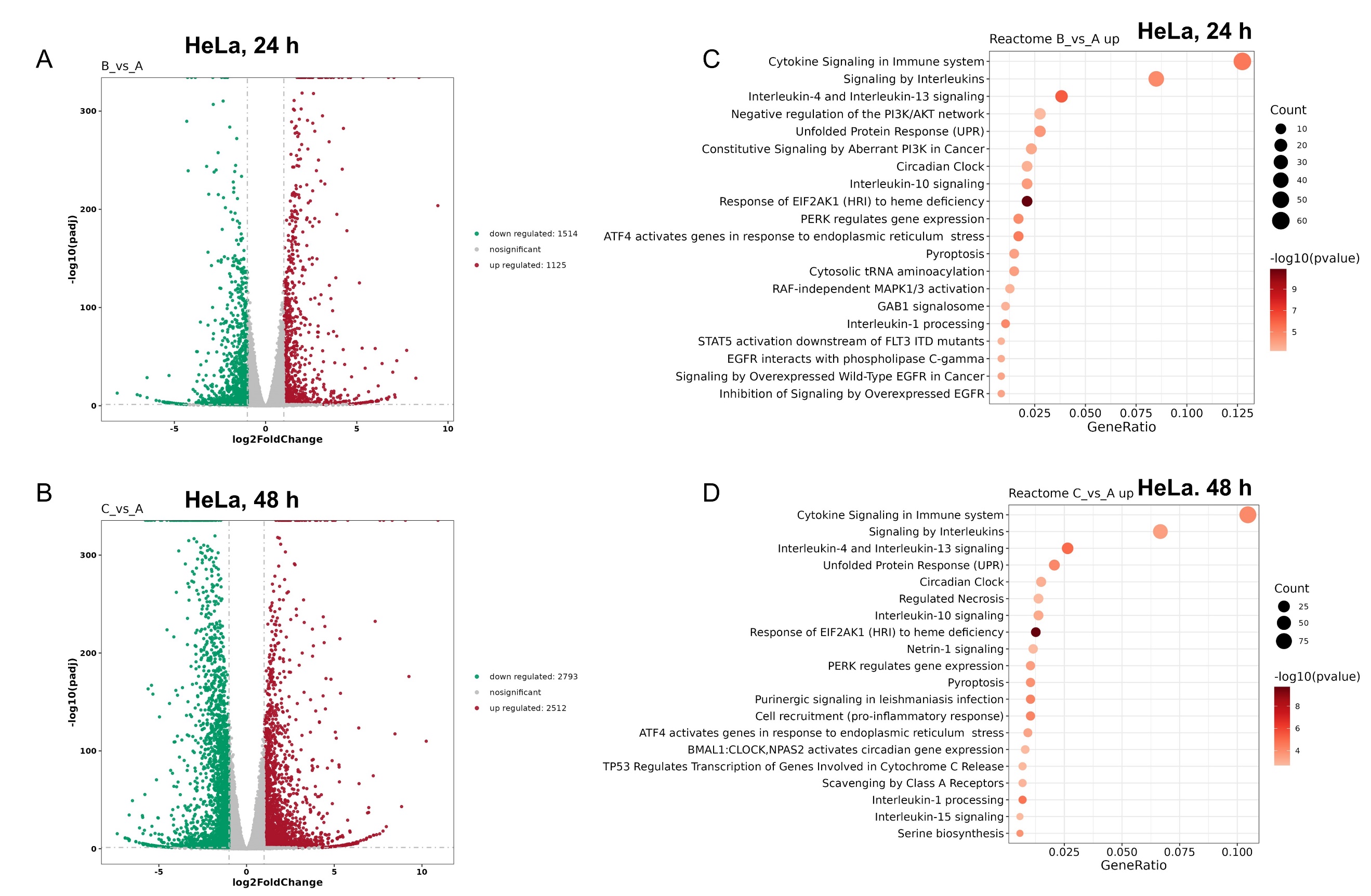


**Figure S21.** Transcriptomic profiling of HeLa cells treated with 500 nM TPP^+^-C_14_ at various time points. A, B) Volcano plots depicting DEGs in HeLa cells treated with TPP^+^-C_14_ for 24 h A) or 48 h B). C, D) Reactome pathway enrichment analysis of upregulated DEGs in HeLa cells treated with TPP^+^-C_14_ for 24 h C) or 48 h D).


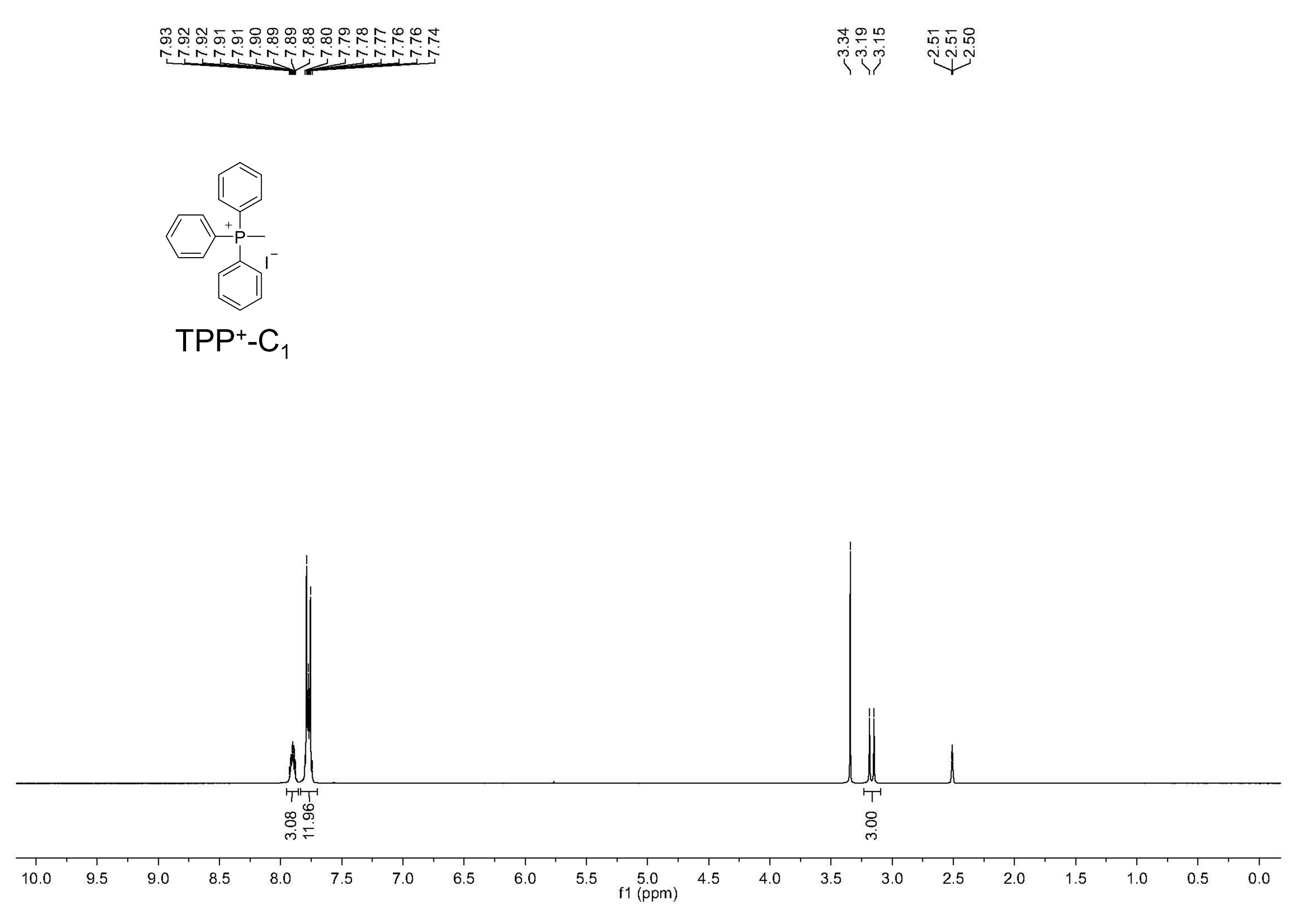


**Figure S22.** ^1^HNMR spectrum of TPP^+^-C_1_.


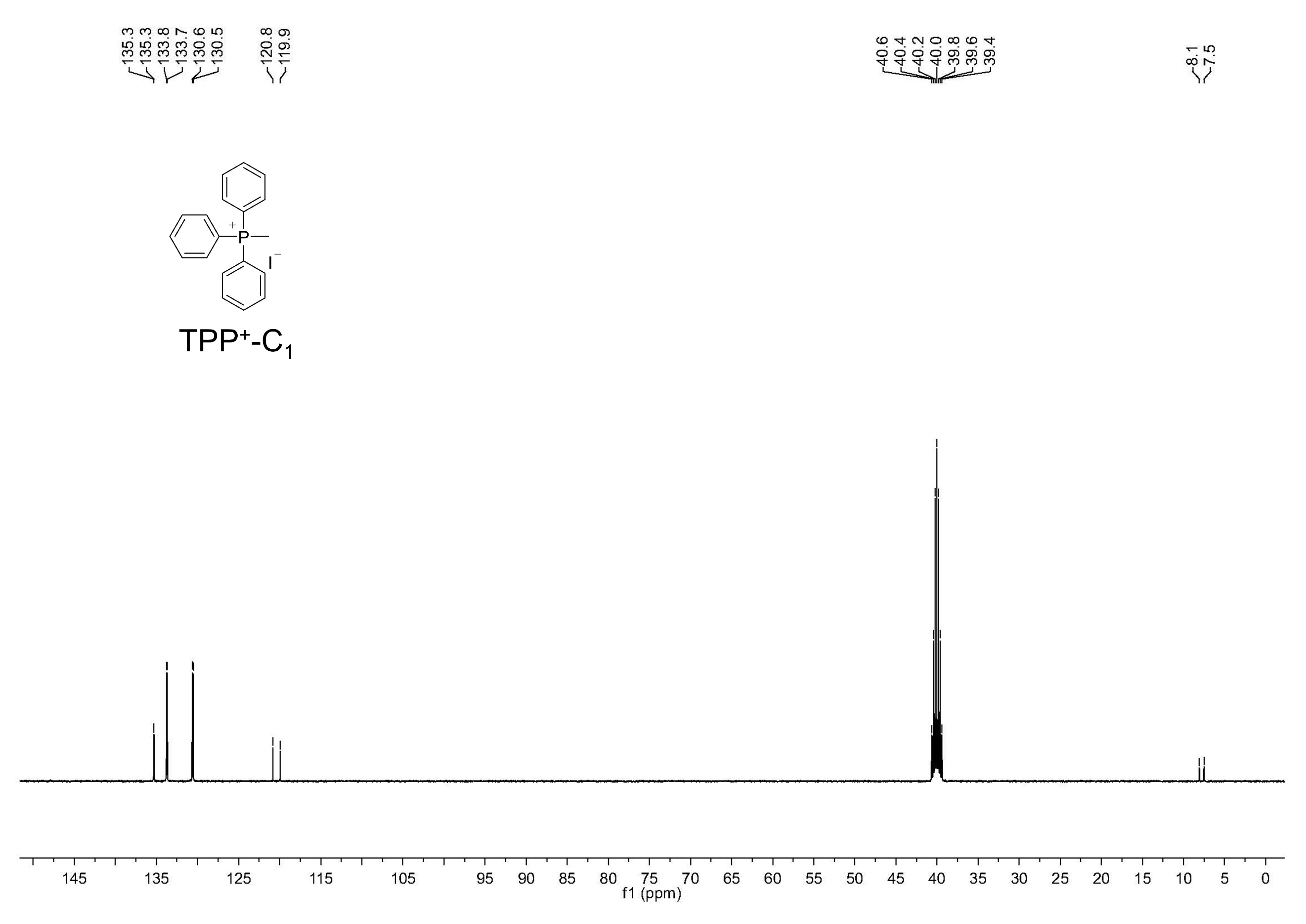


**Figure S23.** ^13^CNMR spectrum of TPP^+^-C_1_.


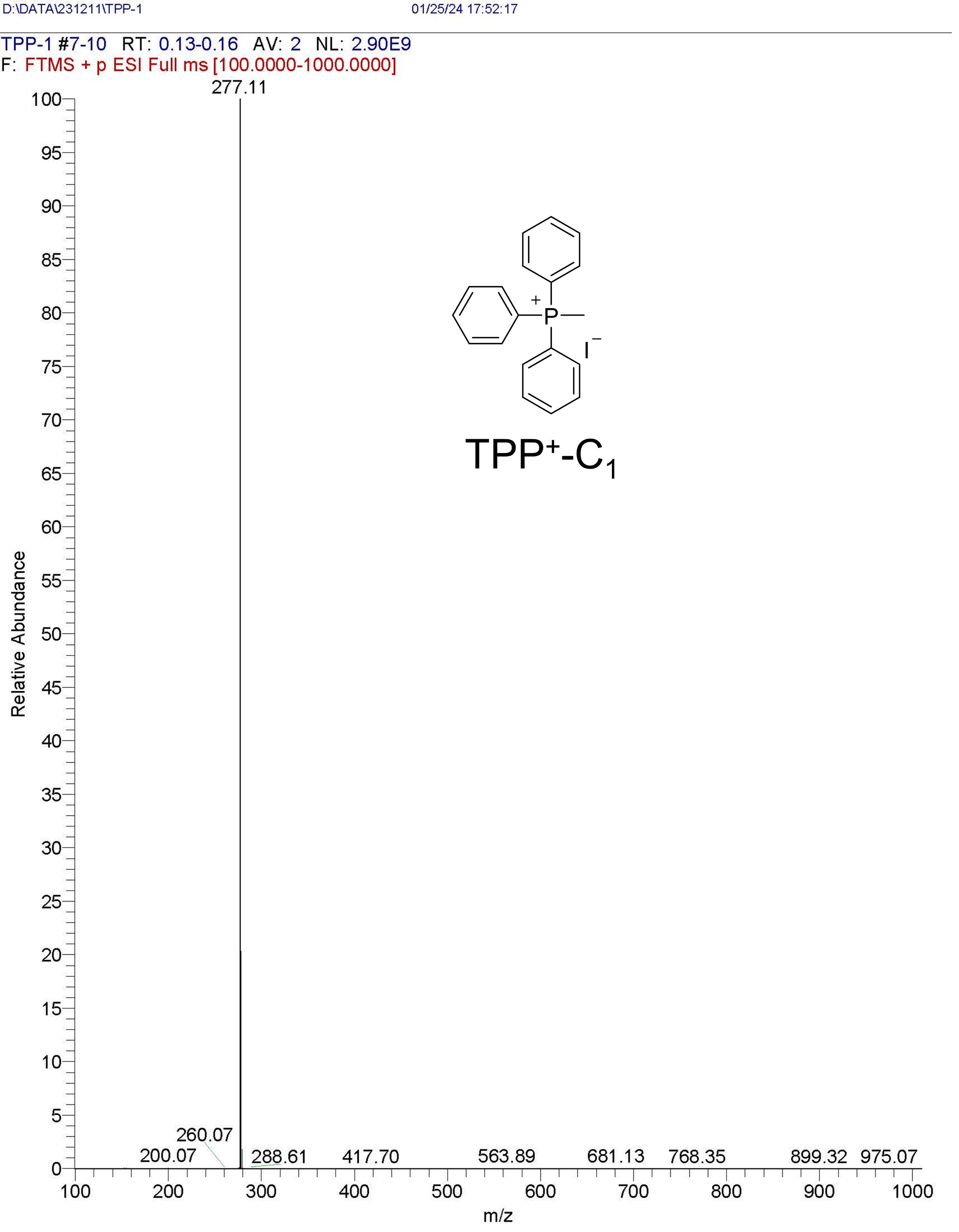


**Figure S24.** HR-MS spectrum of TPP^+^-C_1_.


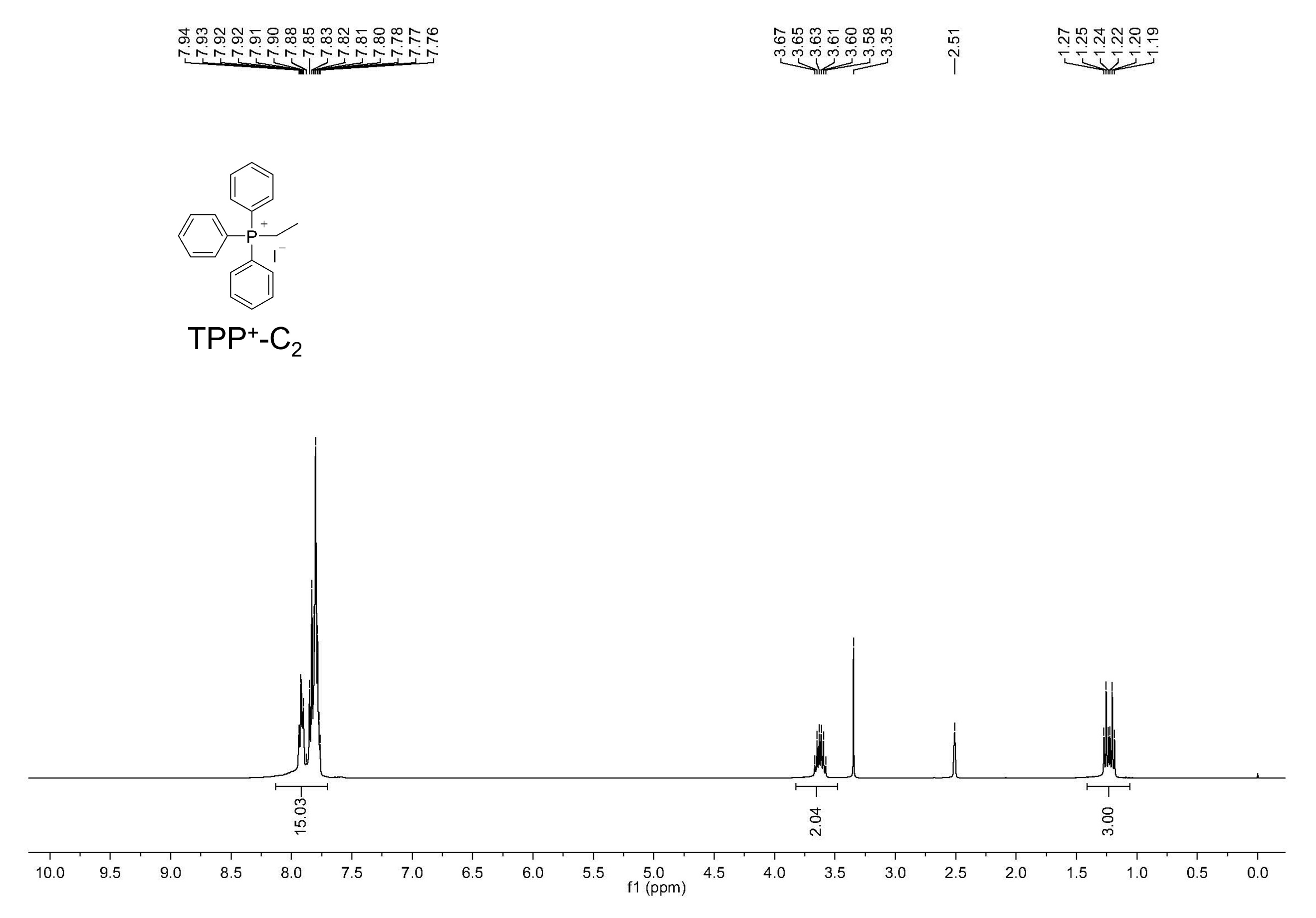


**Figure S25.** ^1^HNMR spectrum of TPP^+^-C_2_.


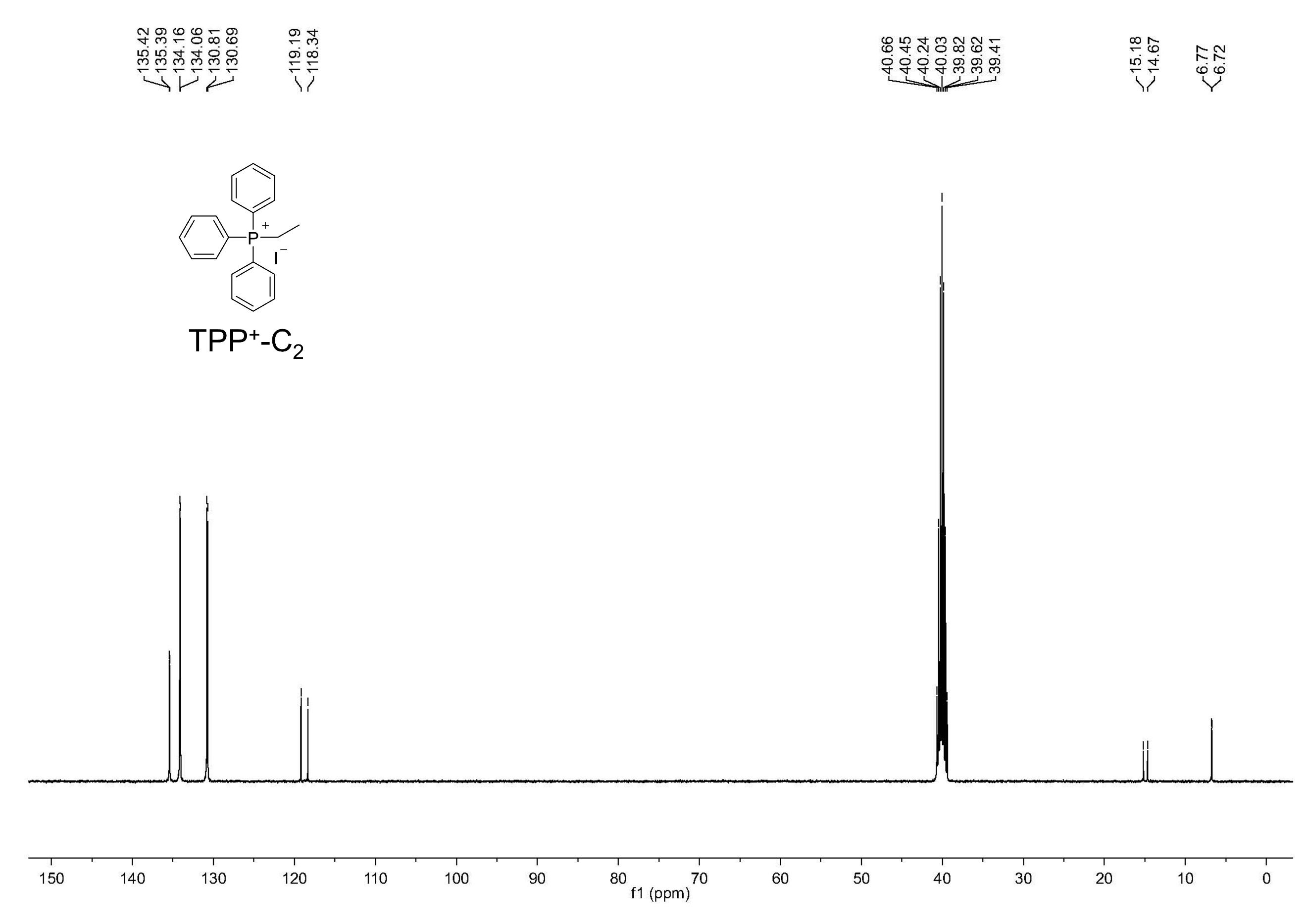


**Figure S26.** ^13^CNMR spectrum of TPP^+^-C_2_.


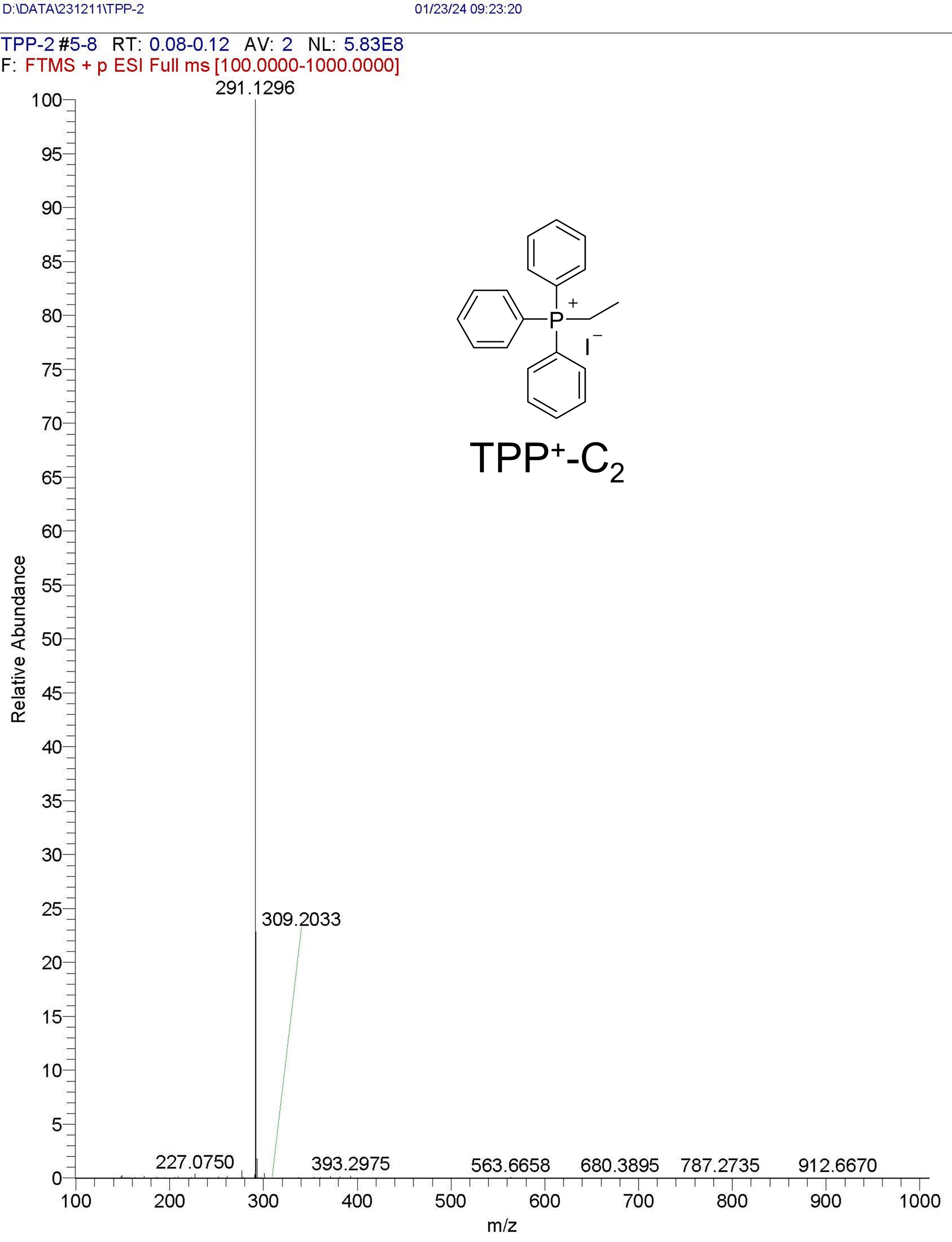


**Figure S27.** HR-MS spectrum of TPP^+^-C_2_.


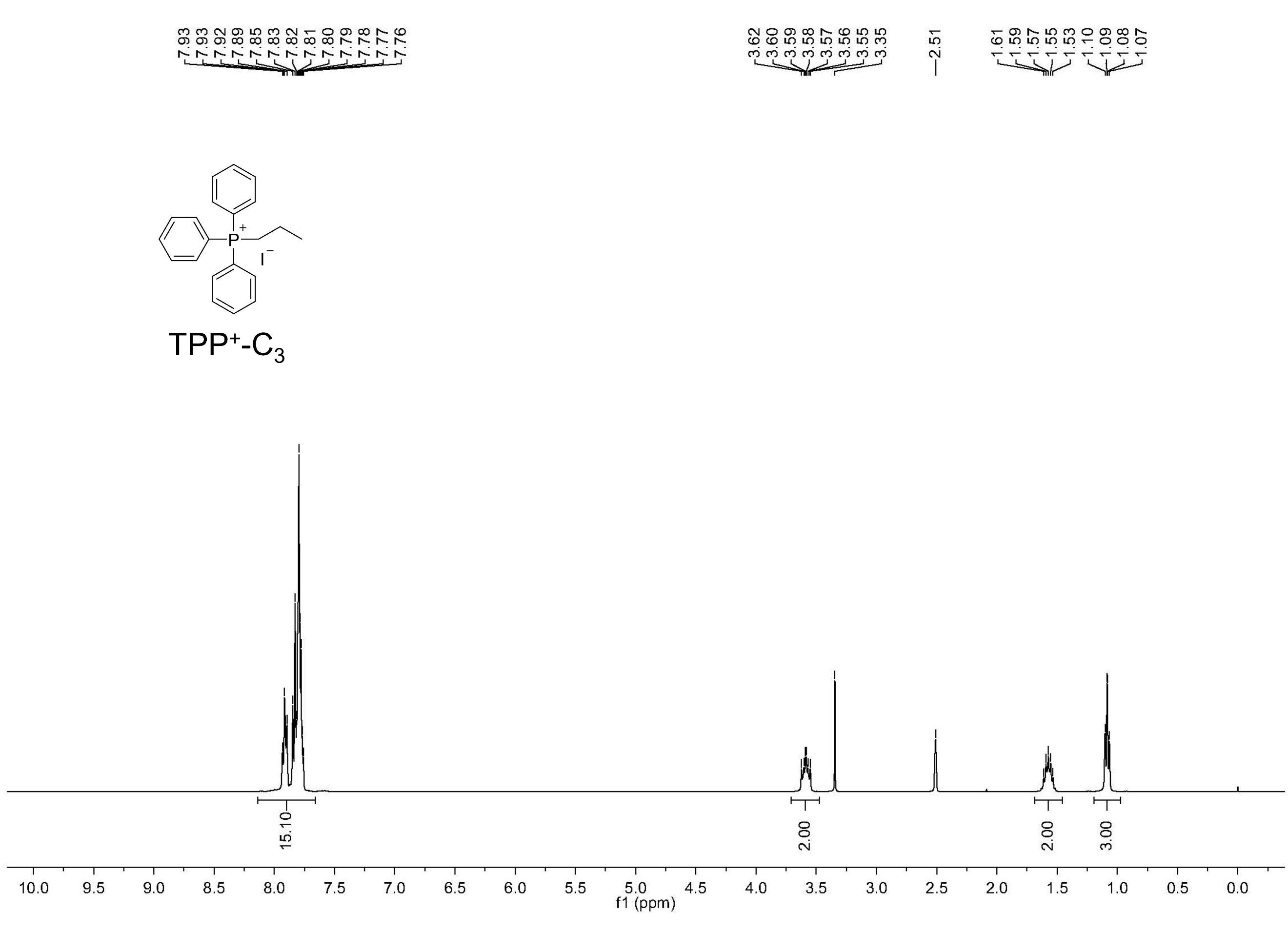


**Figure S28.** ^1^HNMR spectrum of TPP^+^-C_3_.


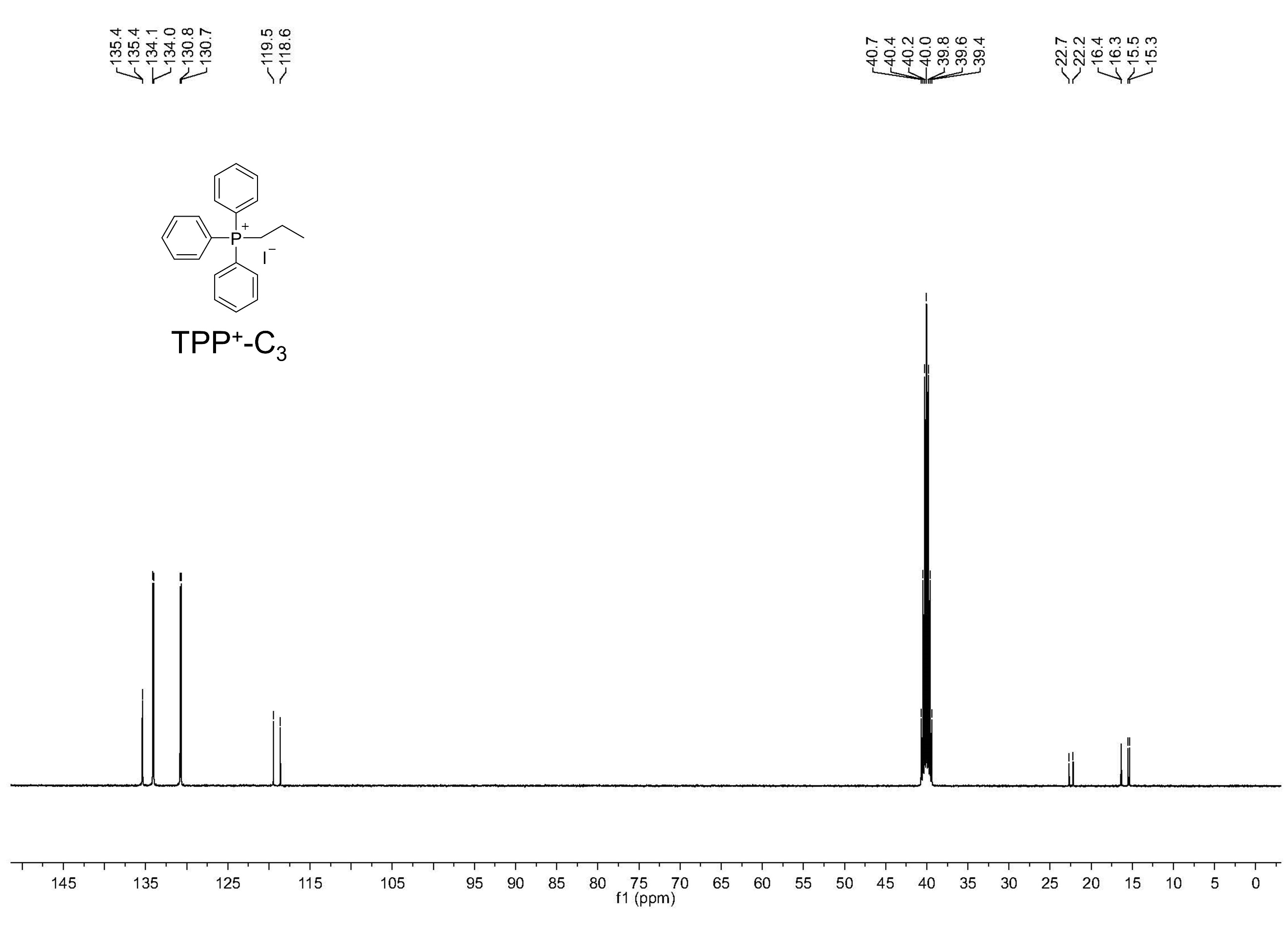


**Figure S29.** ^13^CNMR spectrum of TPP^+^-C_3_.


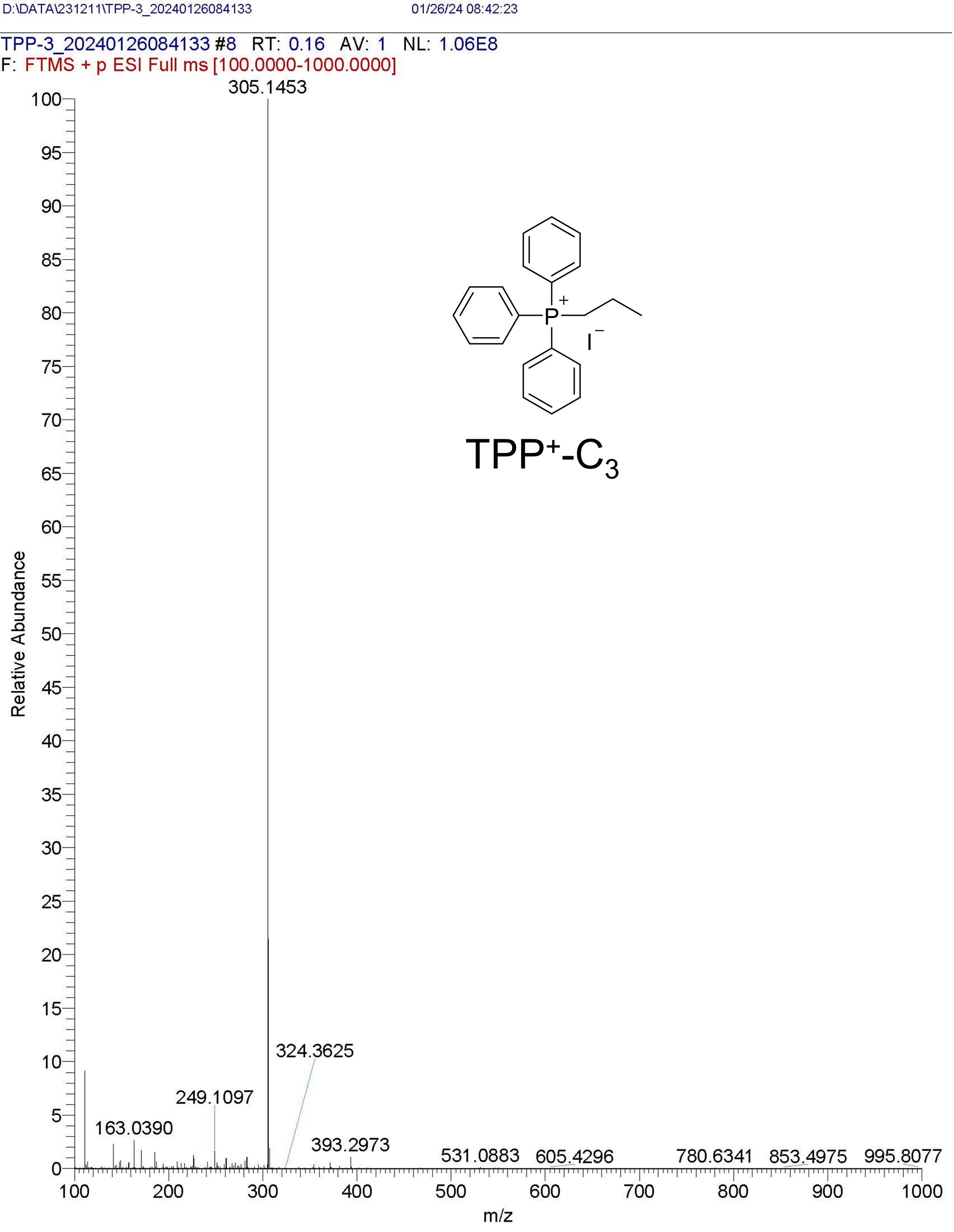


**Figure S30.** HR-MS spectrum of TPP^+^-C_3_.


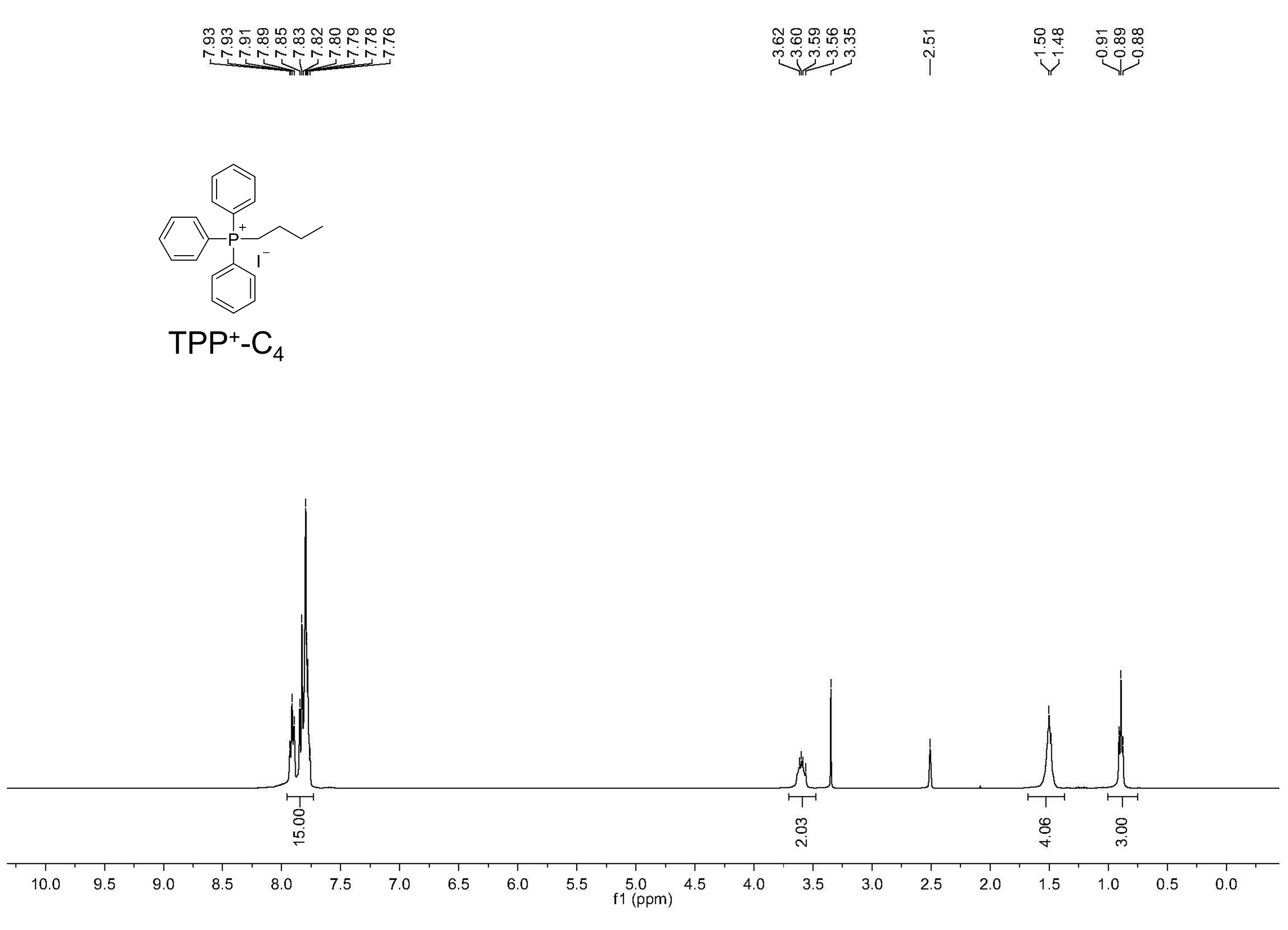


**Figure S31.** ^1^HNMR spectrum of TPP^+^-C_4_.


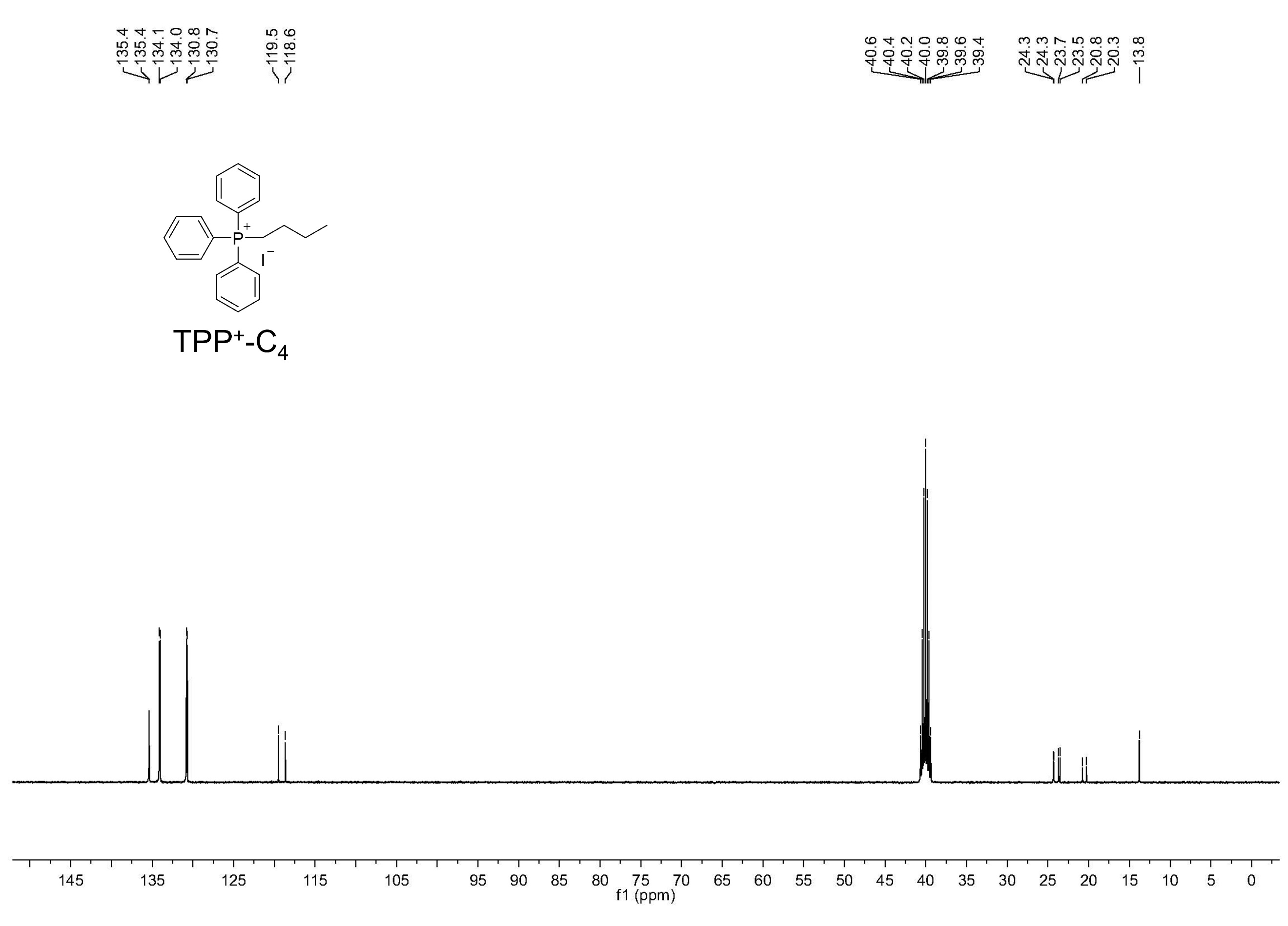


**Figure S32.** ^13^CNMR spectrum of TPP^+^-C_4_.


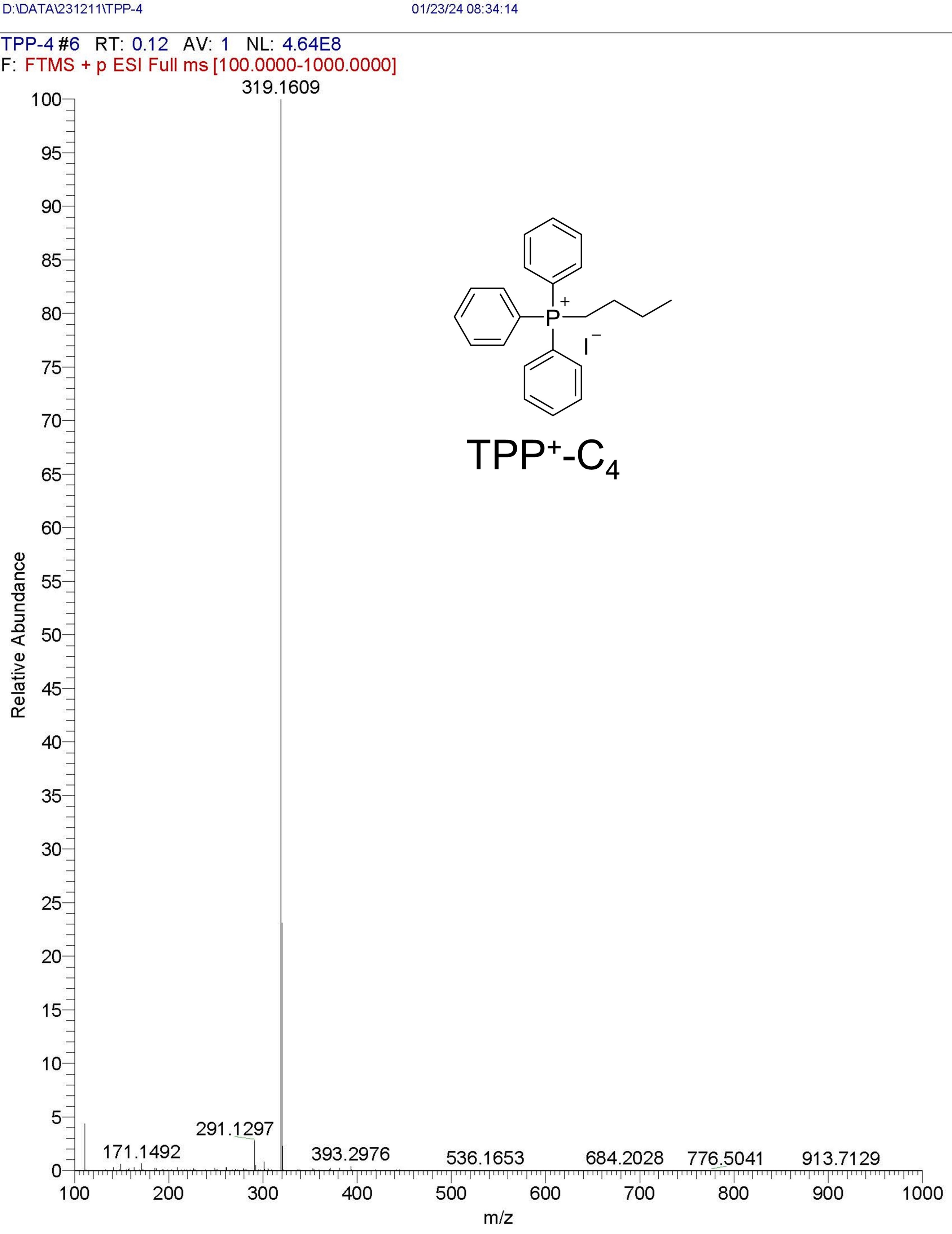


**Figure S33.** HR-MS spectrum of TPP^+^-C_4_.


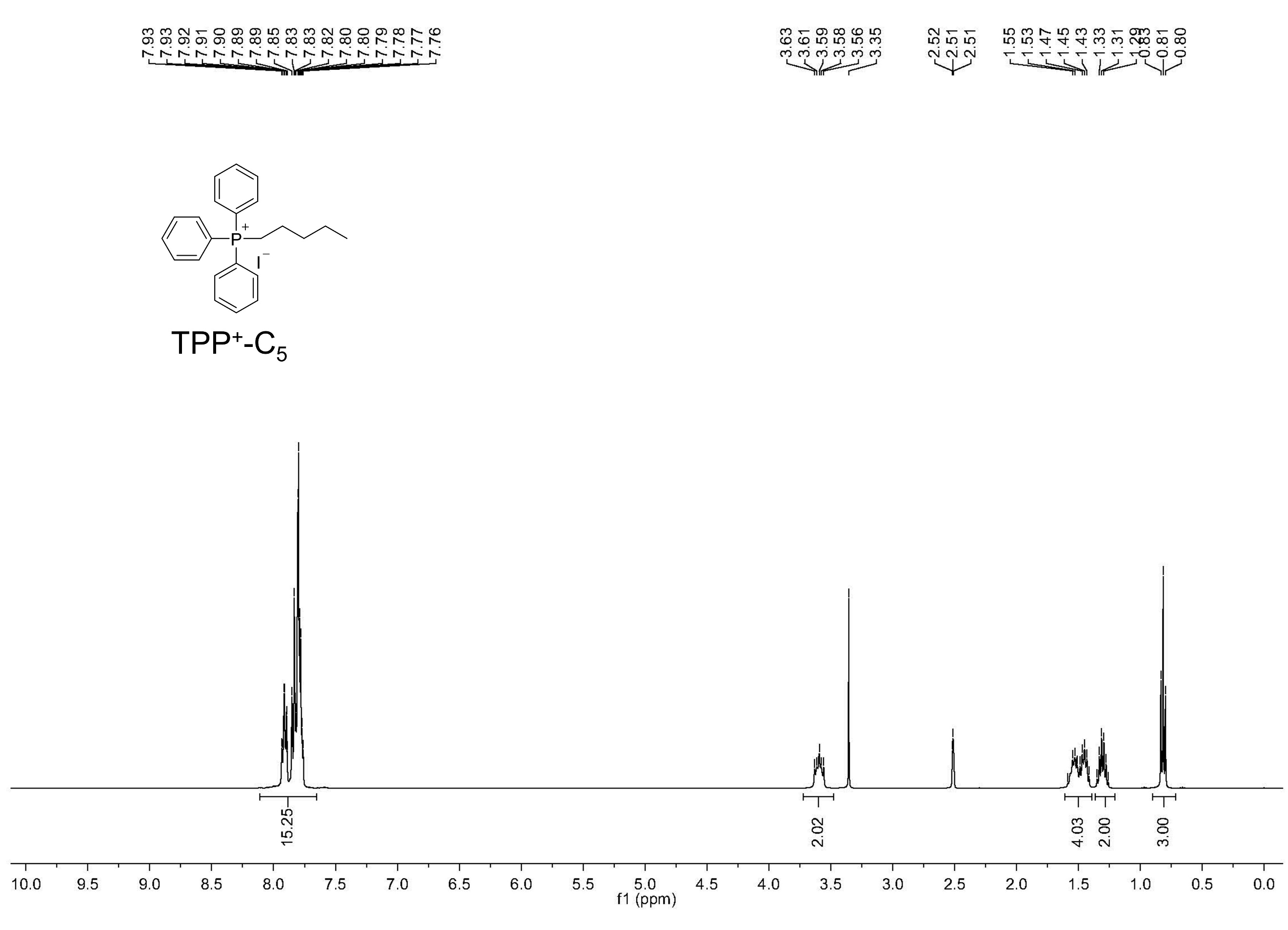


**Figure S34.** ^1^HNMR spectrum of TPP^+^-C_5_.


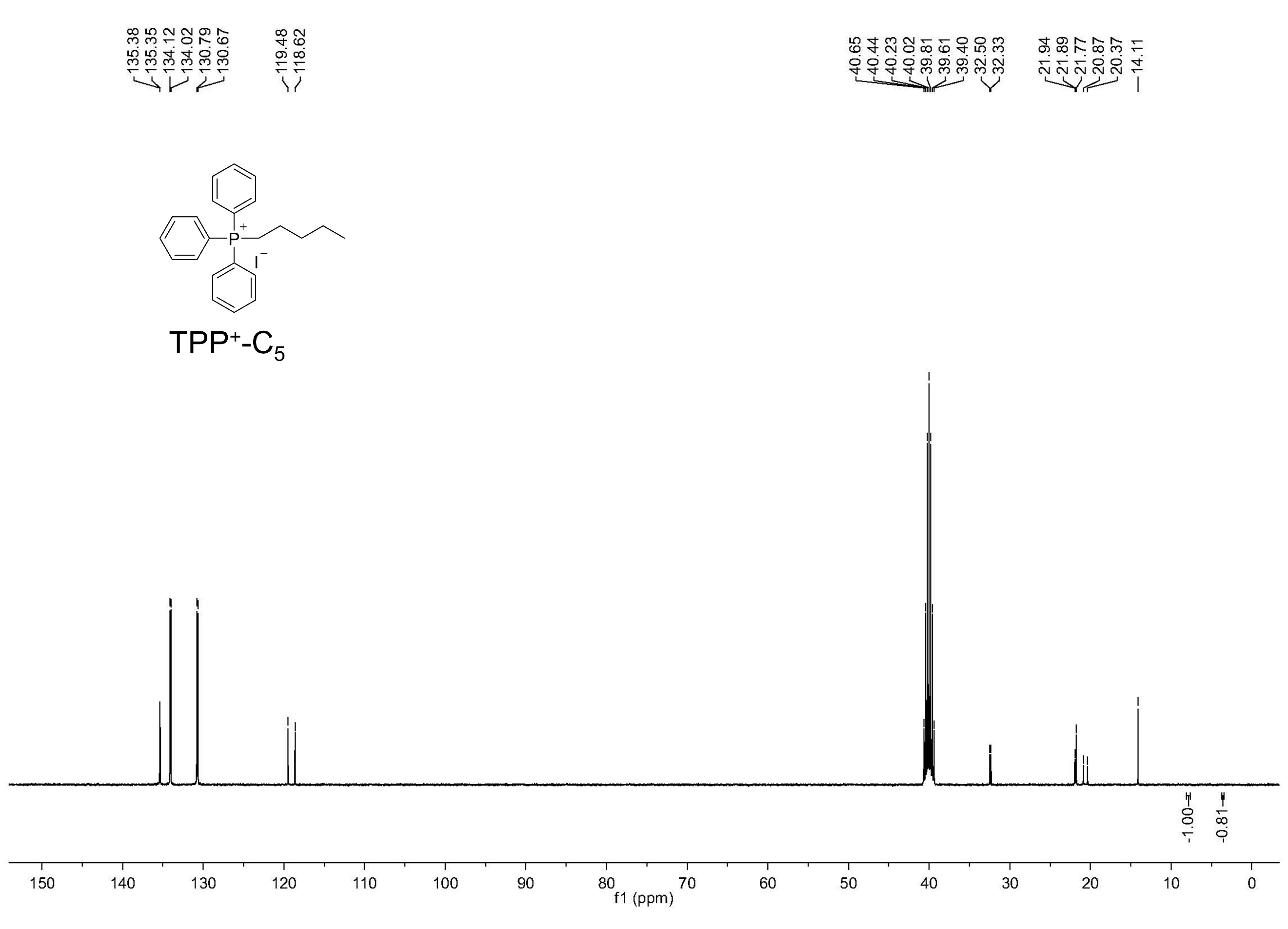


**Figure S35.** ^13^CNMR spectrum of TPP^+^-C_5_.


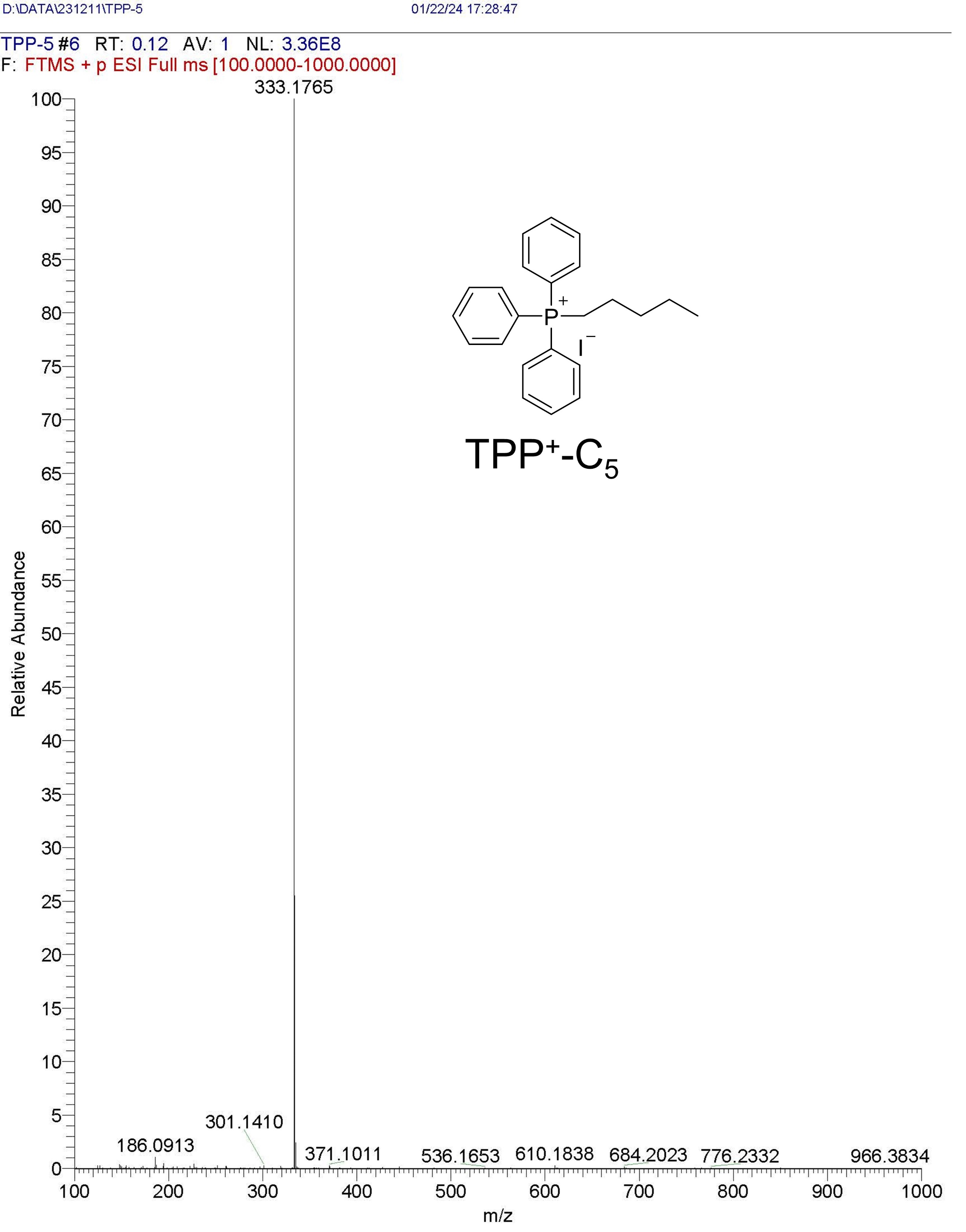


**Figure S36.** HR-MS spectrum of TPP^+^-C_5_.

**
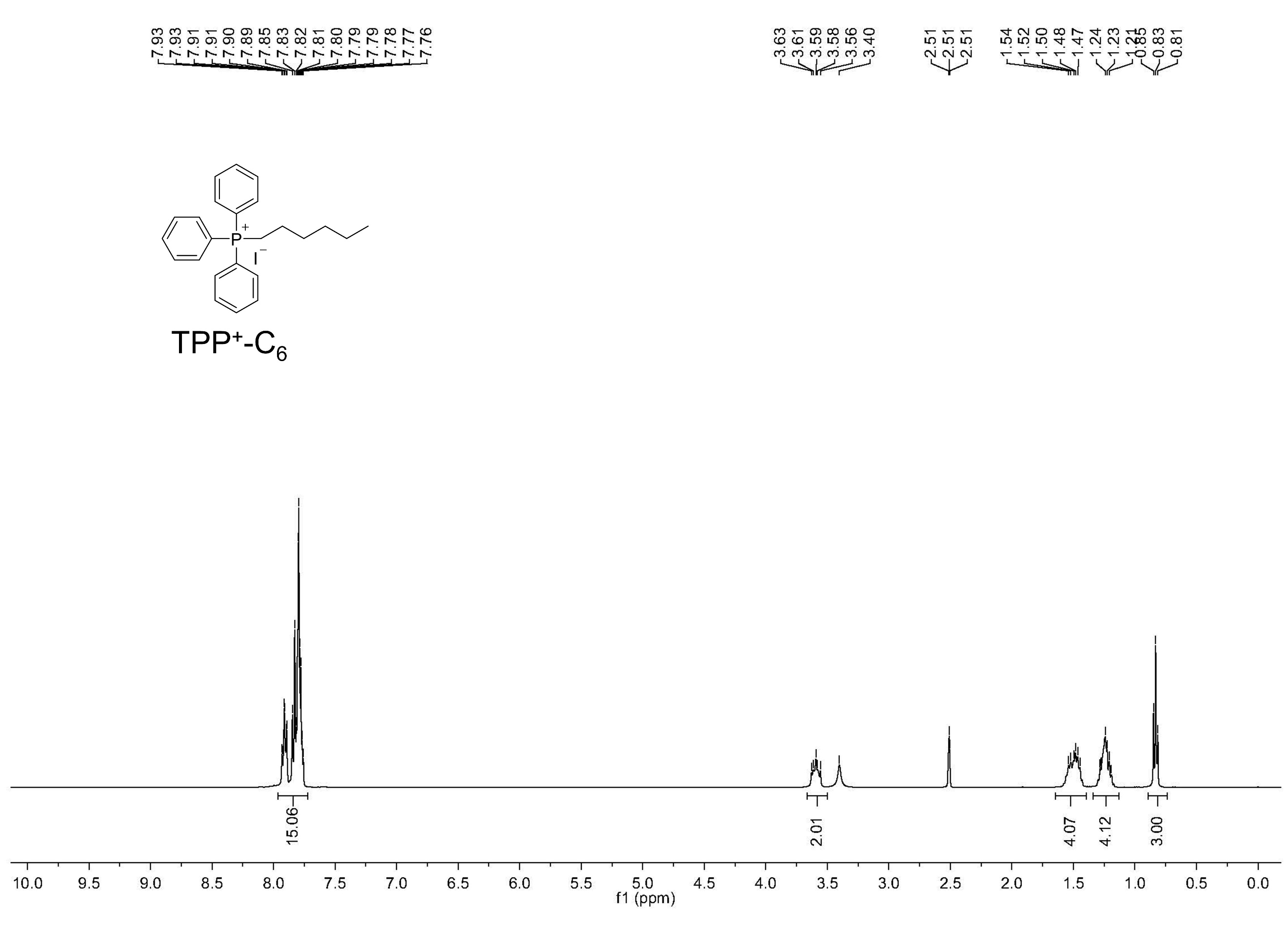
**

**Figure S37.** ^1^HNMR spectrum of TPP^+^-C_6_.


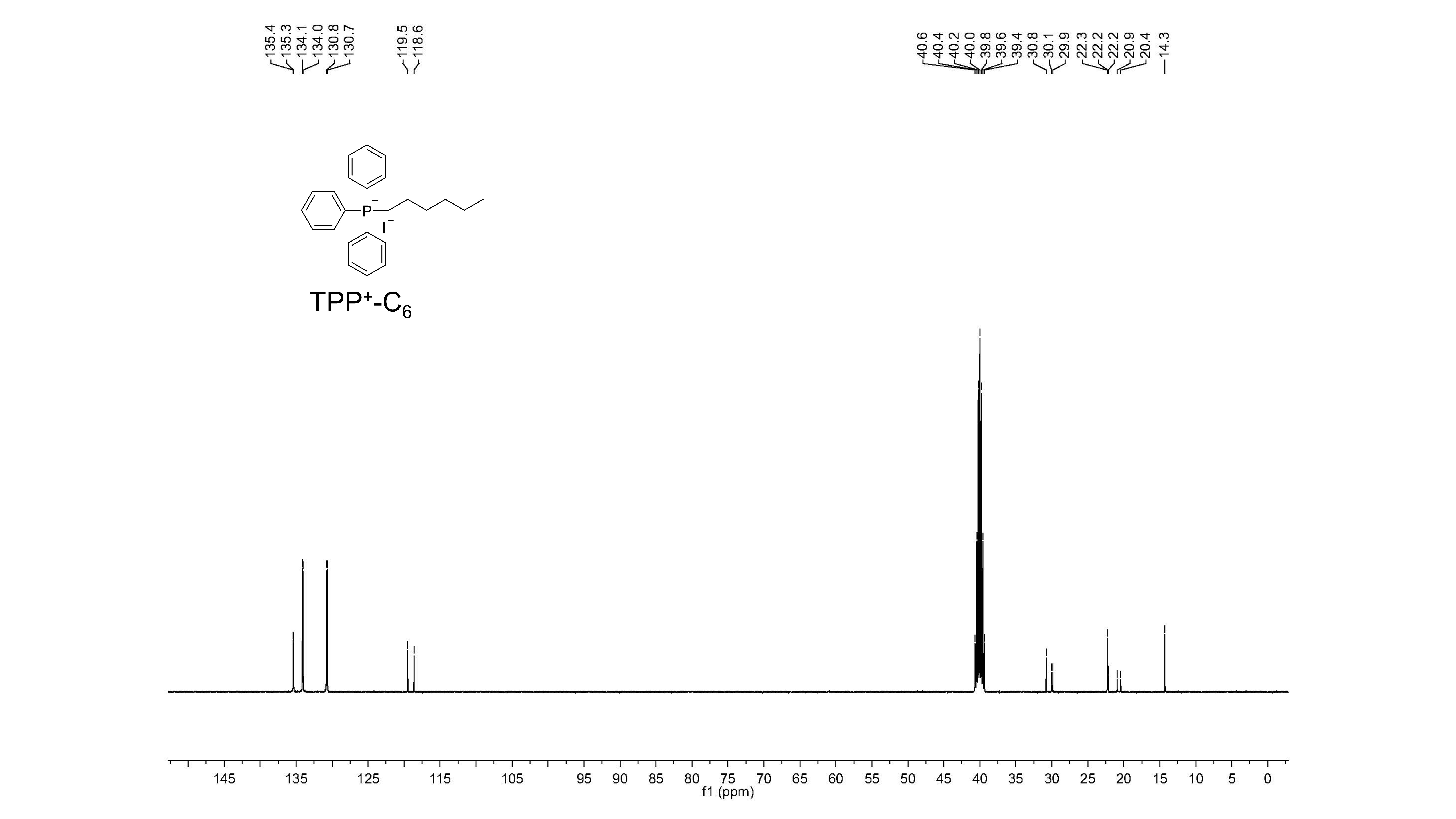


**Figure S38.** ^13^CNMR spectrum of TPP^+^-C_6_.


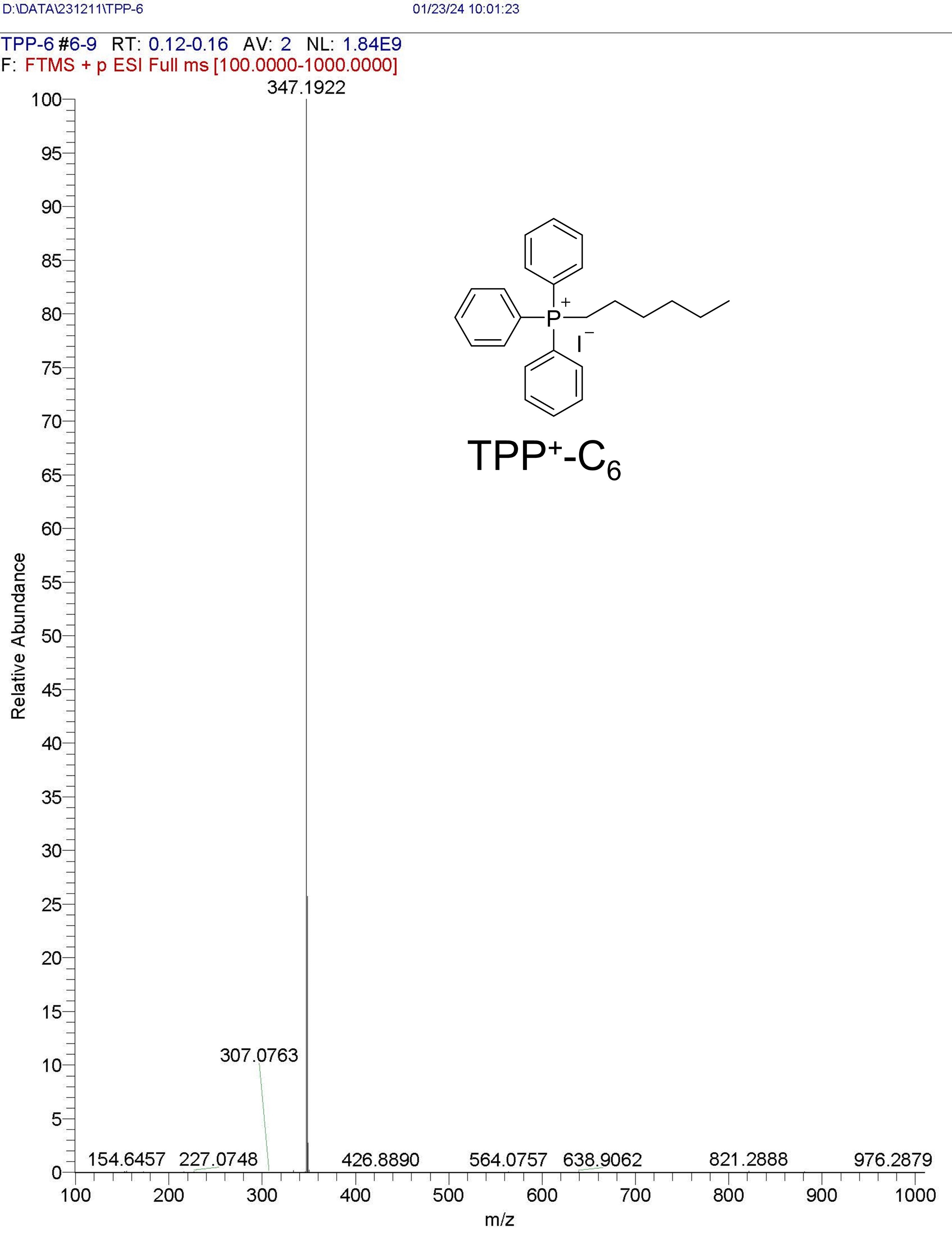


**Figure S39.** HR-MS spectrum of TPP^+^-C_6_.


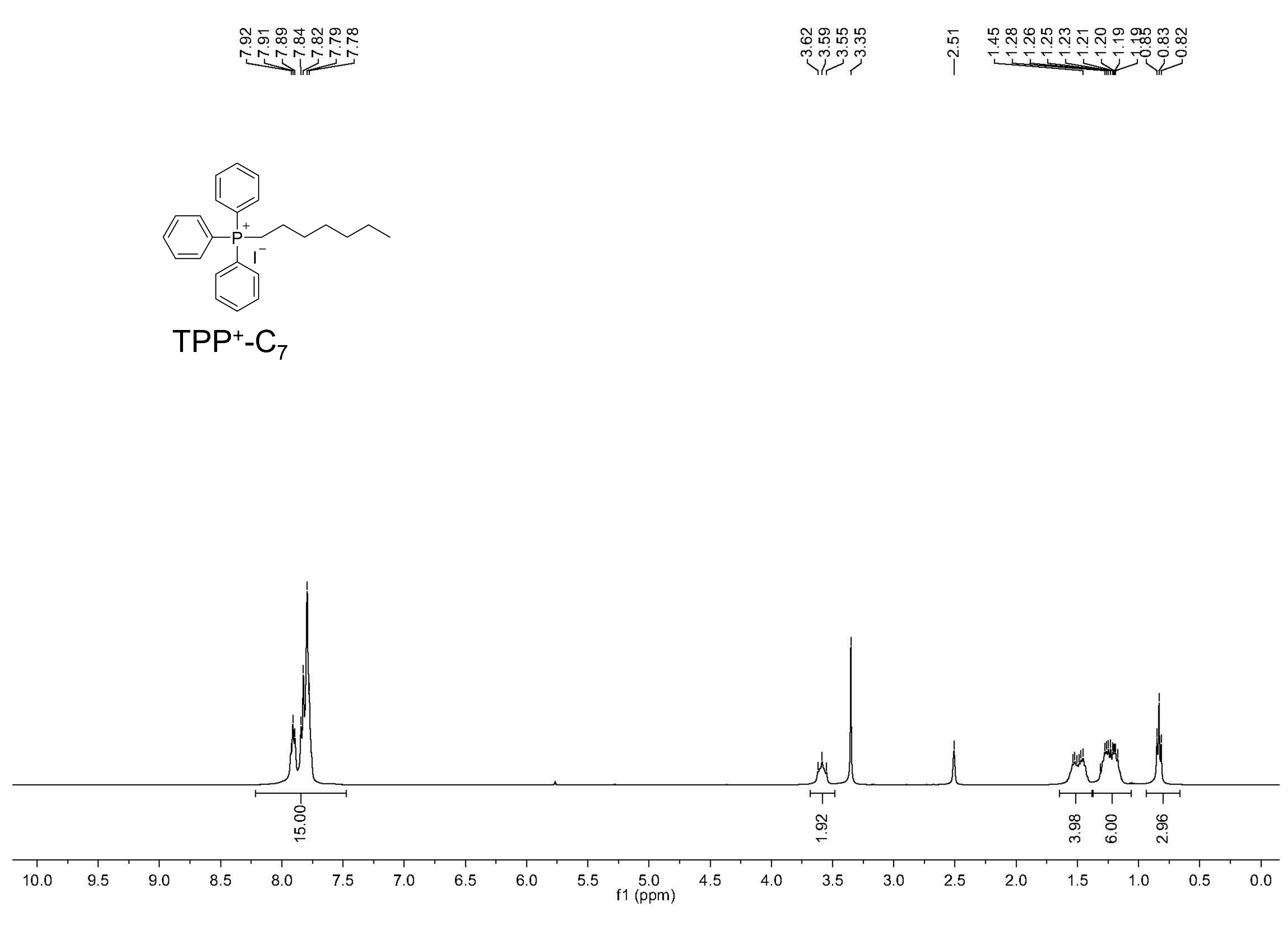


**Figure S40.** ^1^HNMR spectrum of TPP^+^-C_7_.


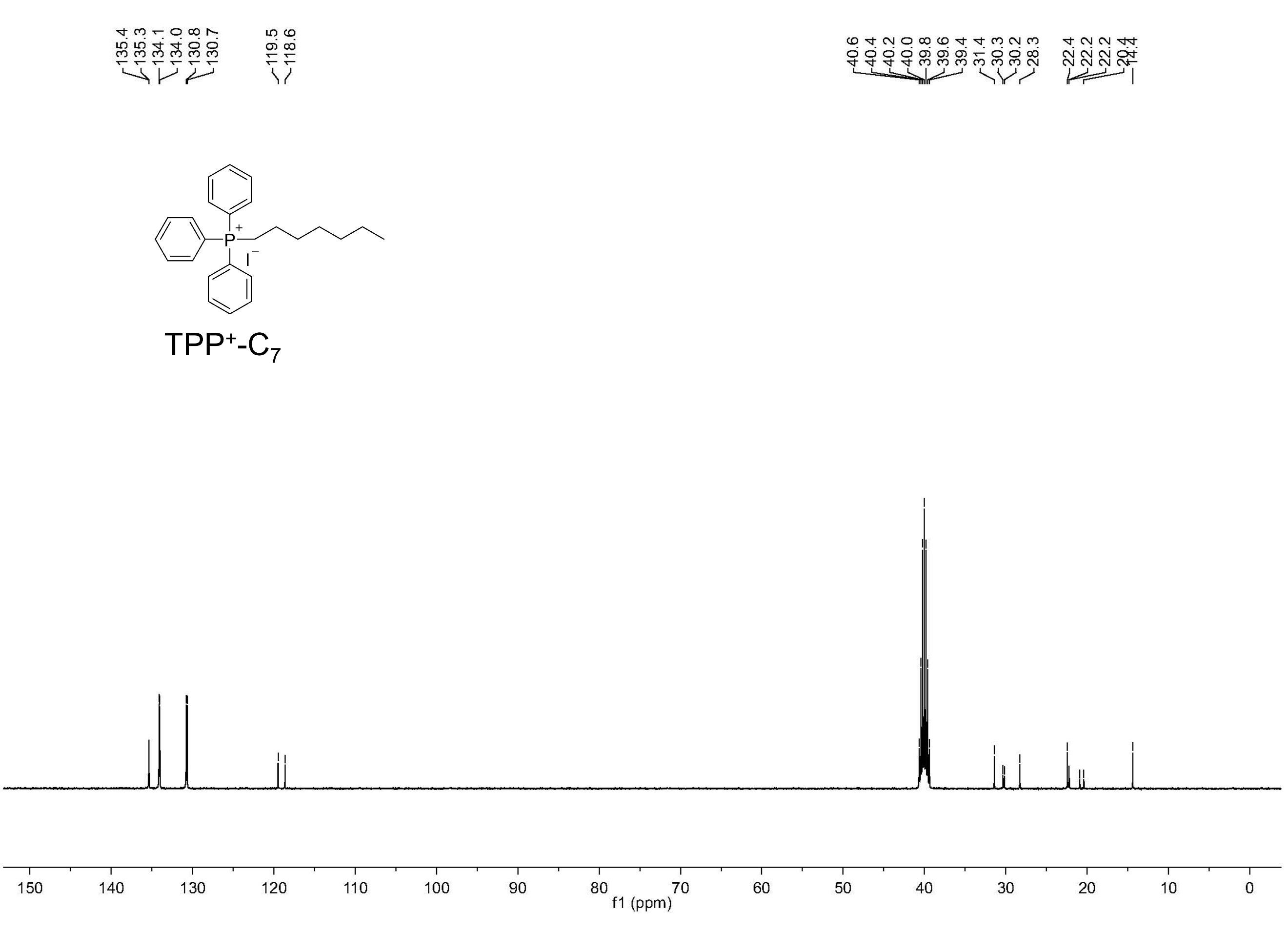


**Figure S41.** ^13^CNMR spectrum of TPP^+^-C_7_.


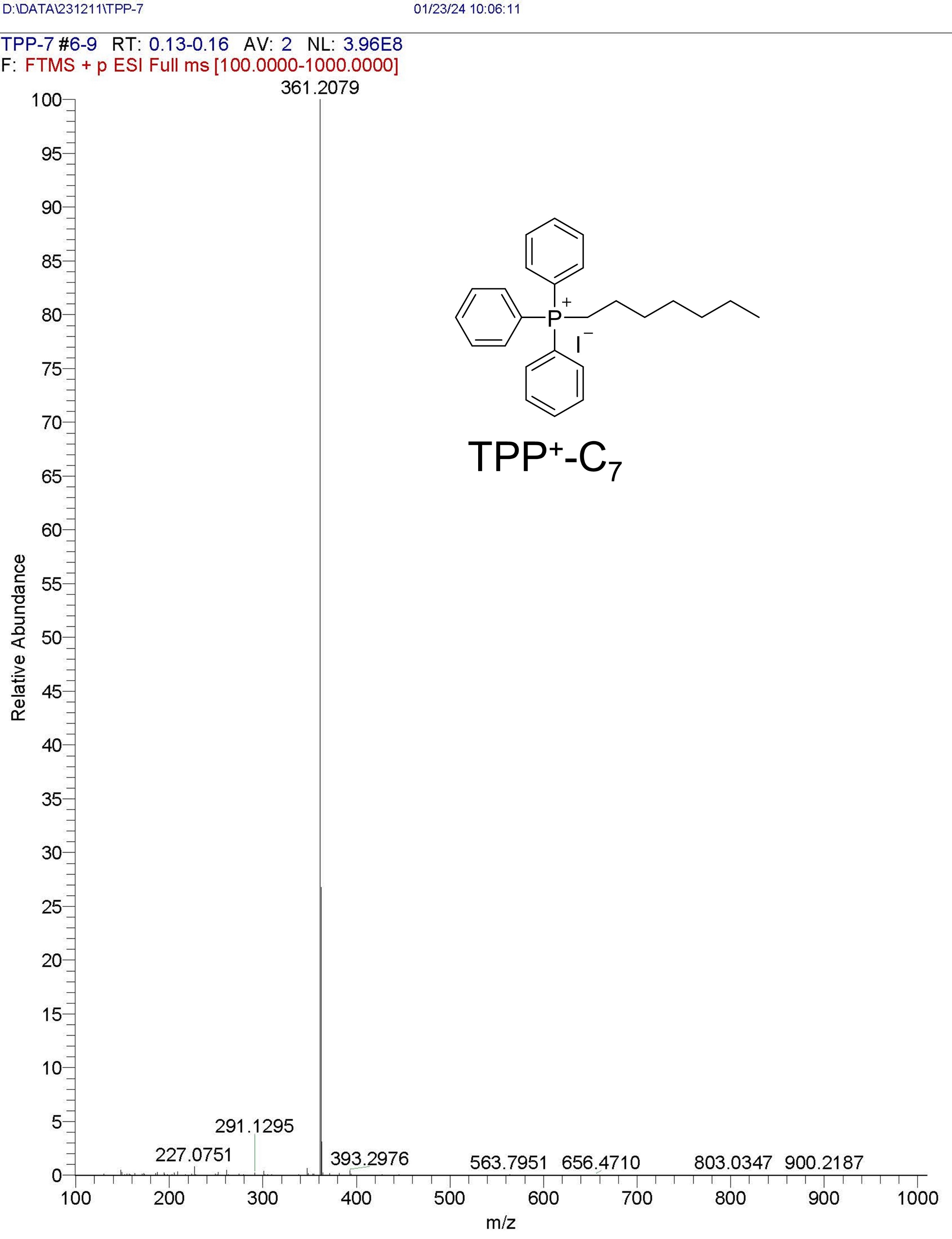


**Figure S42.** HR-MS spectrum of TPP^+^-C_7_.


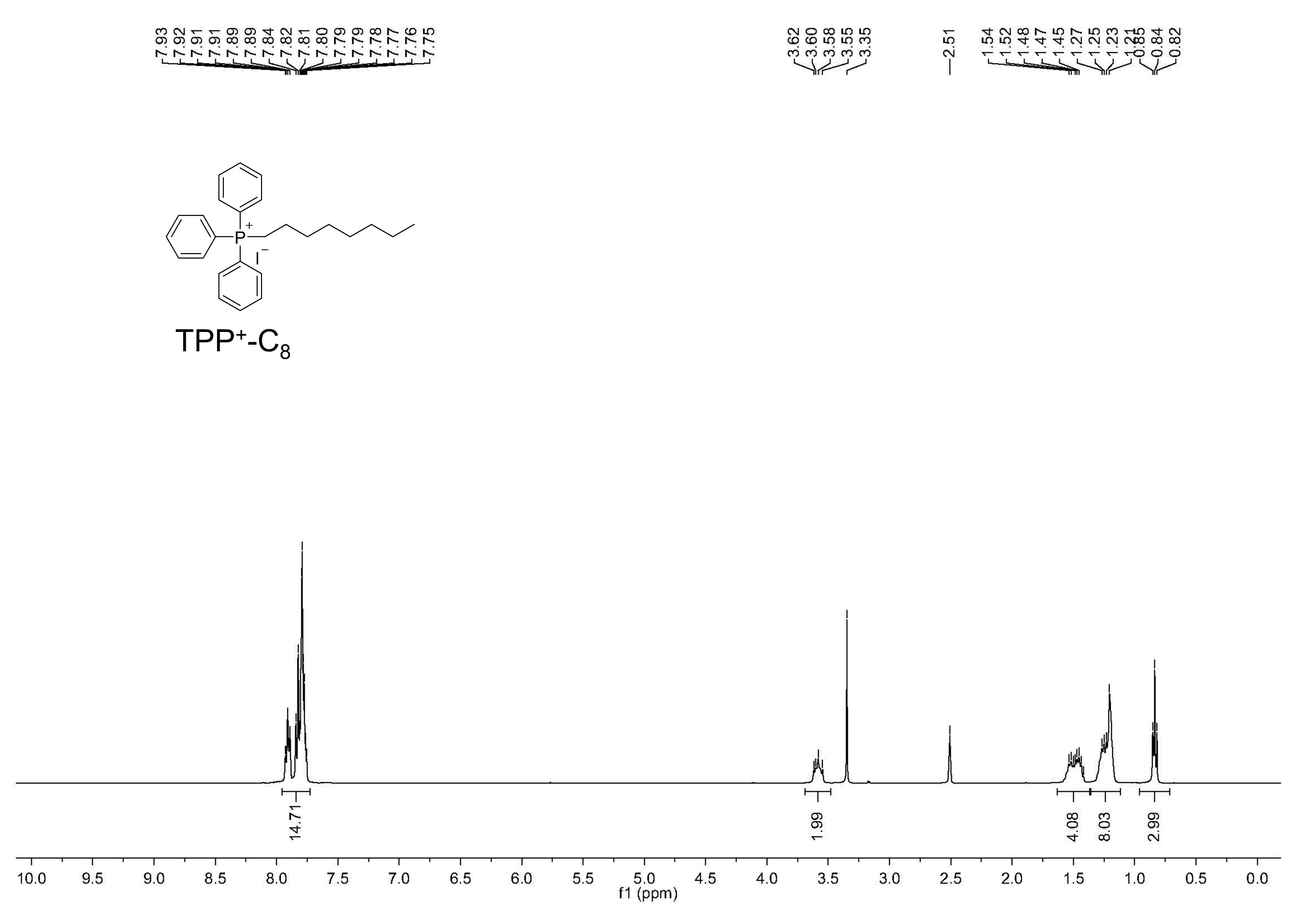


**Figure S43.** ^1^HNMR spectrum of TPP^+^-C_8_.


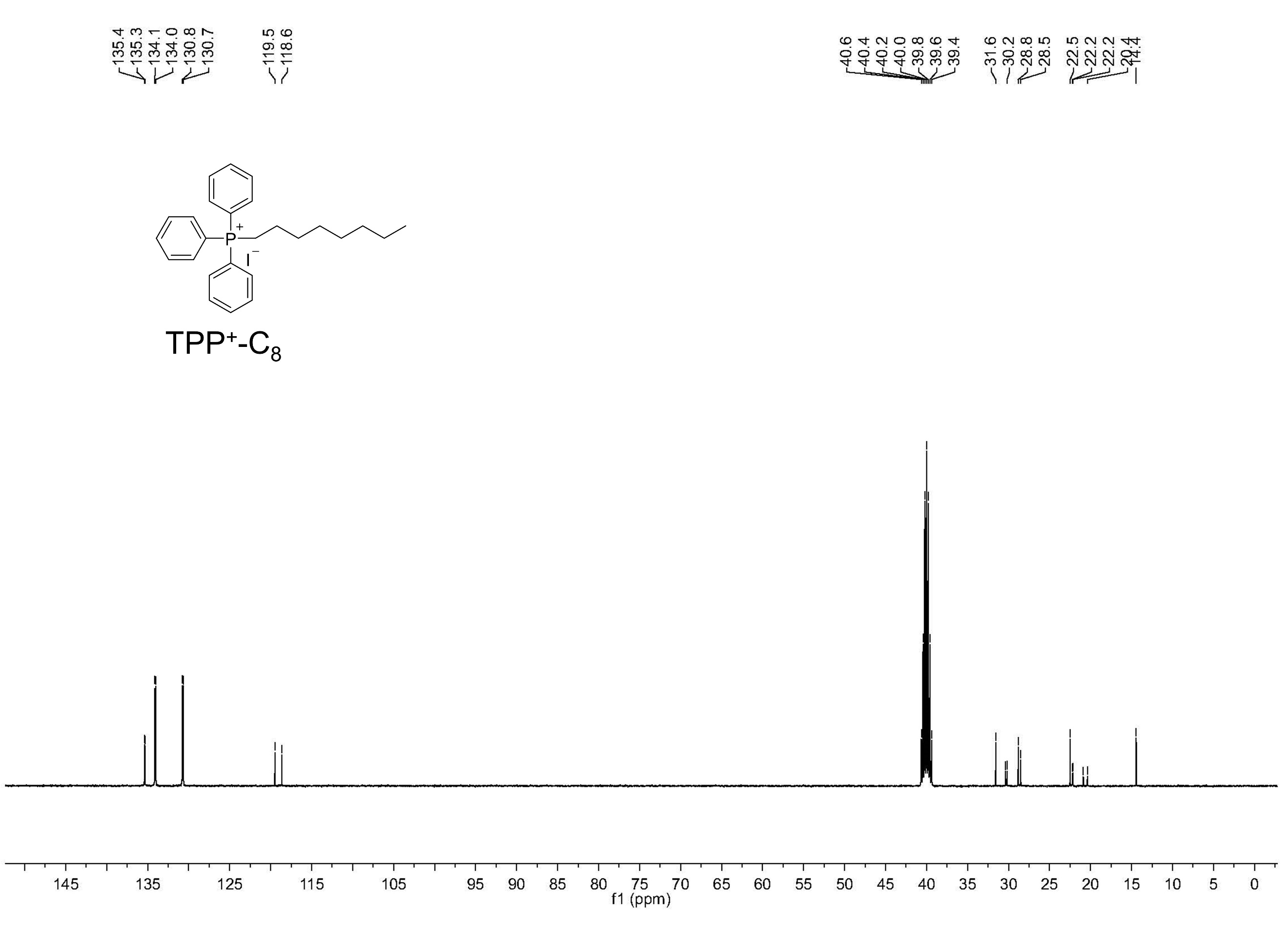


**Figure S44.** ^13^CNMR spectrum of TPP^+^-C_8_.


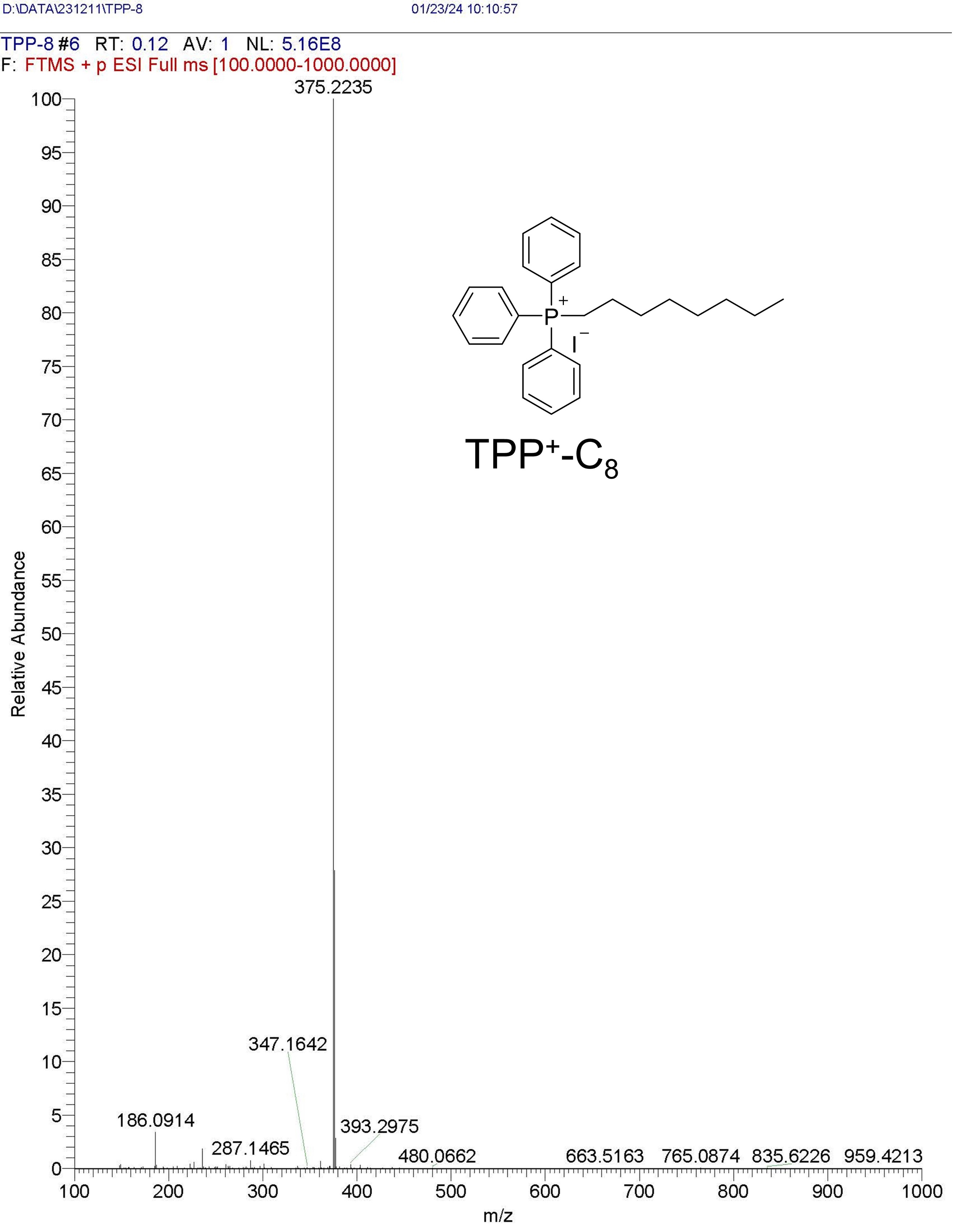


**Figure S45.** HR-MS spectrum of TPP^+^-C_8_.


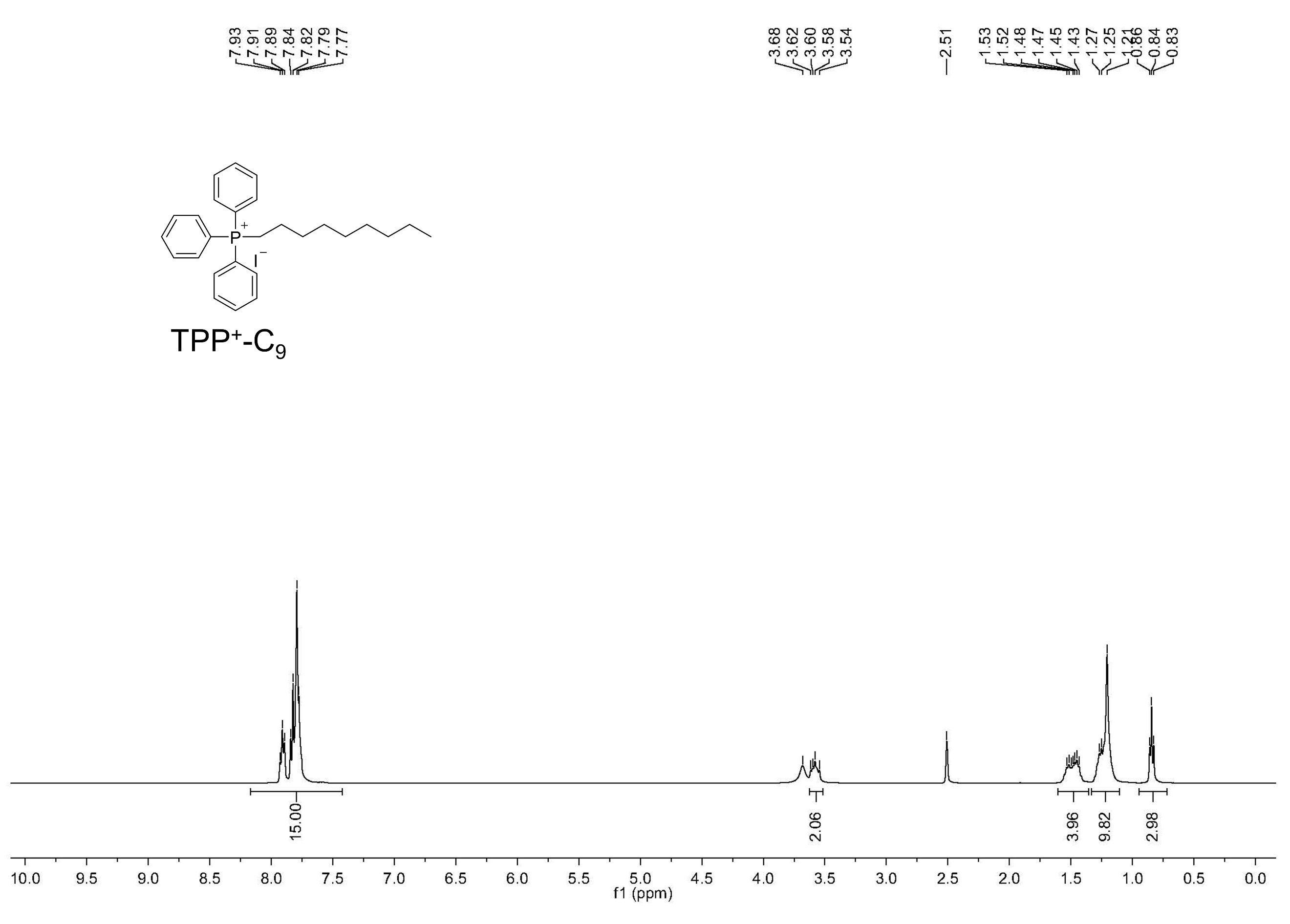


**Figure S46.** ^1^HNMR spectrum of TPP^+^-C_9_.


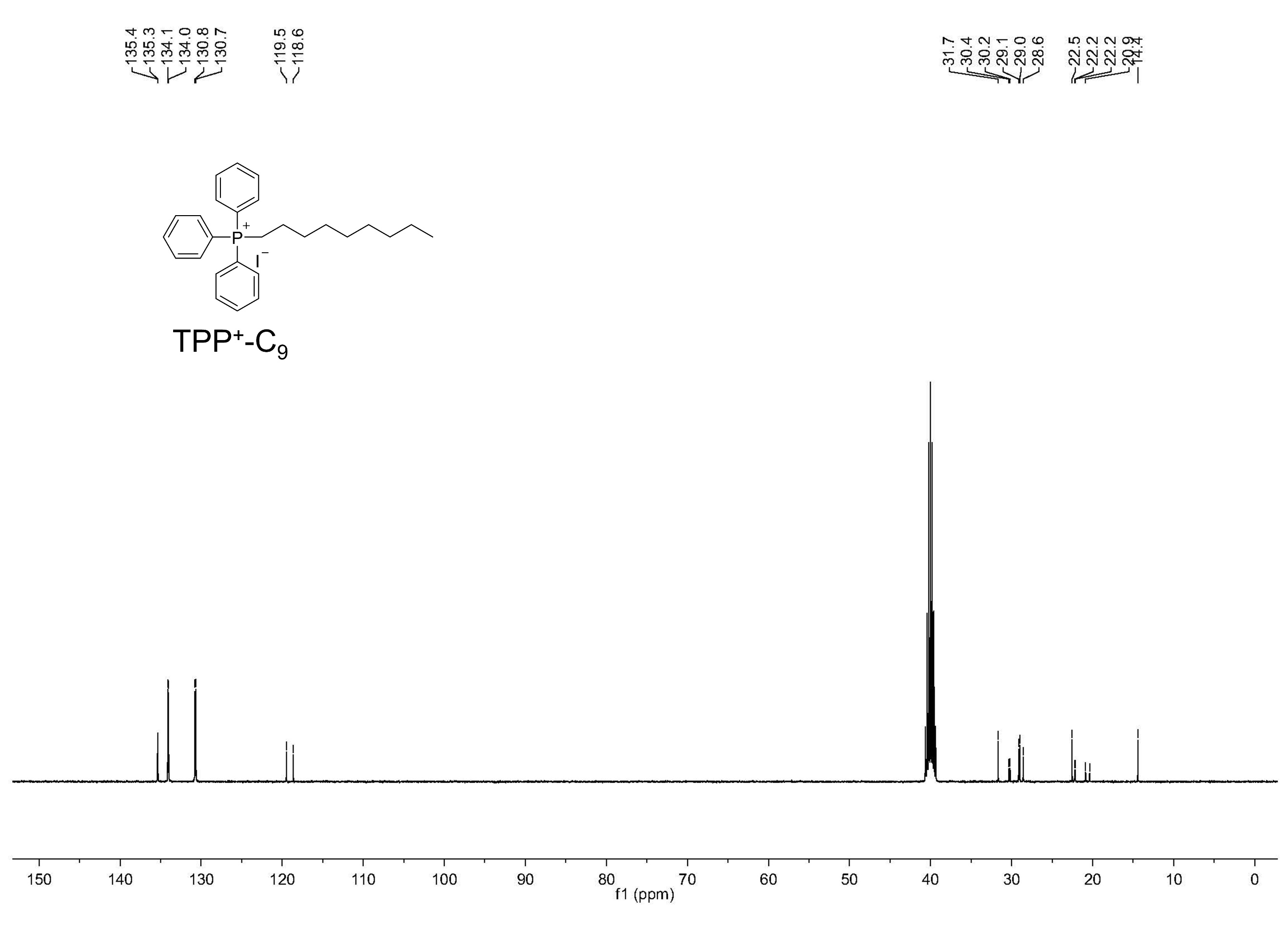


**Figure S47.** ^13^CNMR spectrum of TPP^+^-C_9_.


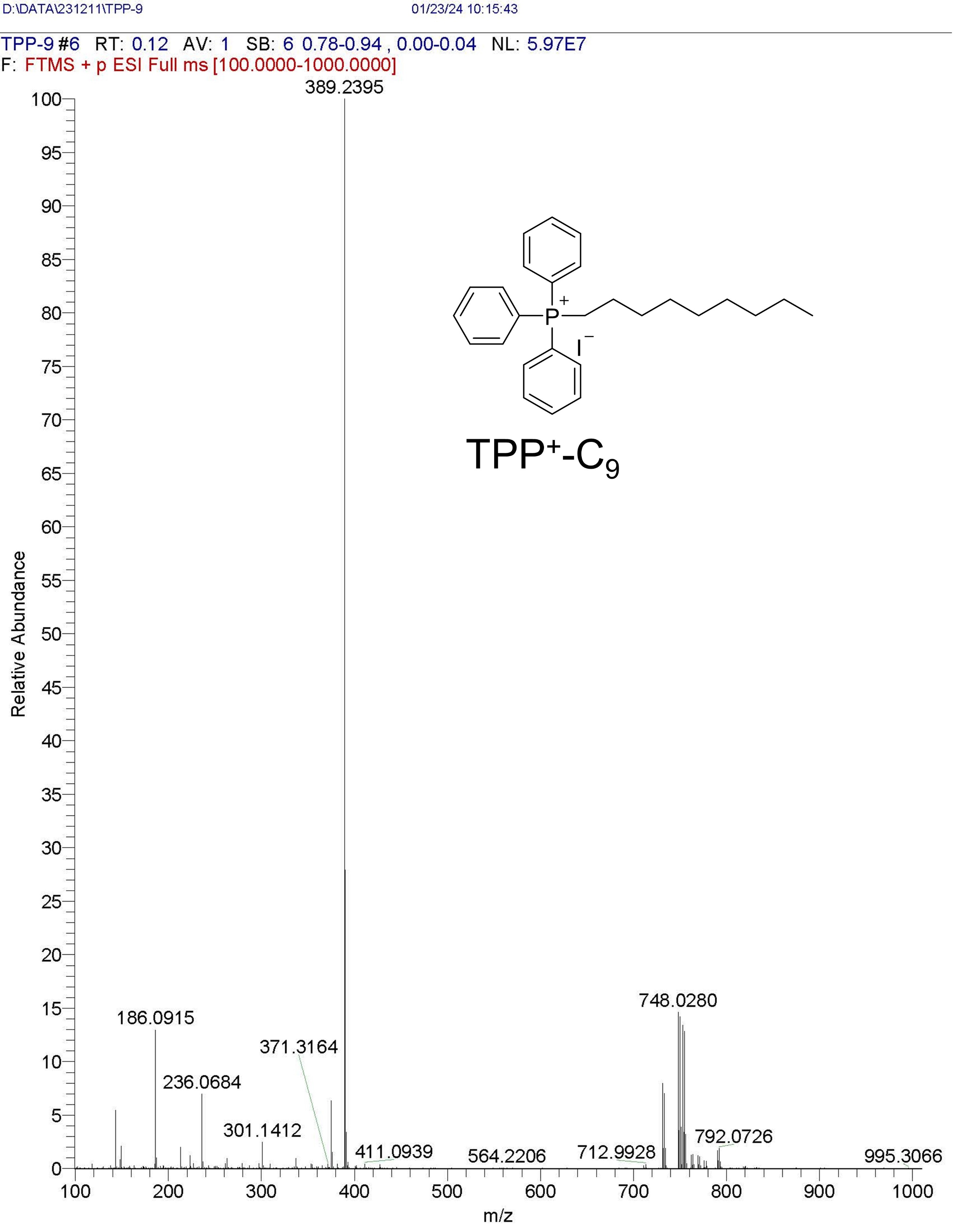


**Figure S48.** HR-MS spectrum of TPP^+^-C_9_.


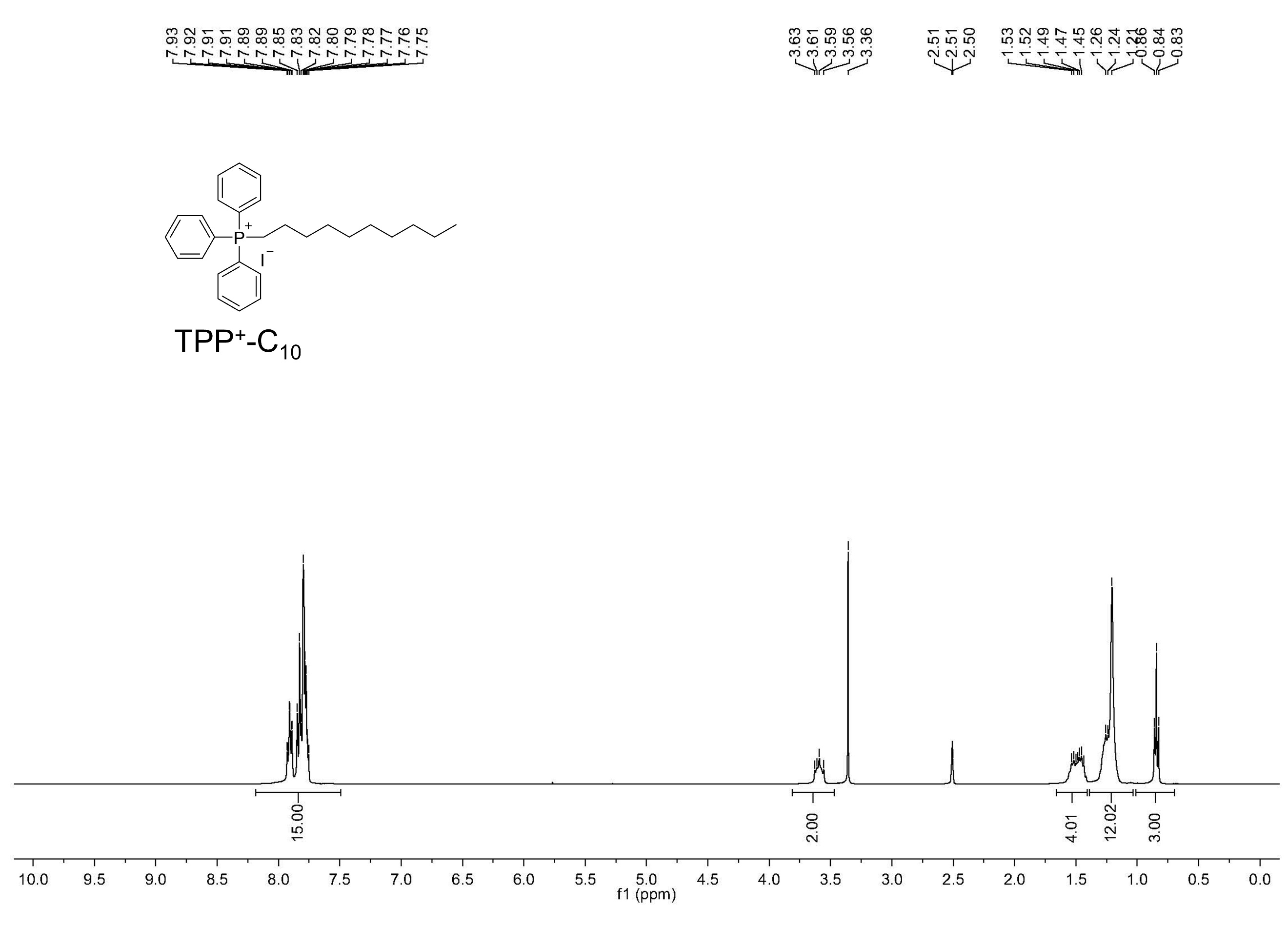


**Figure S49.** ^1^HNMR spectrum of TPP^+^-C_10_.


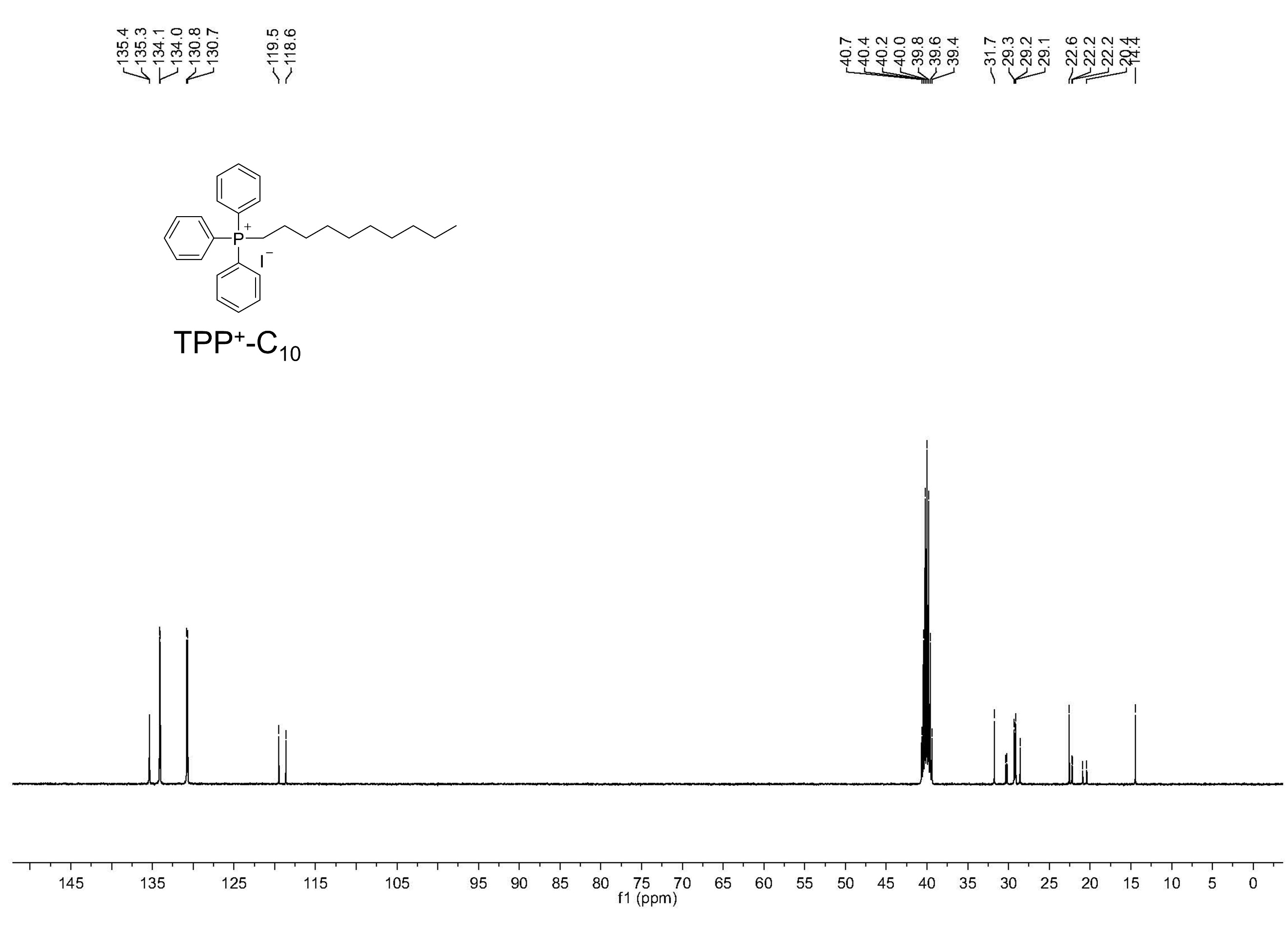


**Figure S50.** ^13^CNMR spectrum of TPP^+^-C_10_.


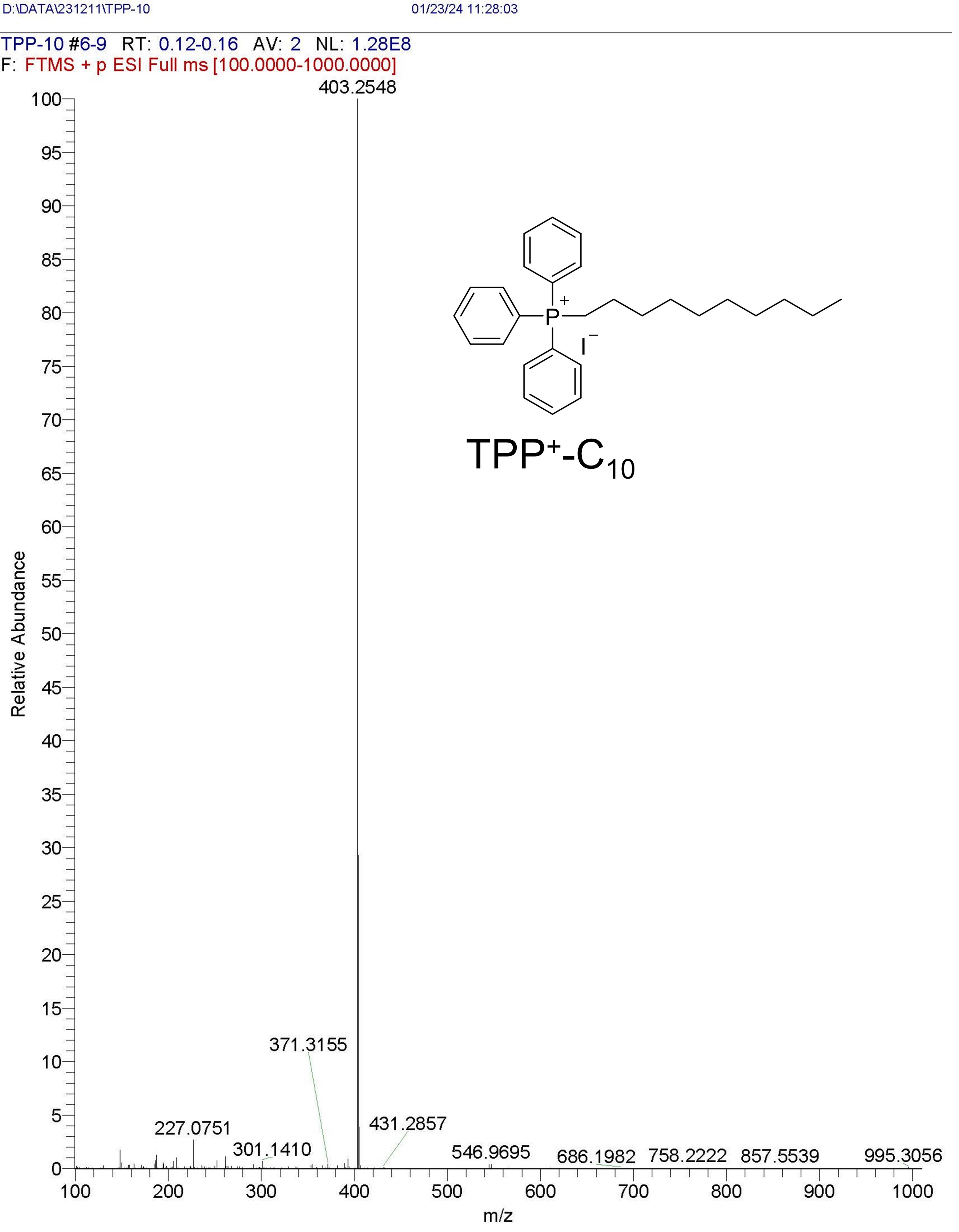


**Figure S51.** HR-MS spectrum of TPP^+^-C_10_.


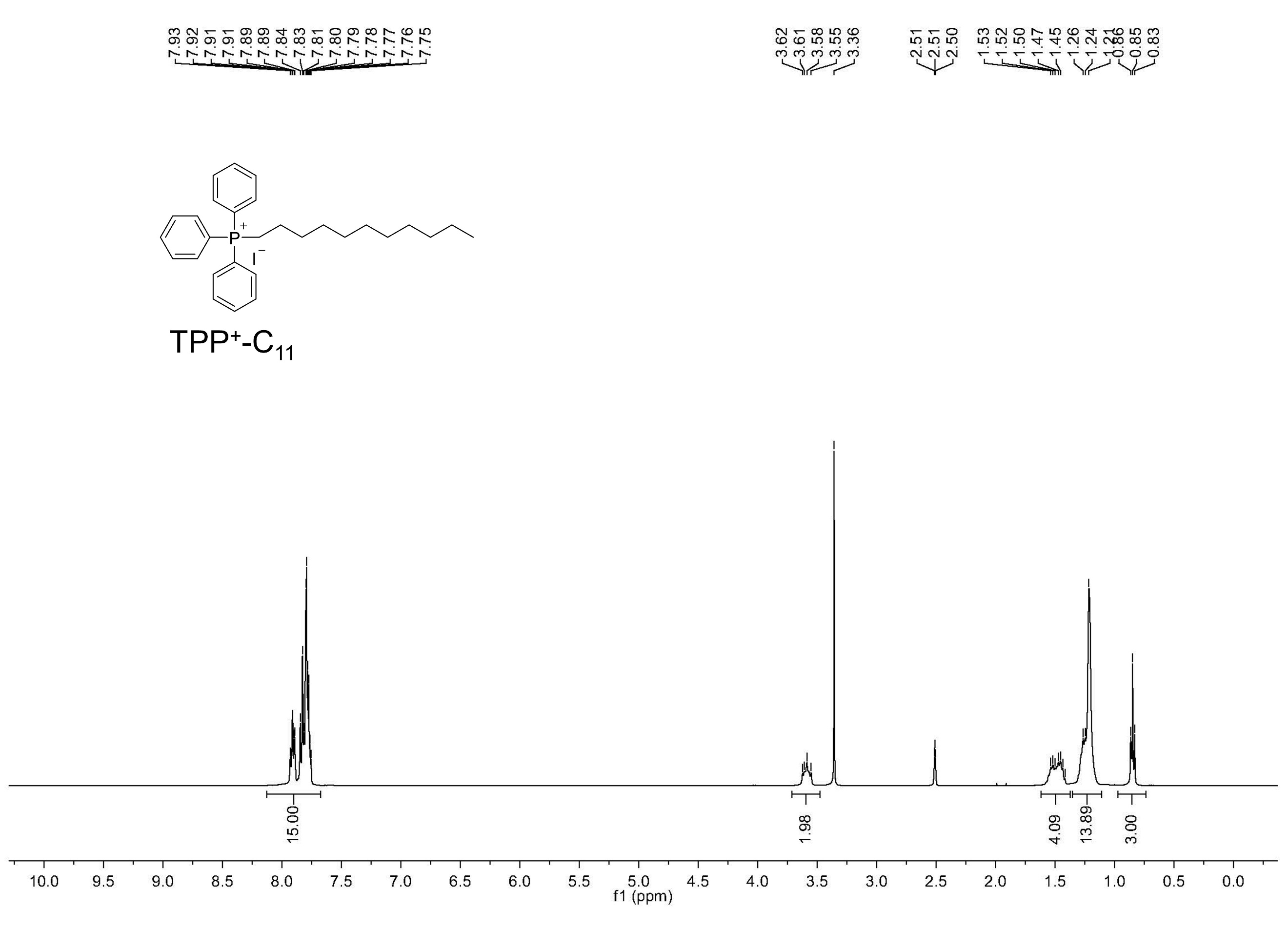


**Figure S52.** ^1^HNMR spectrum of TPP^+^-C_11_.

**
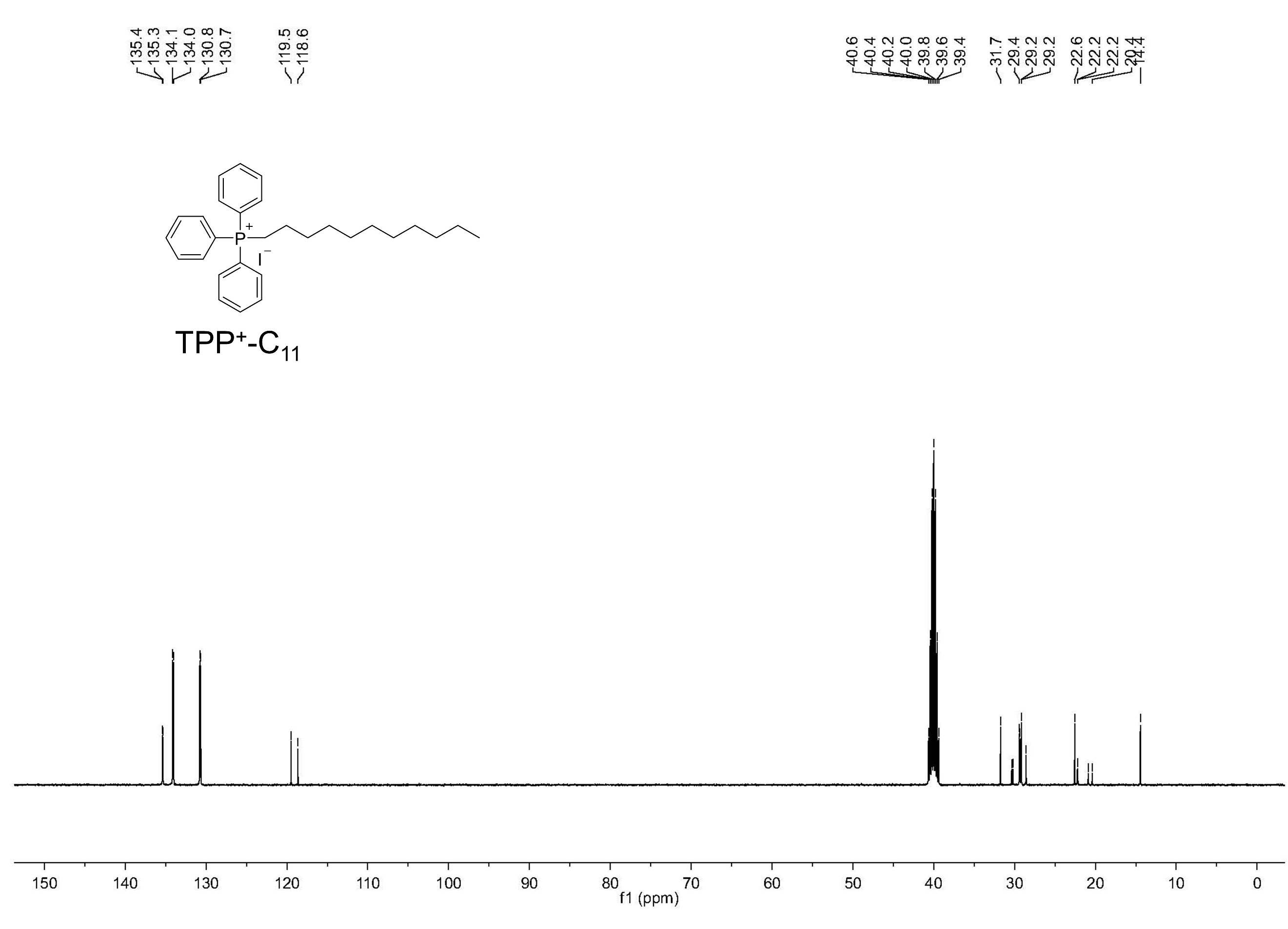
**

**Figure S53.** ^13^CNMR spectrum of TPP^+^-C_11_.


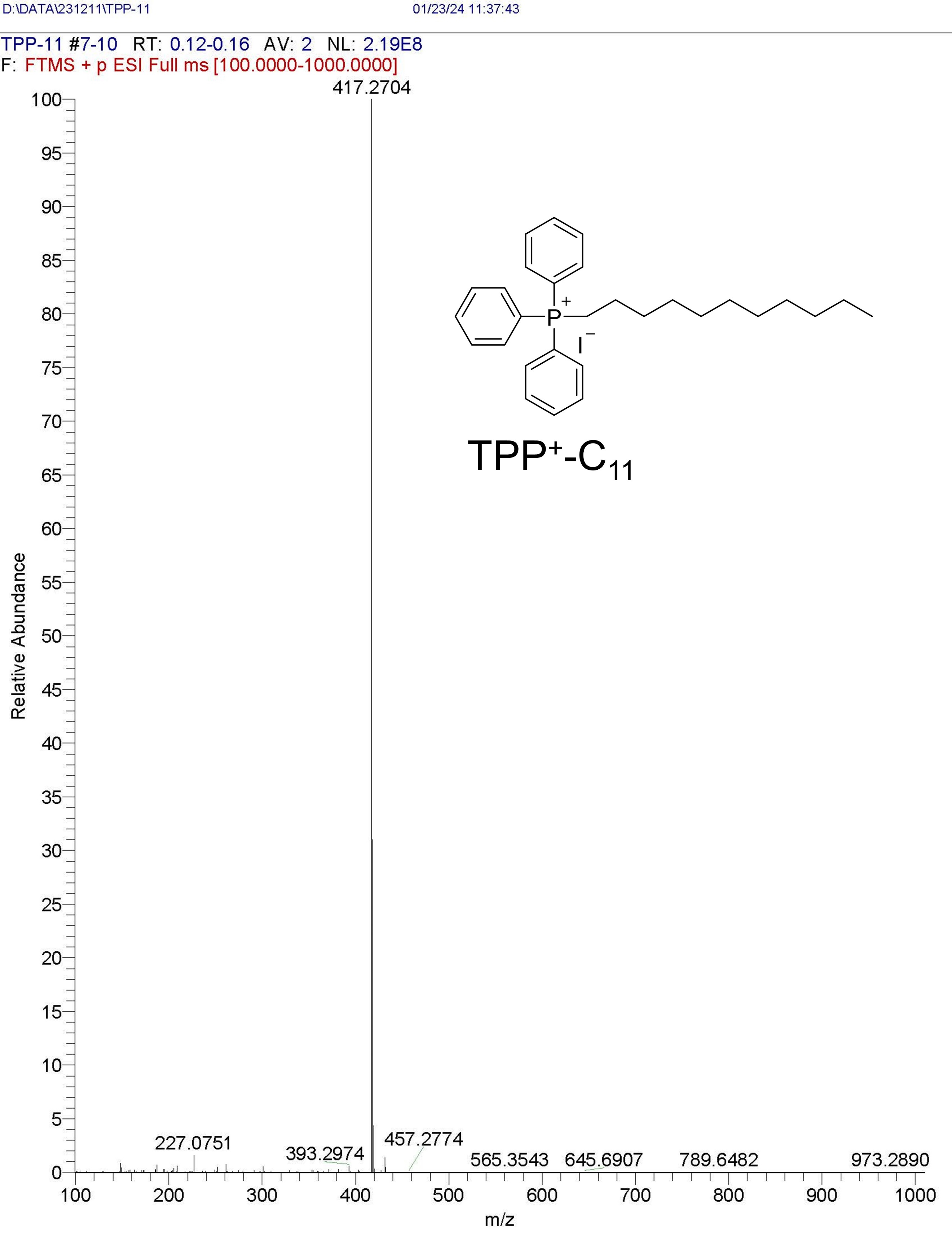


**Figure S54.** HR-MS spectrum of TPP^+^-C_11_.

**
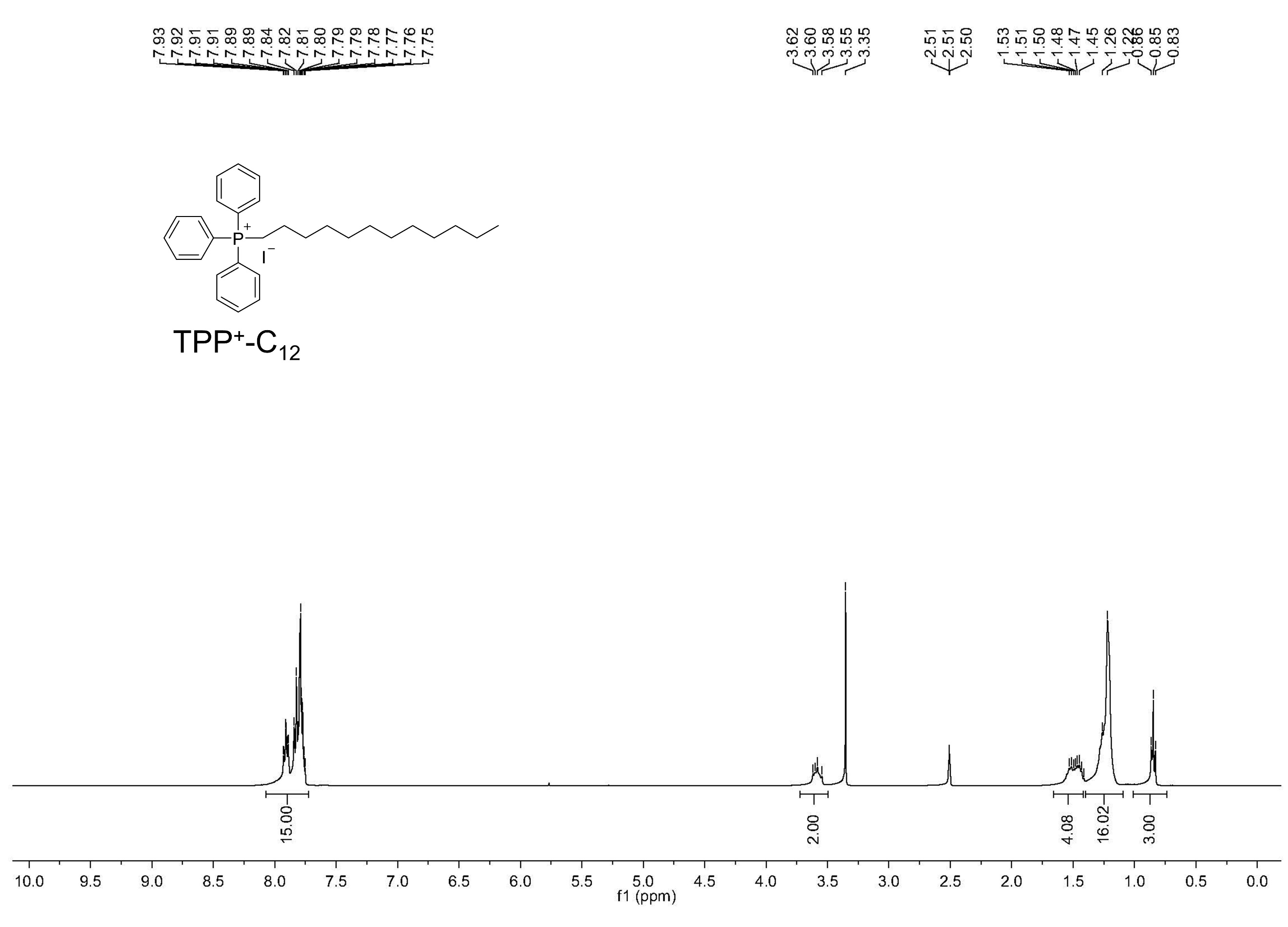
**

**Figure S55.** ^1^HNMR spectrum of TPP^+^-C_12_.


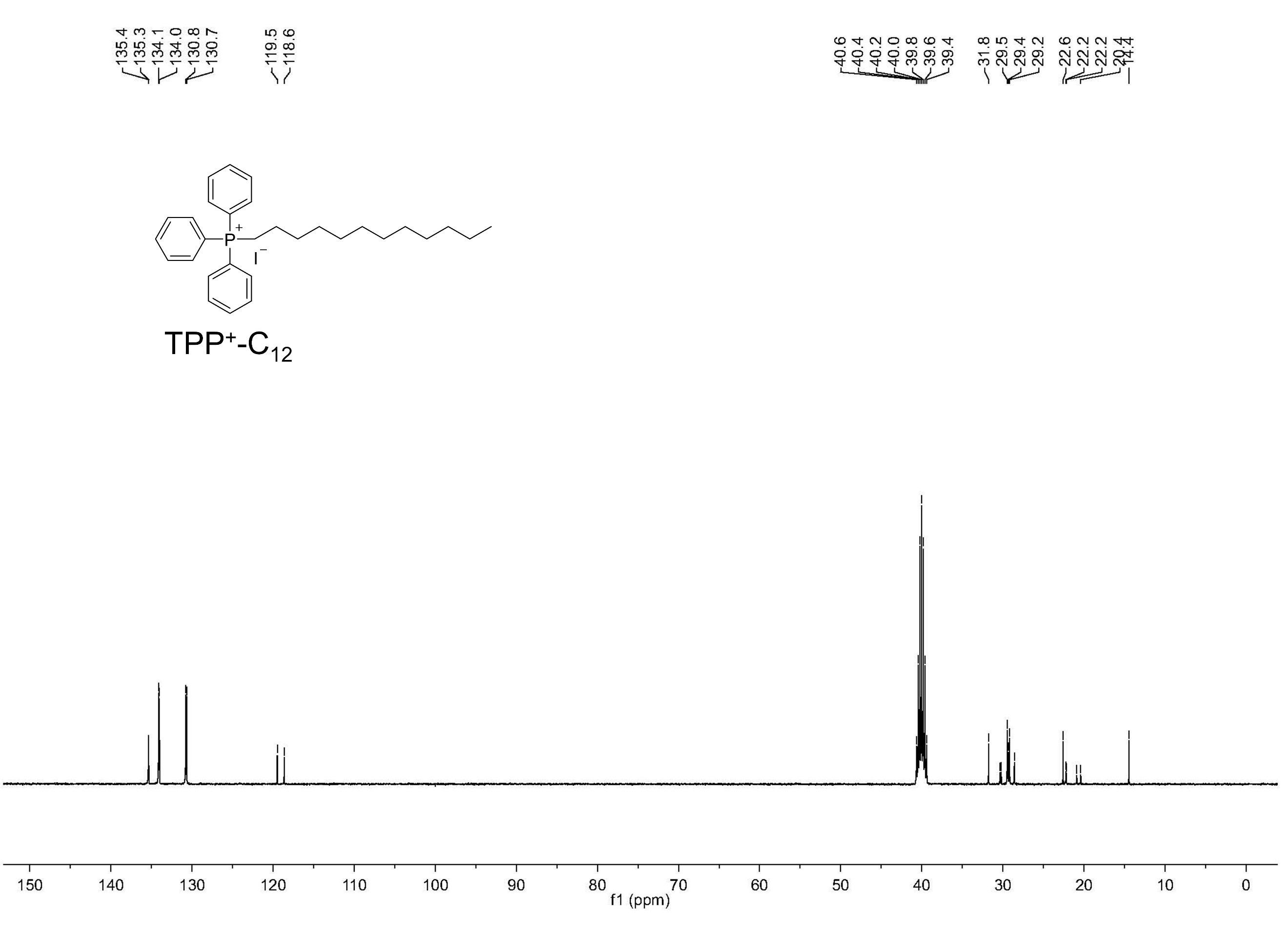


**Figure S56.** ^13^CNMR spectrum of TPP^+^-C_12_.


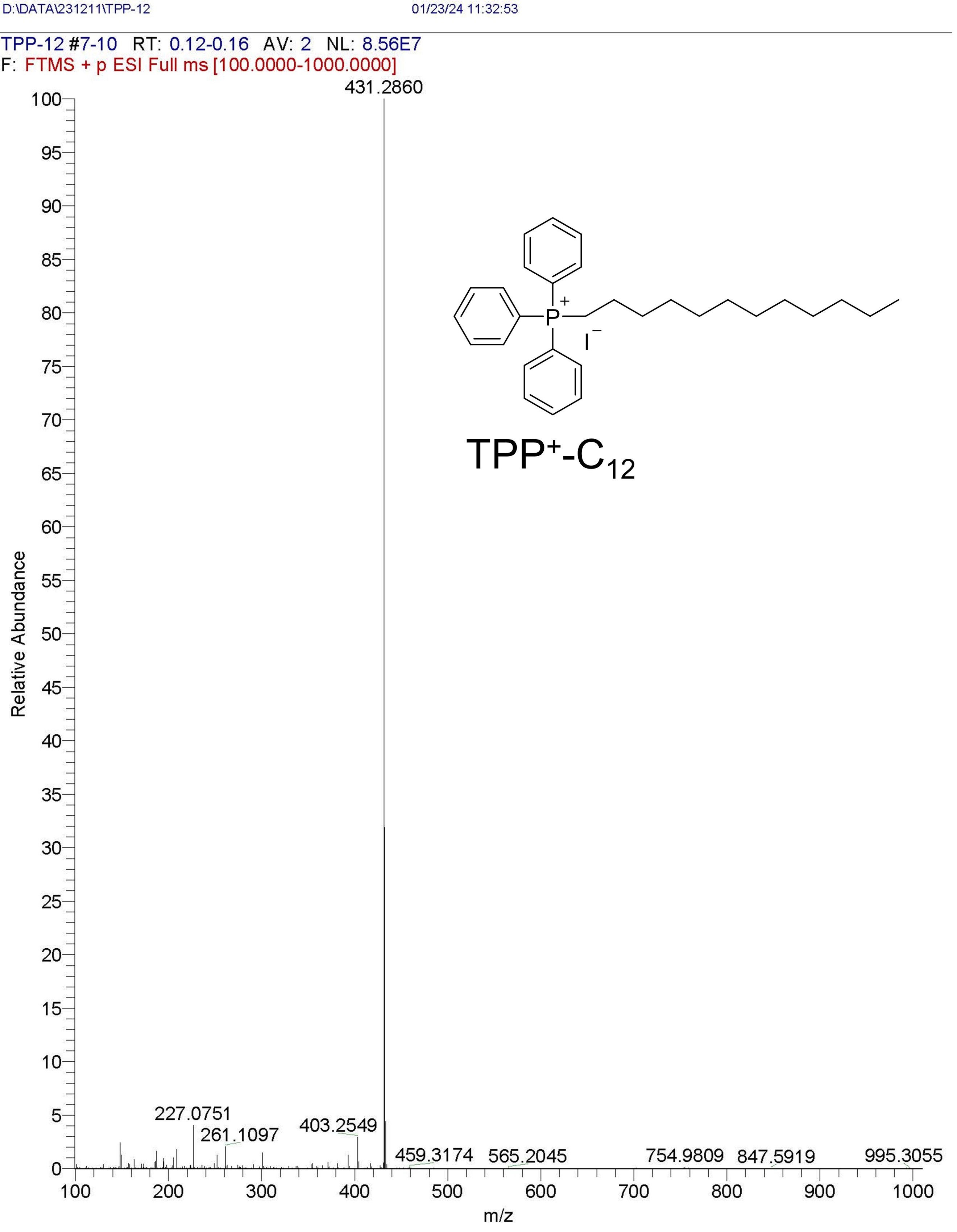


**Figure S57.** HR-MS spectrum of TPP^+^-C_12_.


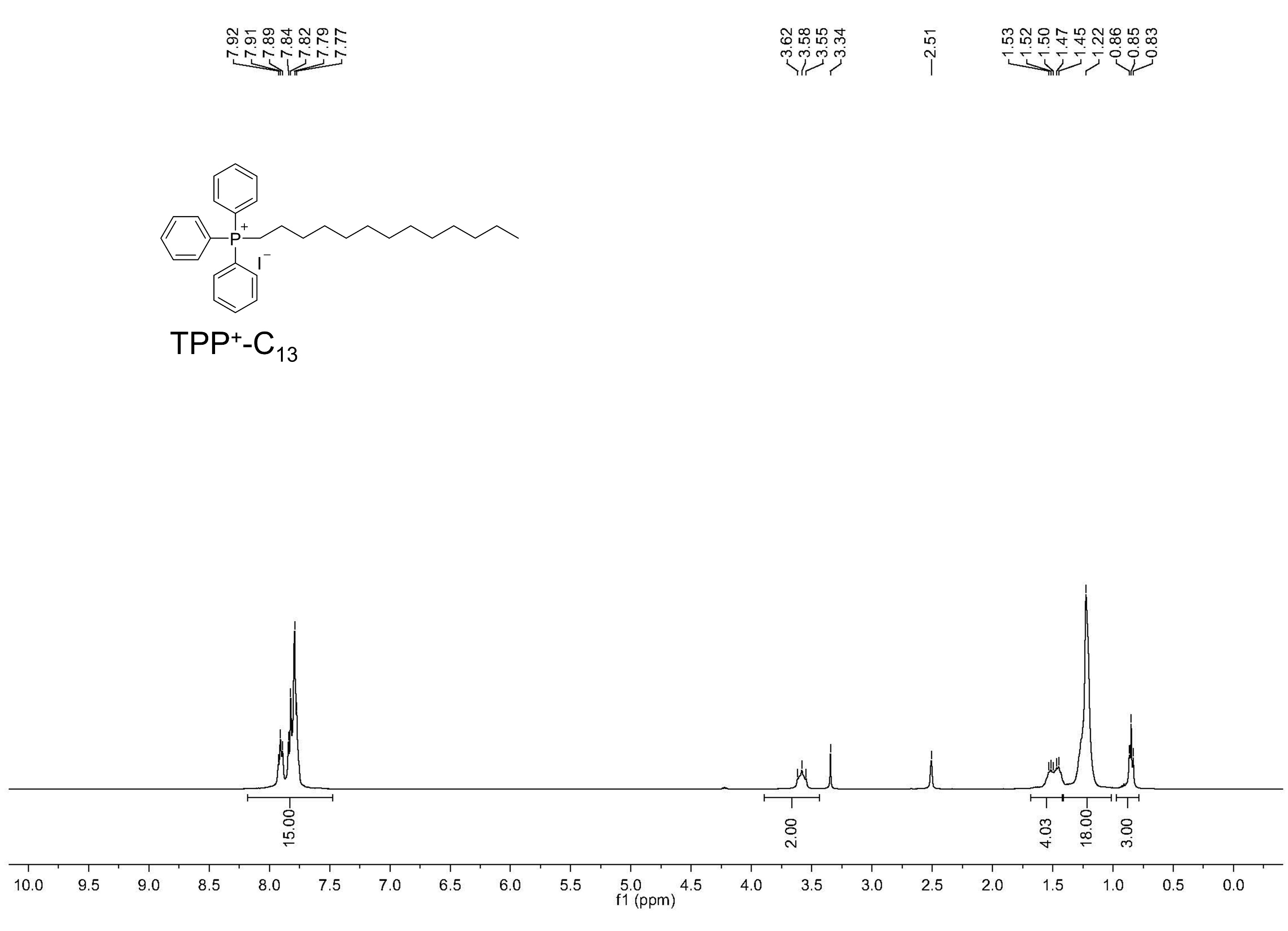


**Figure S58.** ^1^HNMR spectrum of TPP^+^-C_13_.


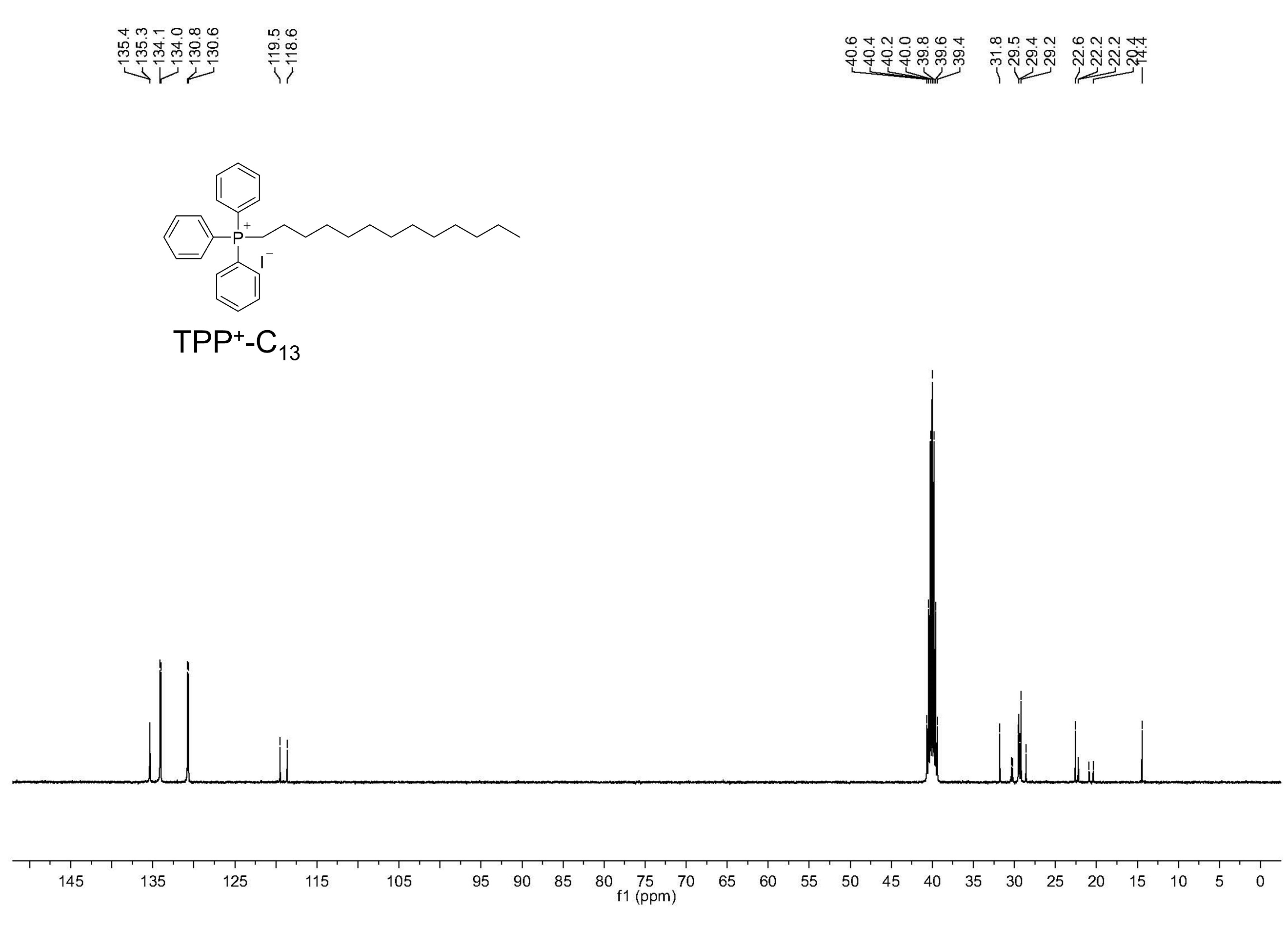


**Figure S59.** ^13^CNMR spectrum of TPP^+^-C_13_.


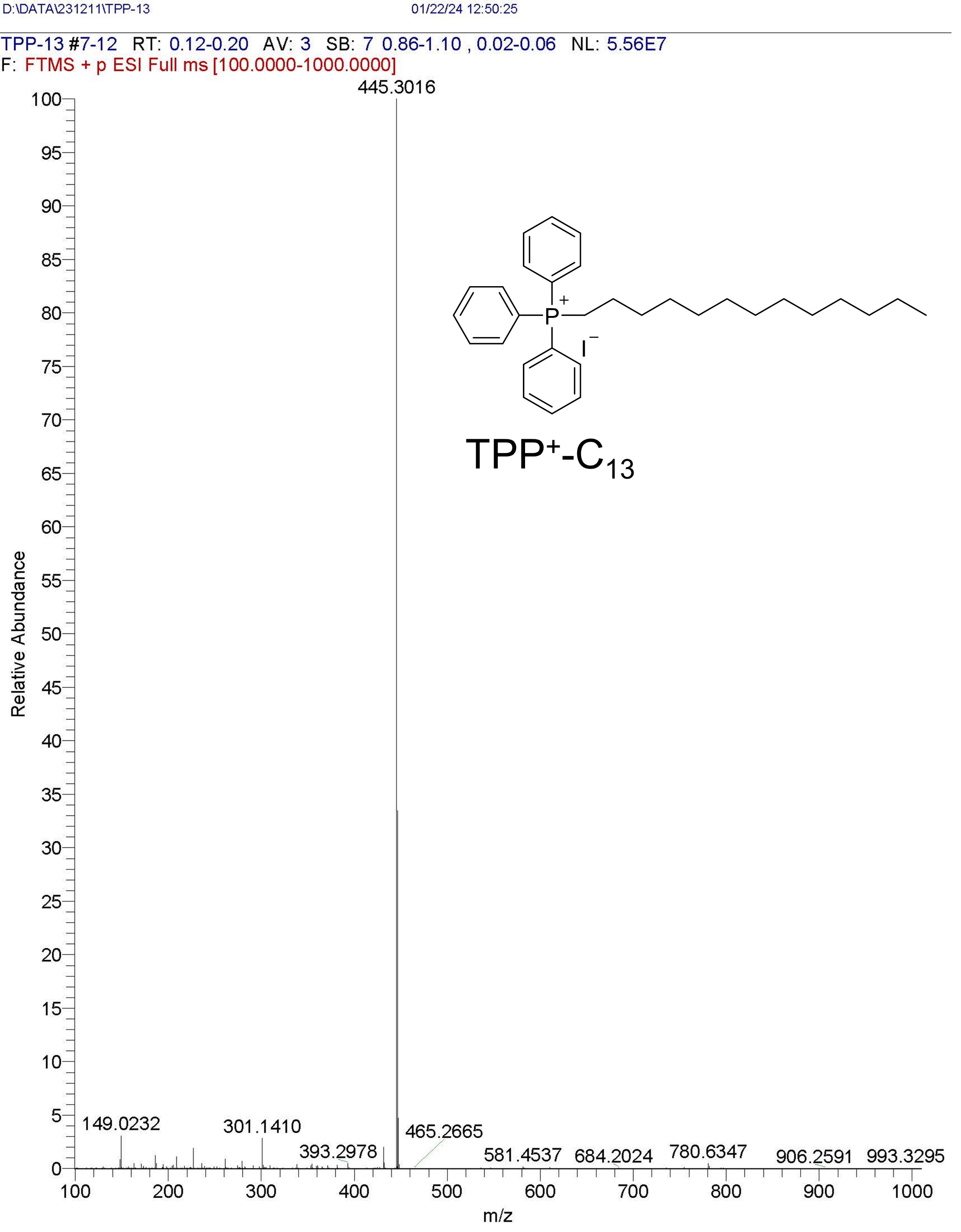


**Figure S60.** HR-MS spectrum of TPP^+^-C_13_.


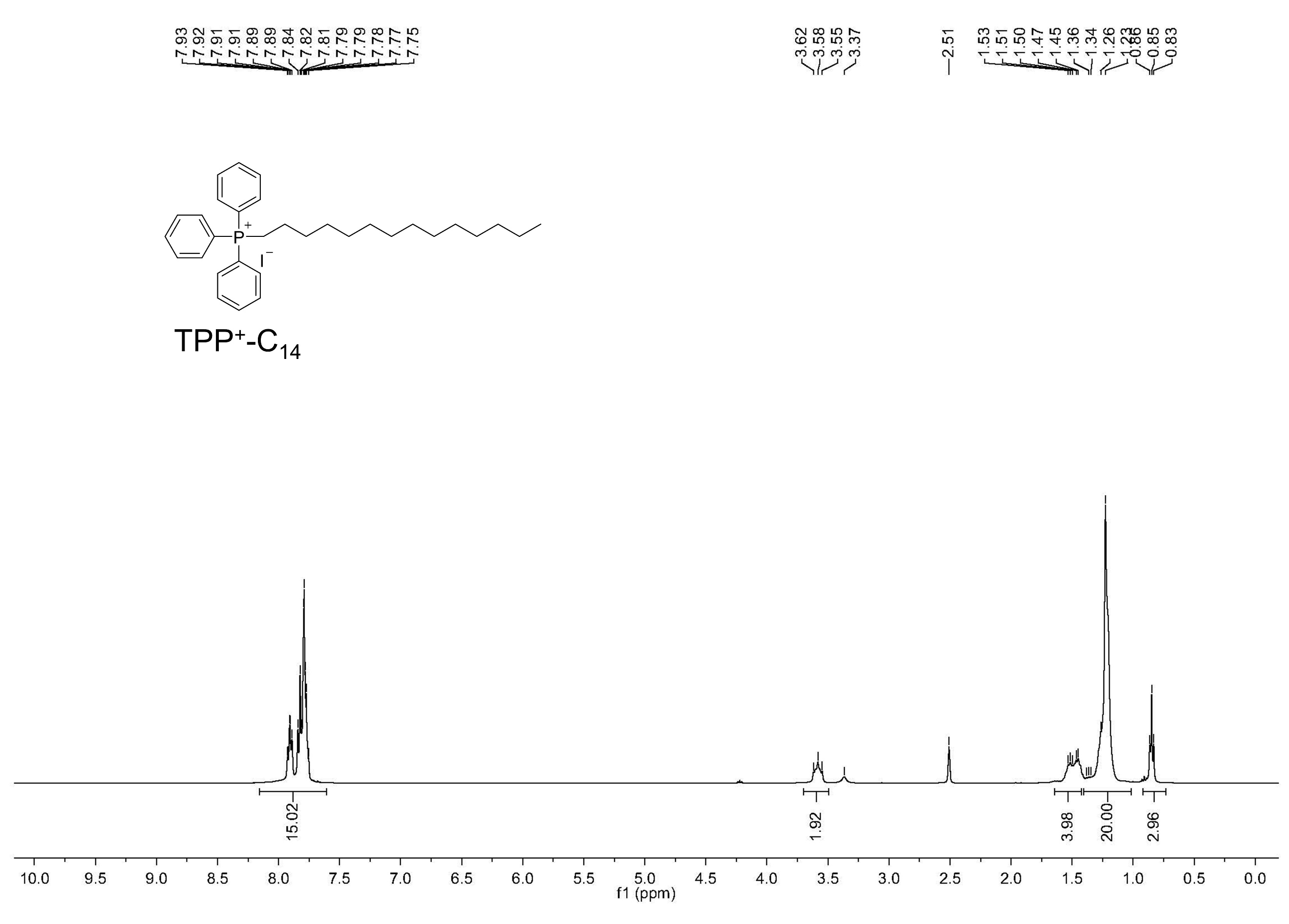


**Figure S61.** ^1^HNMR spectrum of TPP^+^-C_14_.


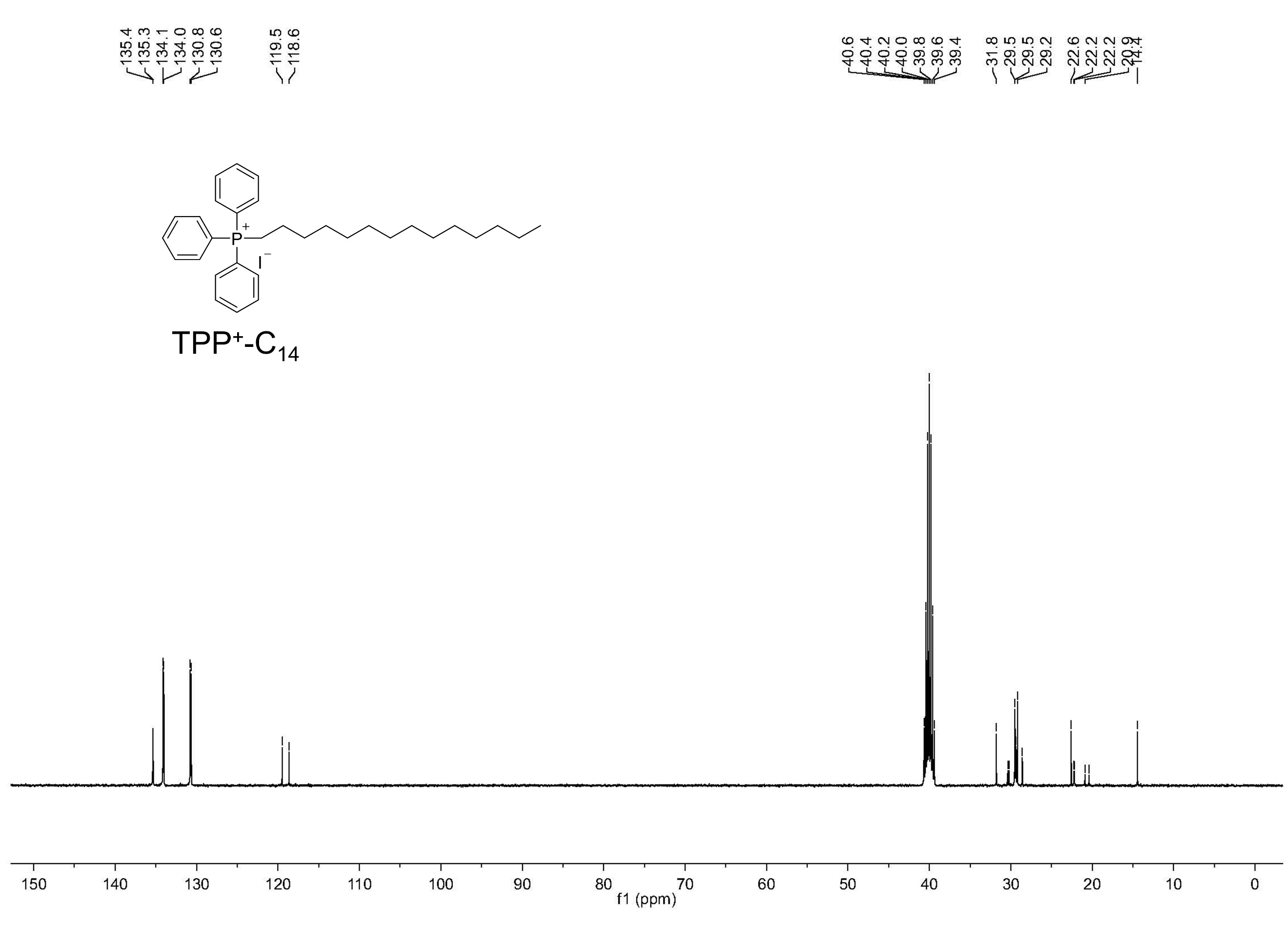


**Figure S62.** ^13^CNMR spectrum of TPP^+^-C_14_.


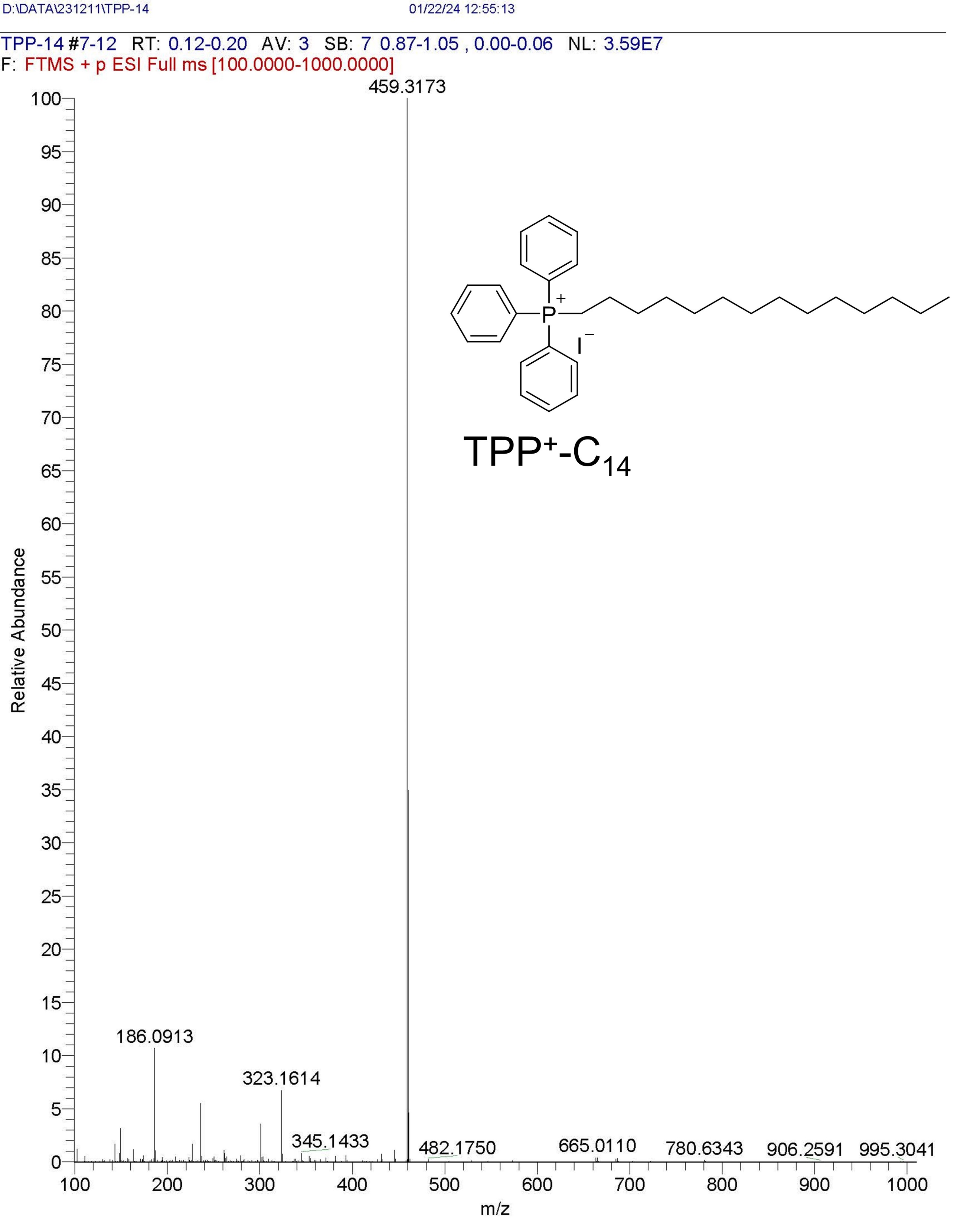


**Figure S63.** HR-MS spectrum of TPP^+^-C_14_.


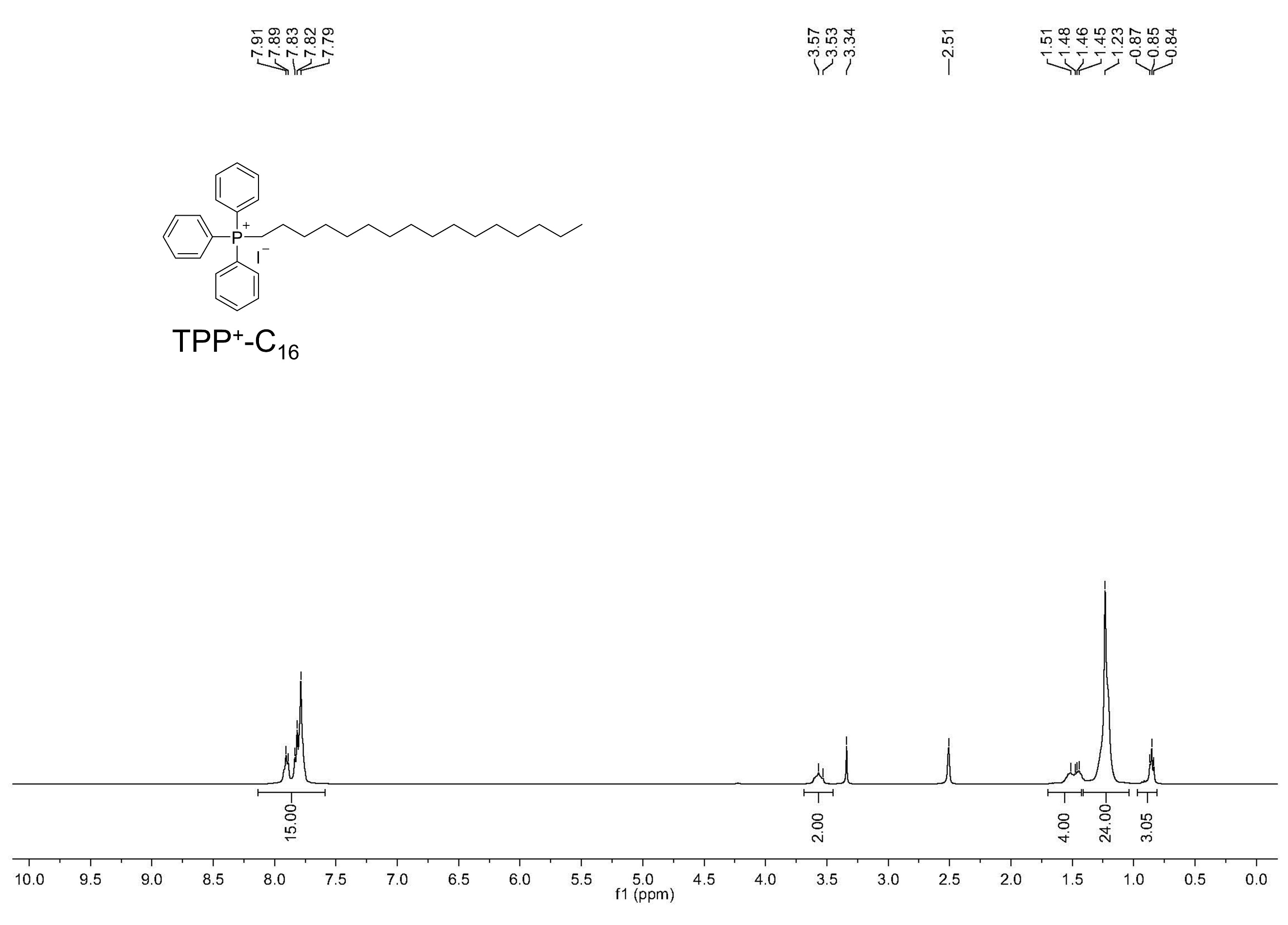


**Figure S64.** ^1^HNMR spectrum of TPP^+^-C_16_.


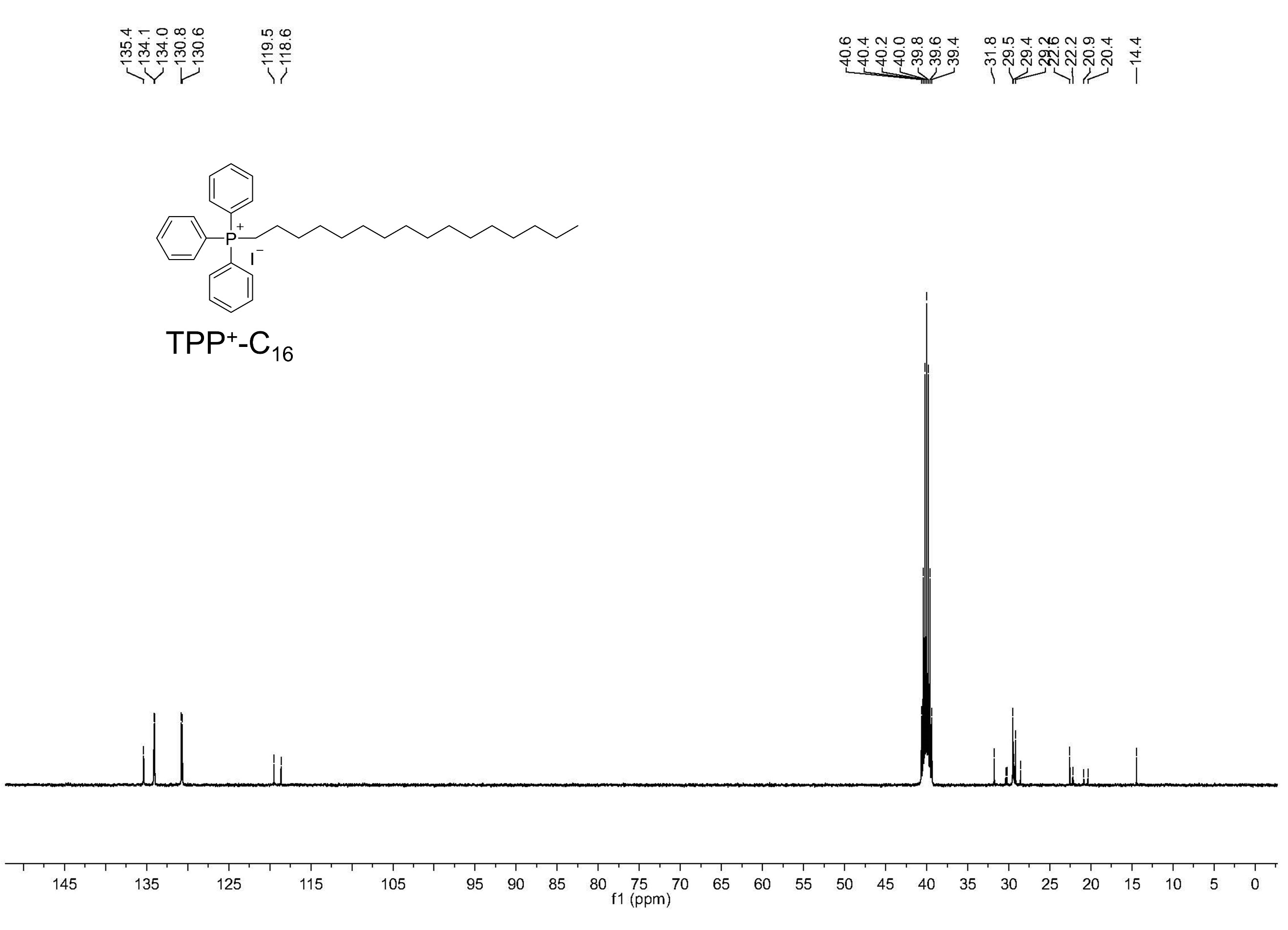


**Figure S65.** ^13^CNMR spectrum of TPP^+^-C_16_.


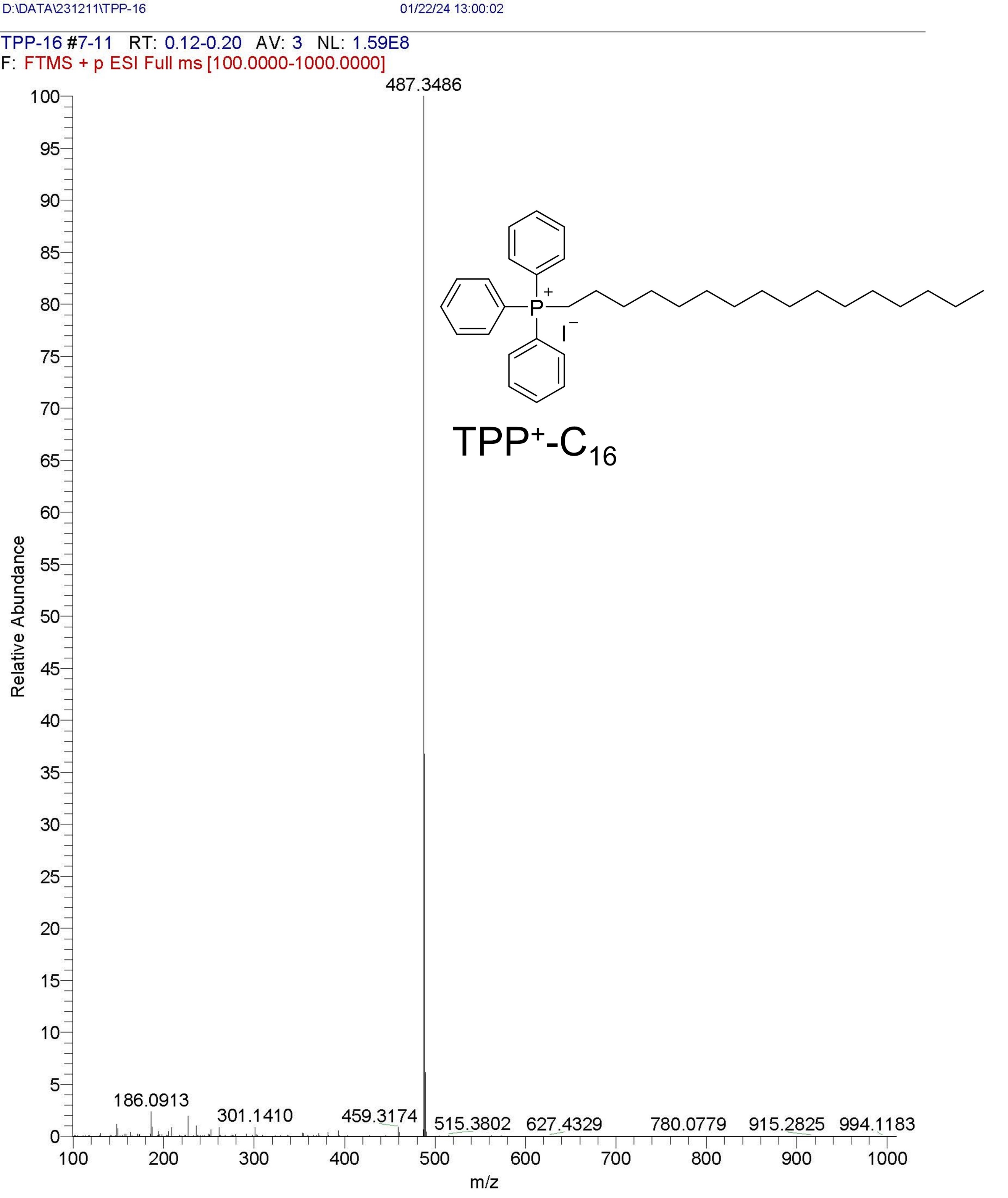


**Figure S66.** HR-MS spectrum of TPP^+^-C_16_.


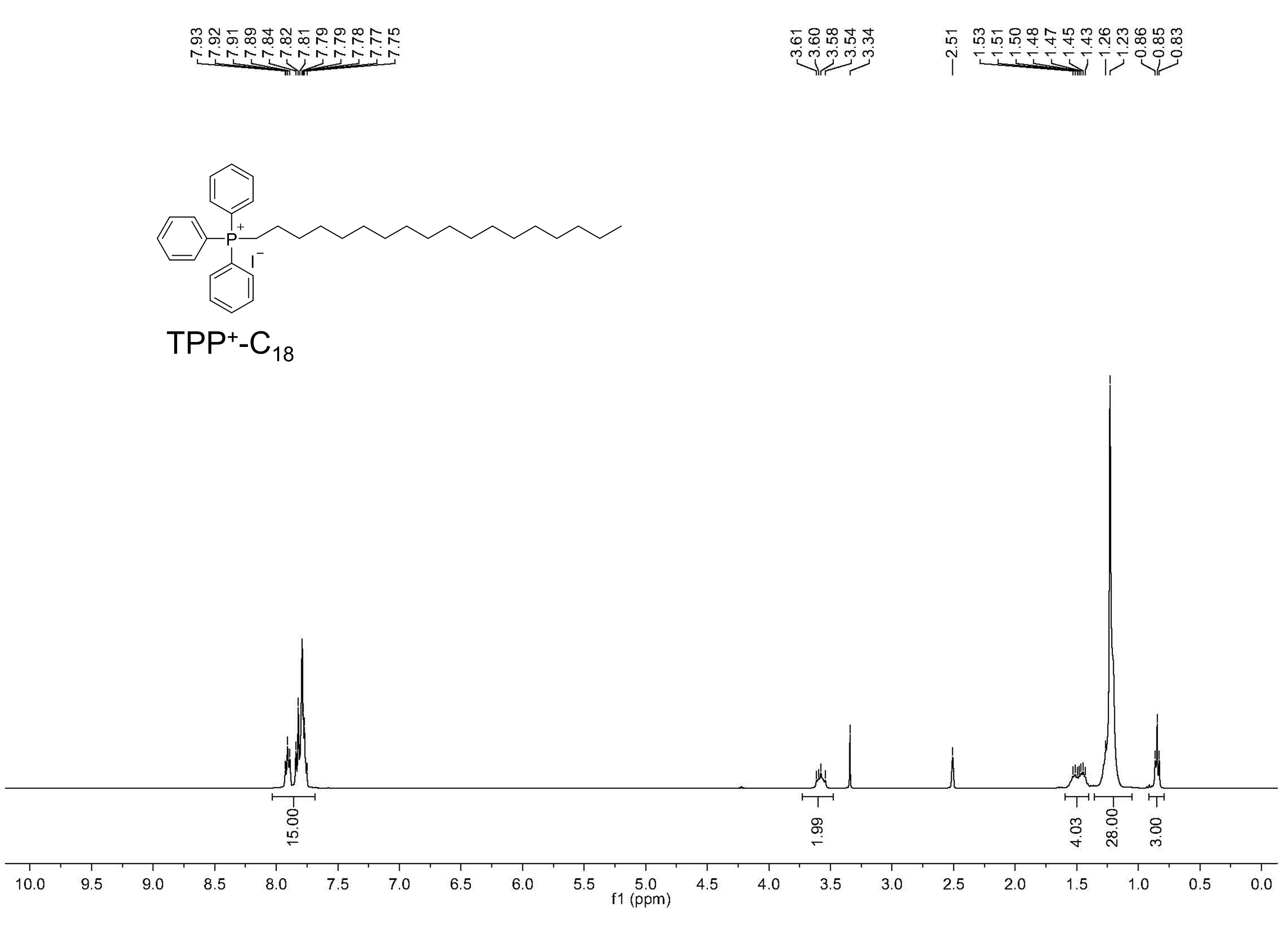


**Figure S67.** ^1^HNMR spectrum of TPP^+^-C_18_.


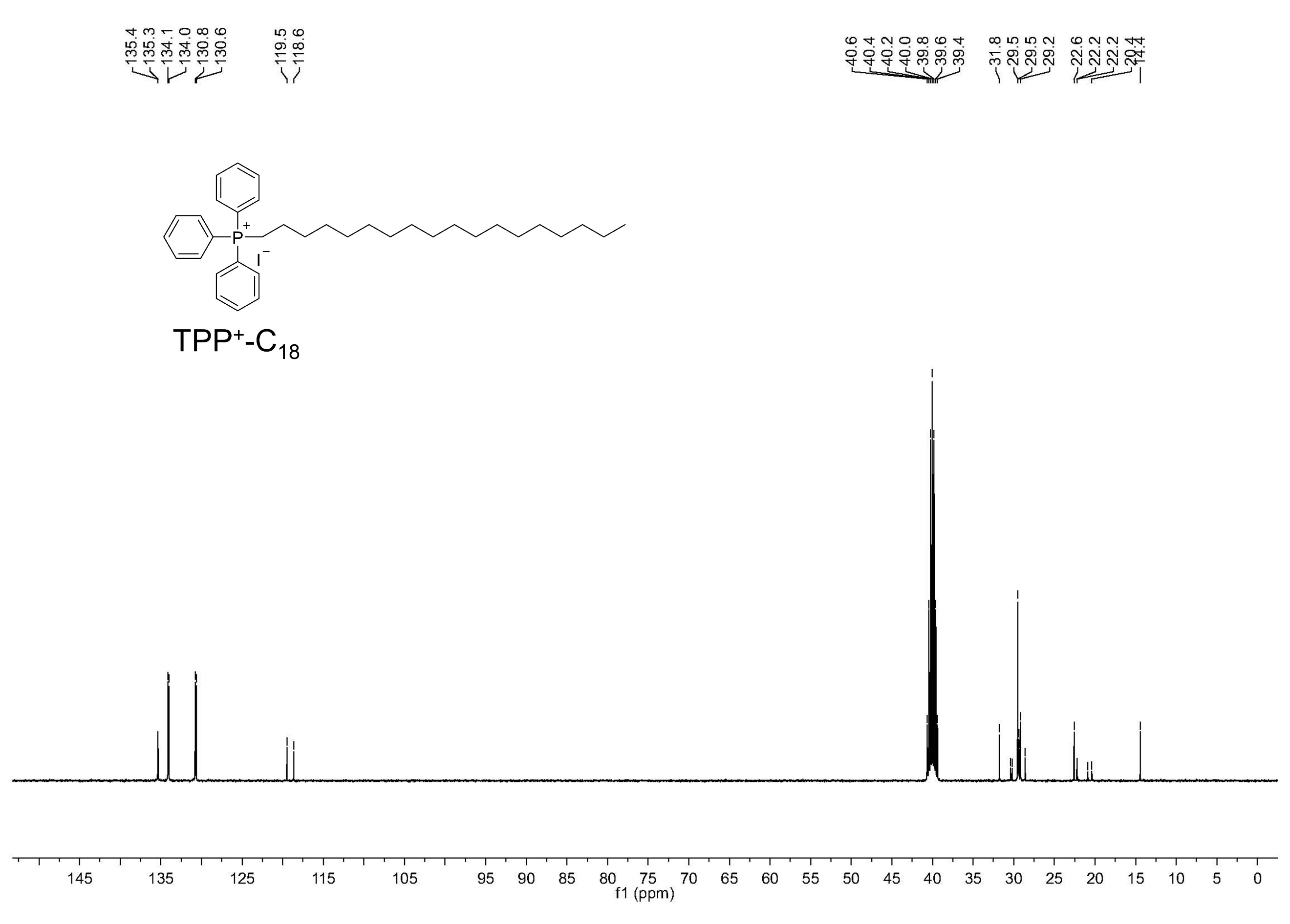


**Figure S68.** ^13^CNMR spectrum of TPP^+^-C_18_.


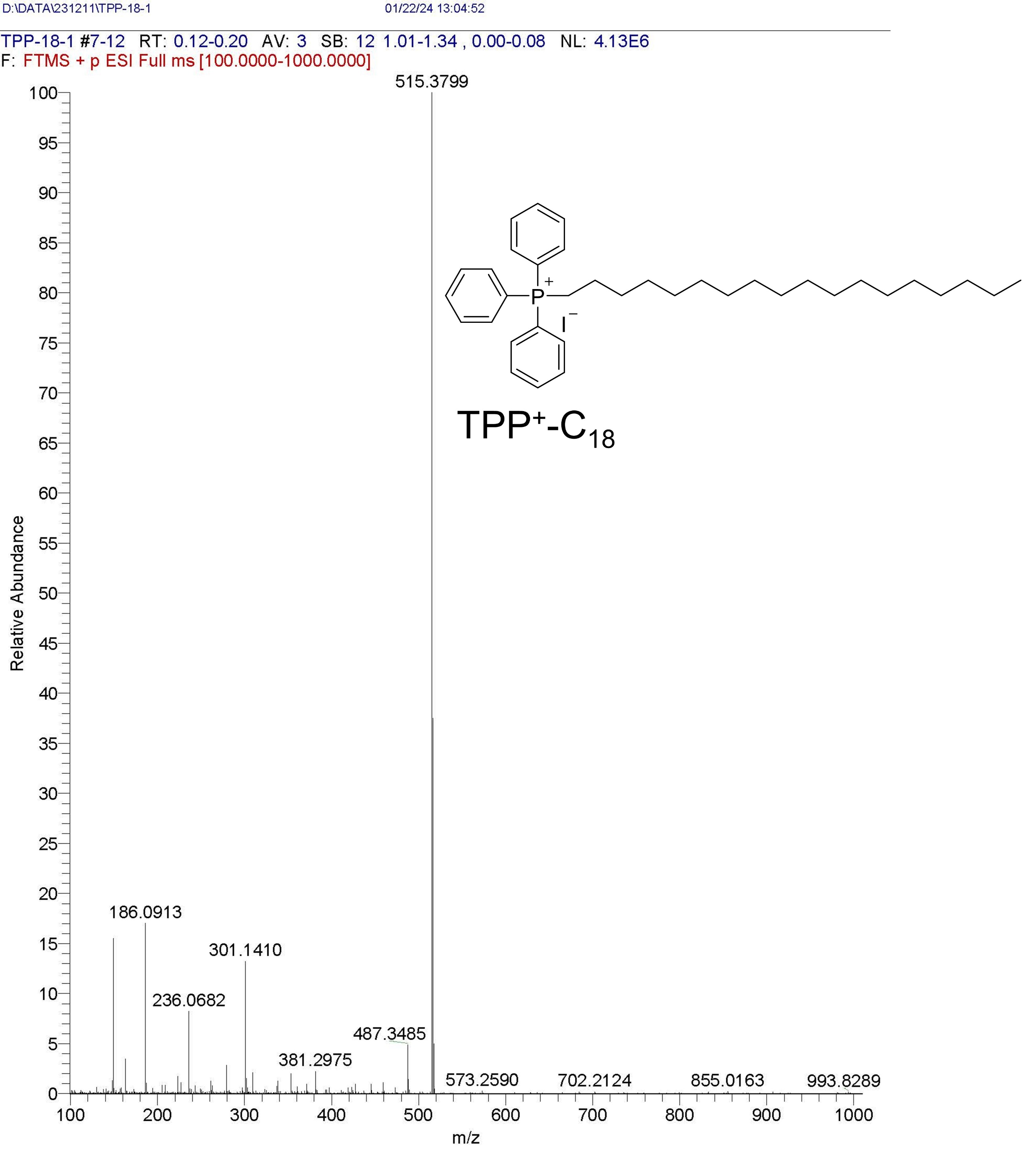


**Figure S69.** HR-MS spectrum of TPP^+^-C_18_.


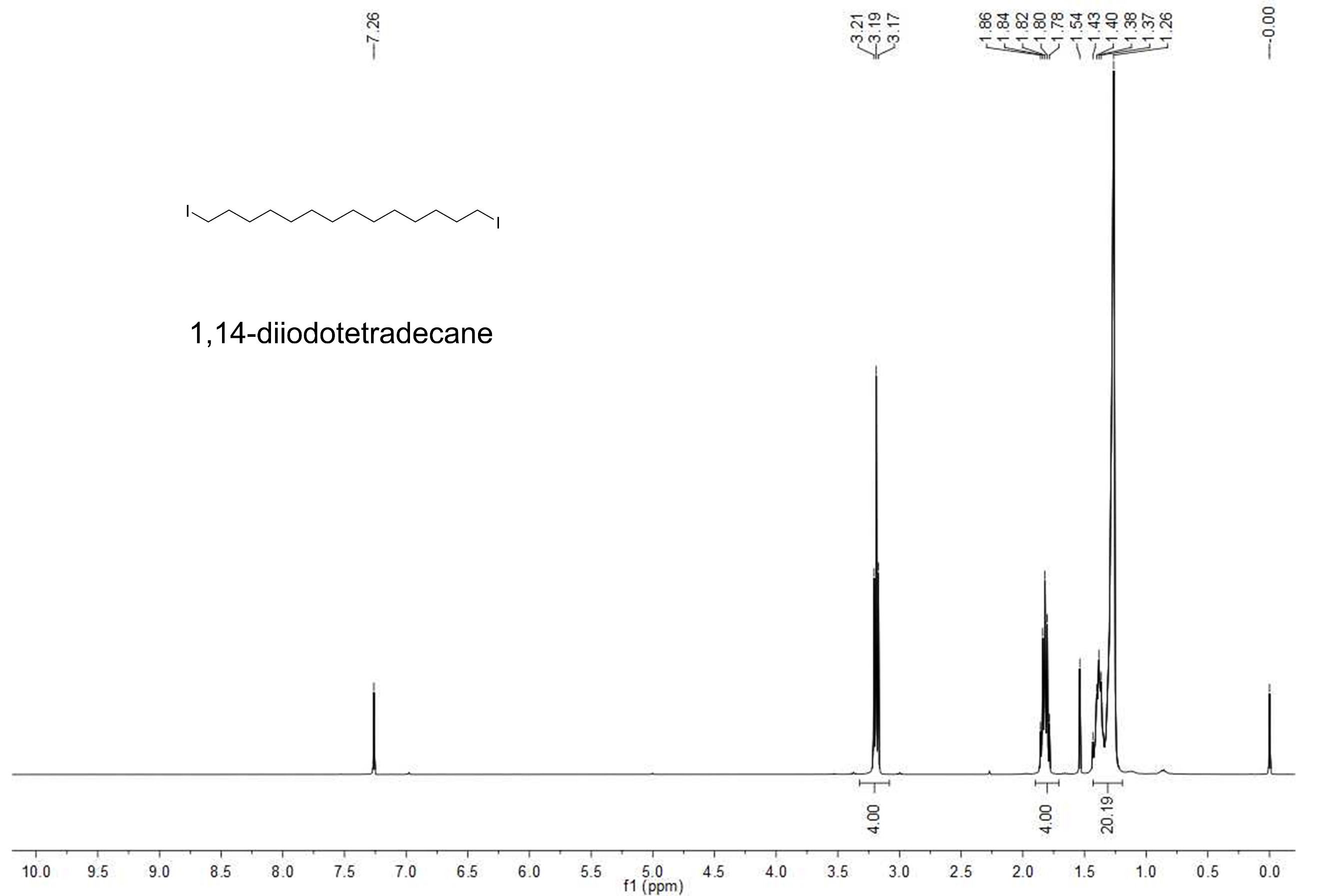


**Figure S70.** ^1^HNMR spectrum of 1,14-diiodotetradecane.


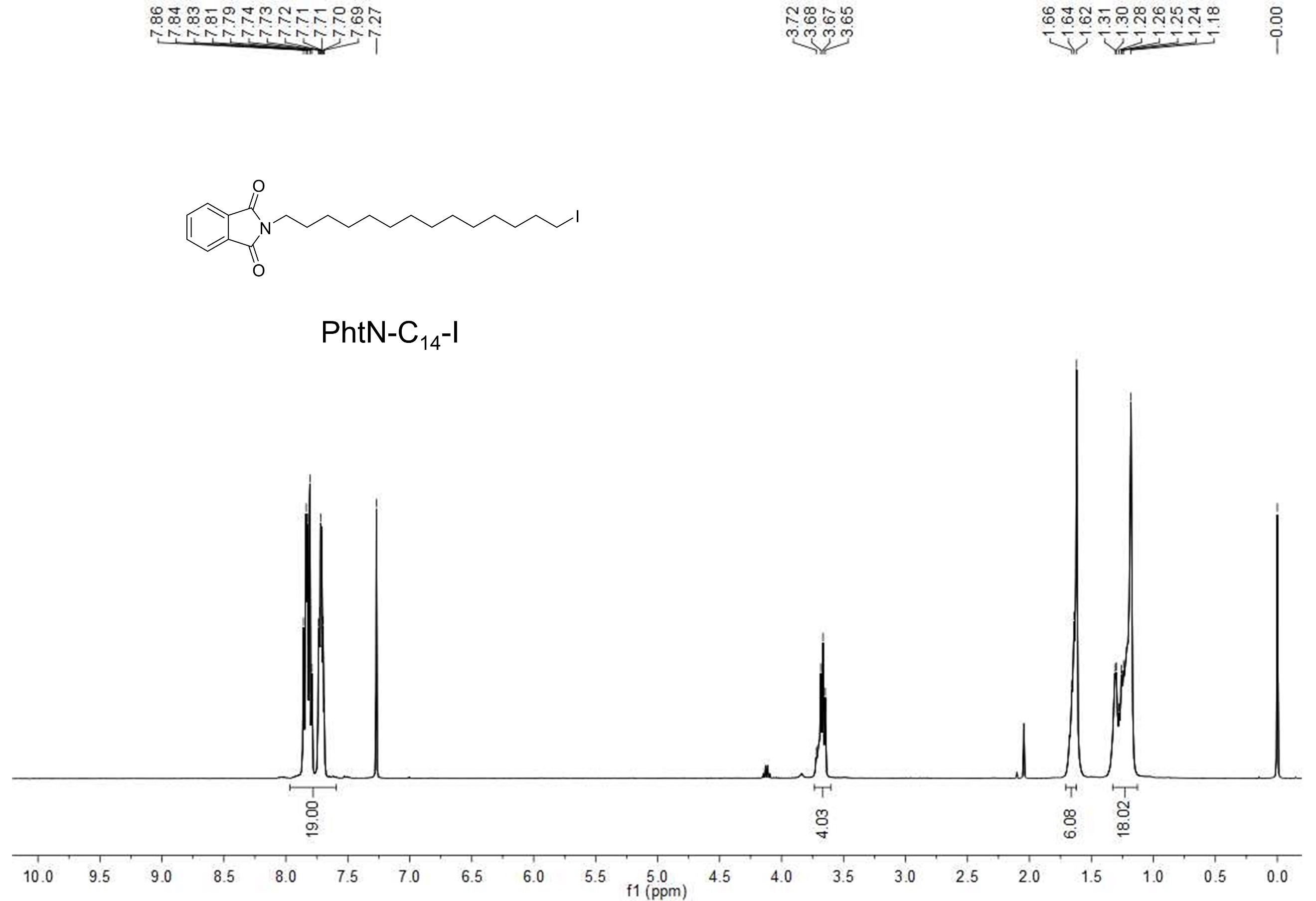


**Figure S71.** ^1^HNMR spectrum of PhtN-C_14_-I.


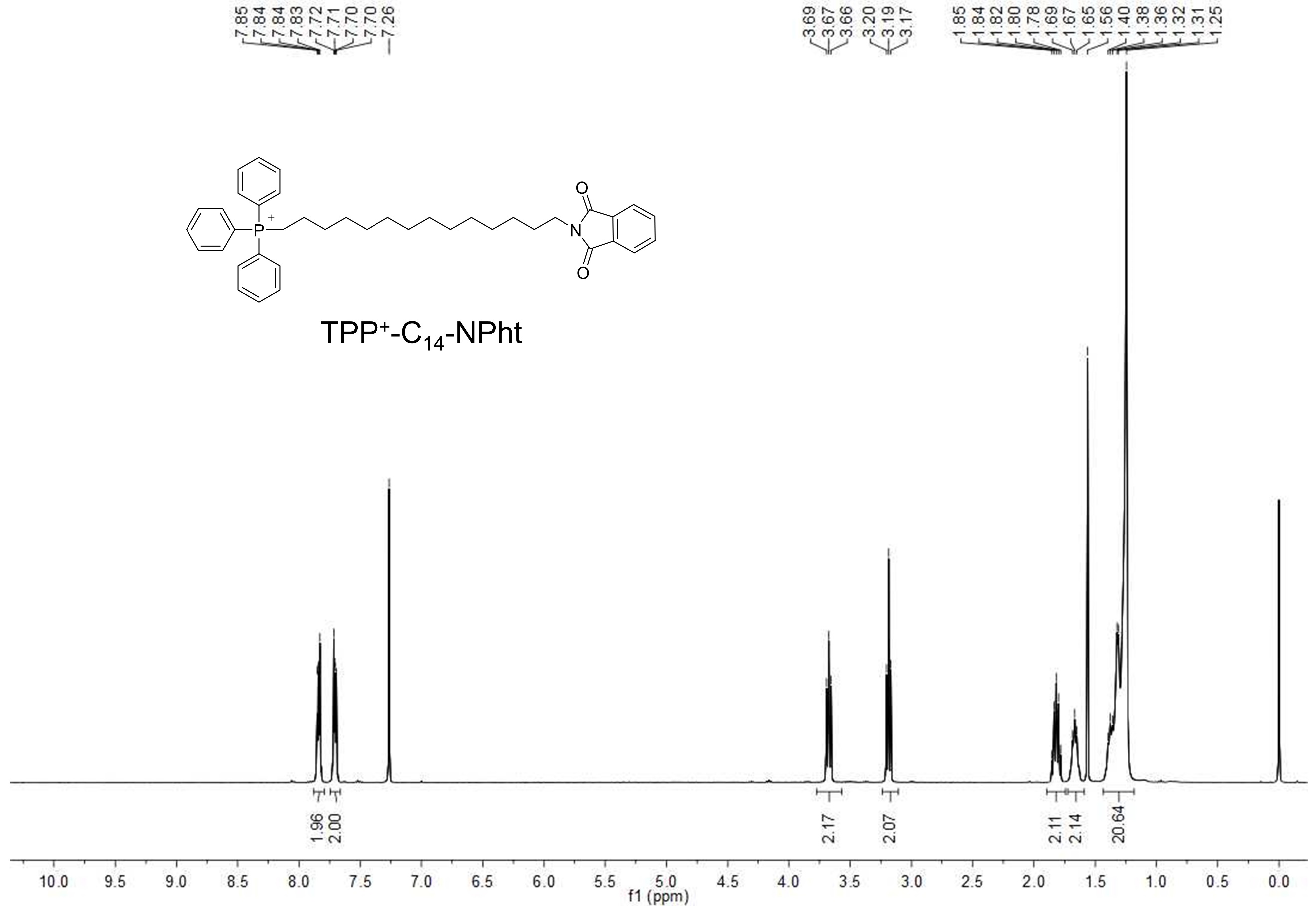


**Figure S72.** ^1^HNMR spectrum of TPP^+^-C_14_-NPht.


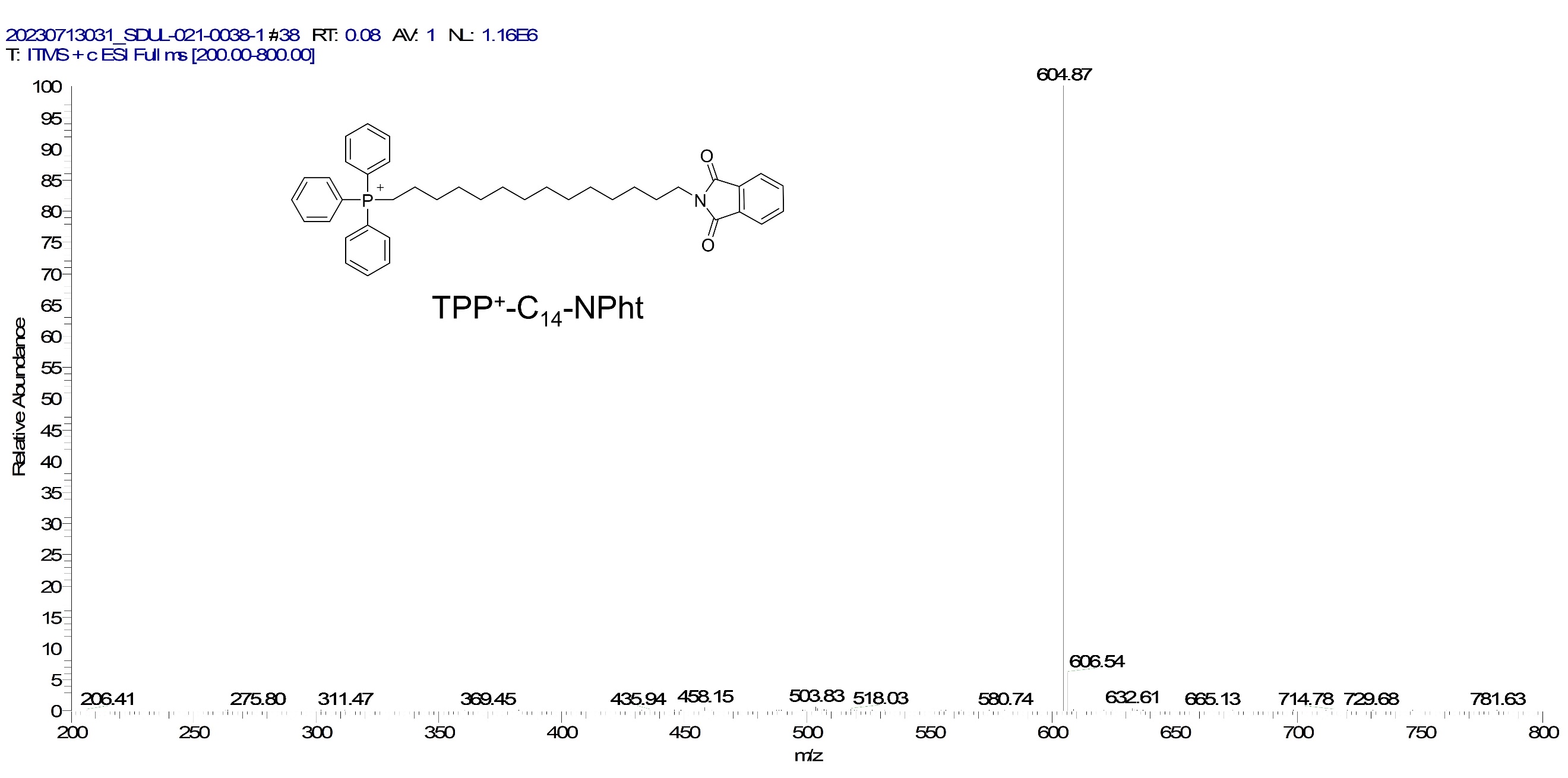


**Figure S73.** MS spectrum of TPP^+^-C_14_-NPht.


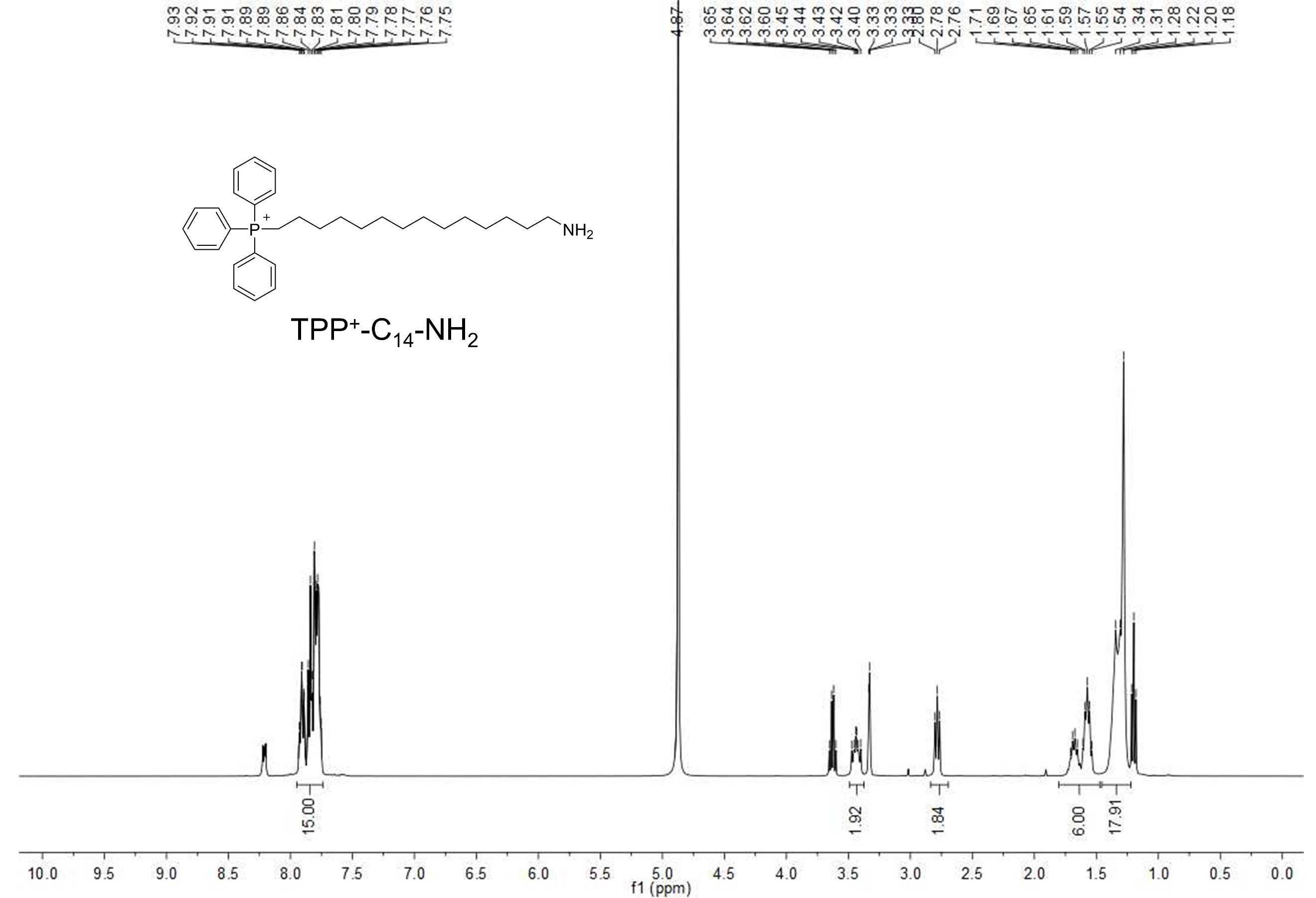


**Figure S74.** ^1^HNMR spectrum of TPP^+^-C_14_-NH_2_.


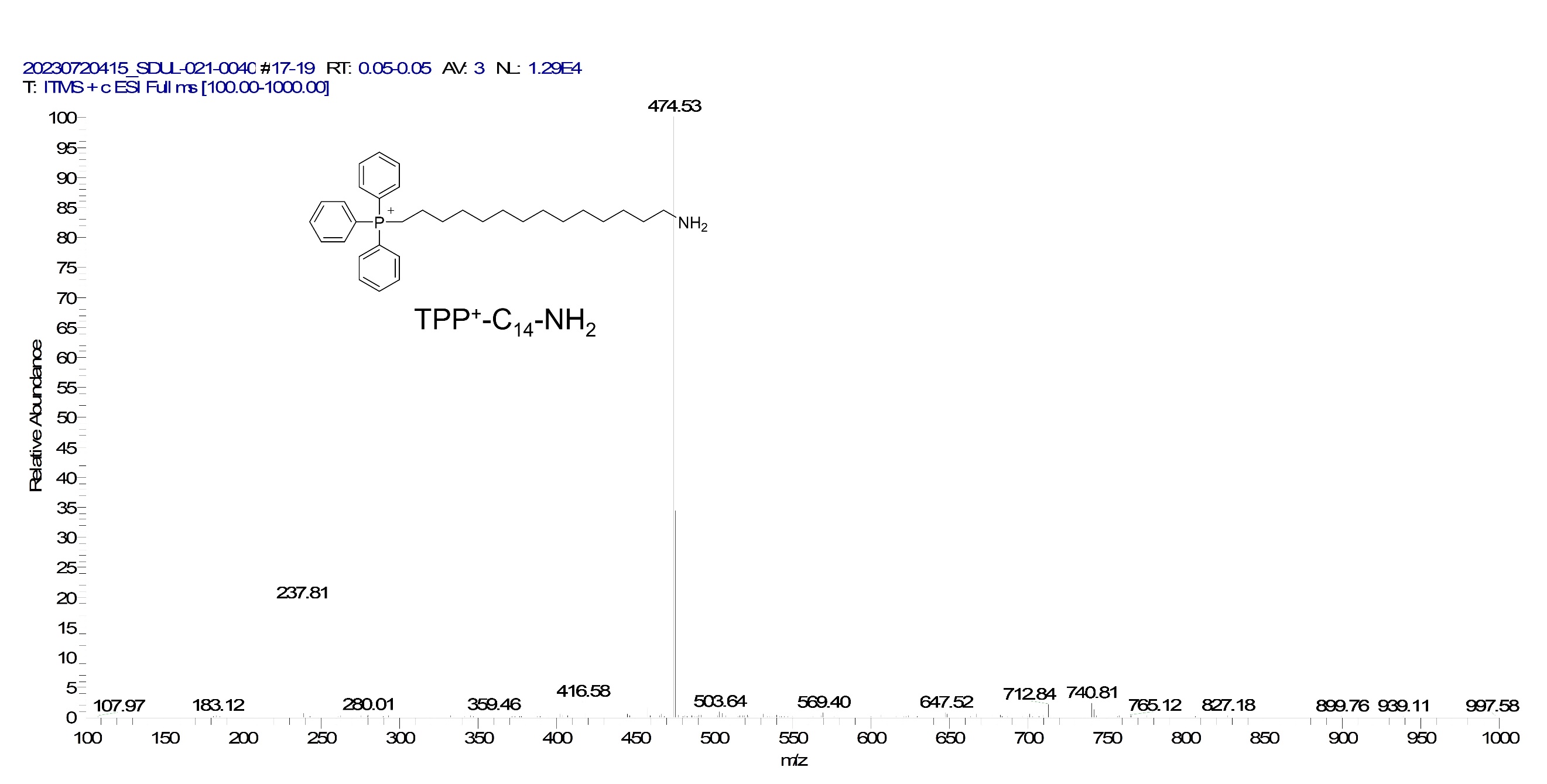


**Figure S75.** MS spectrum of TPP^+^-C_14_-NH_2._


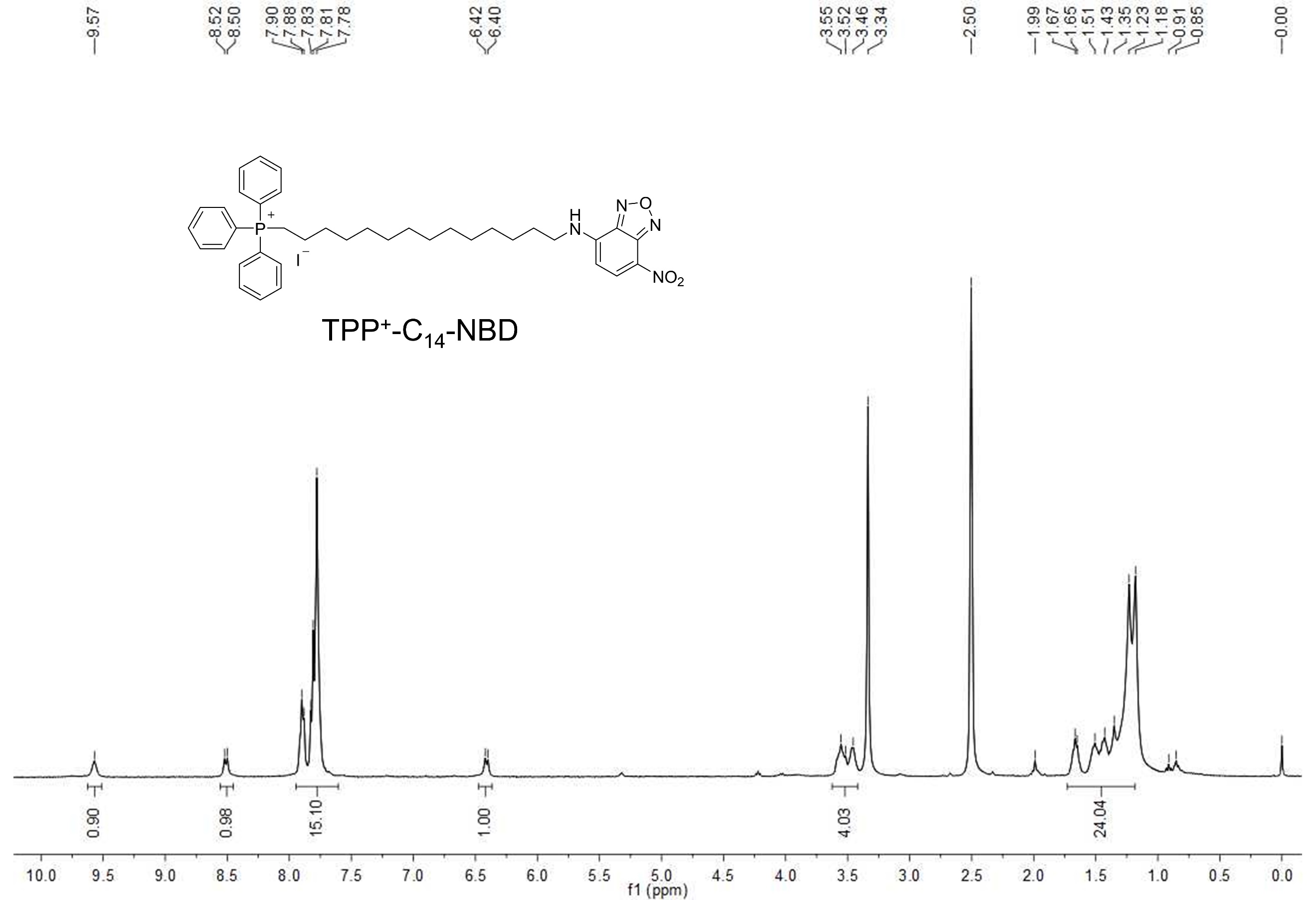


**Figure S76.** ^1^HNMR spectrum of TPP^+^-C_14_-NBD.


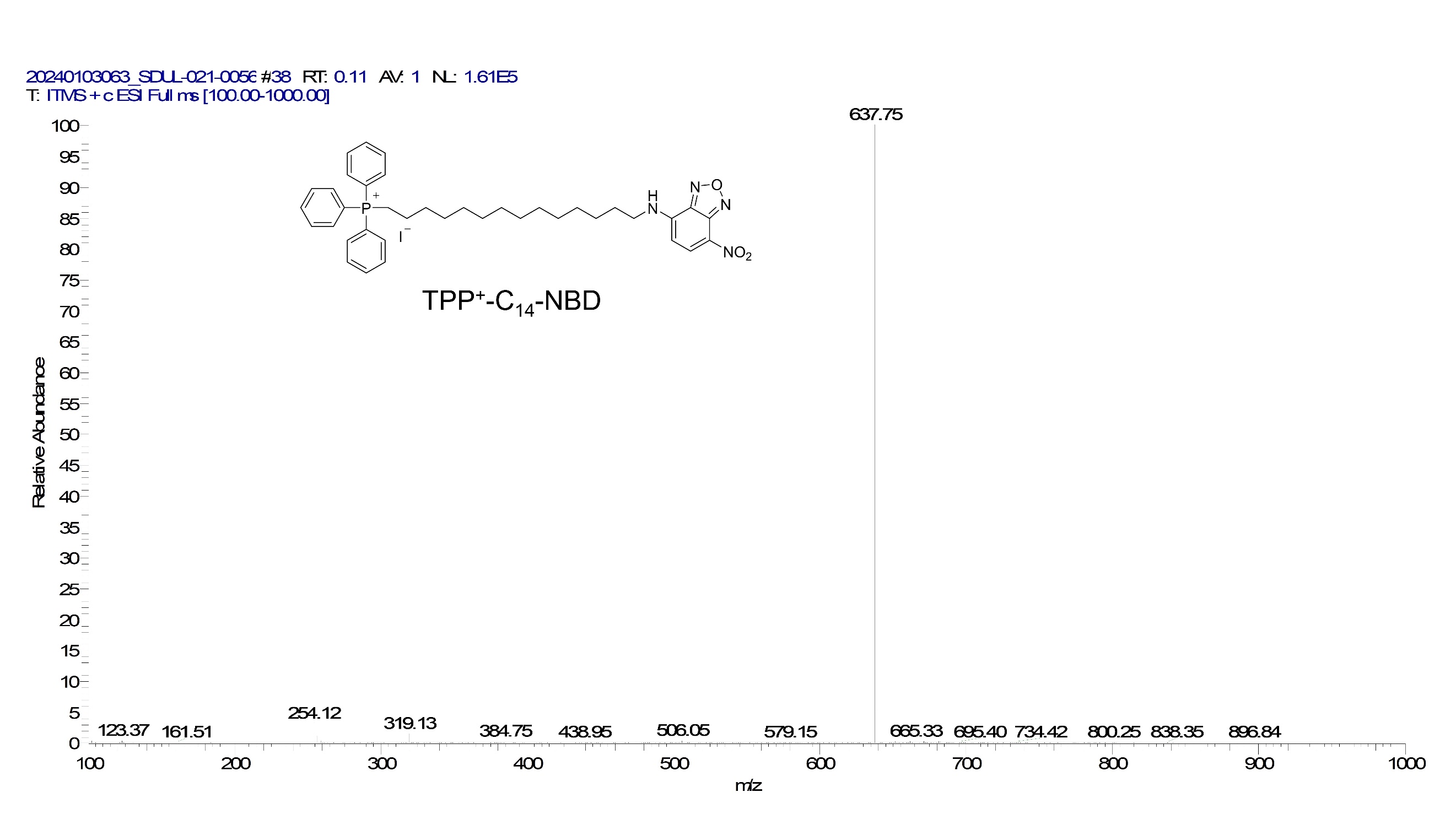


**Figure S77.** MS spectrum of TPP^+^-C_14_-NBD.


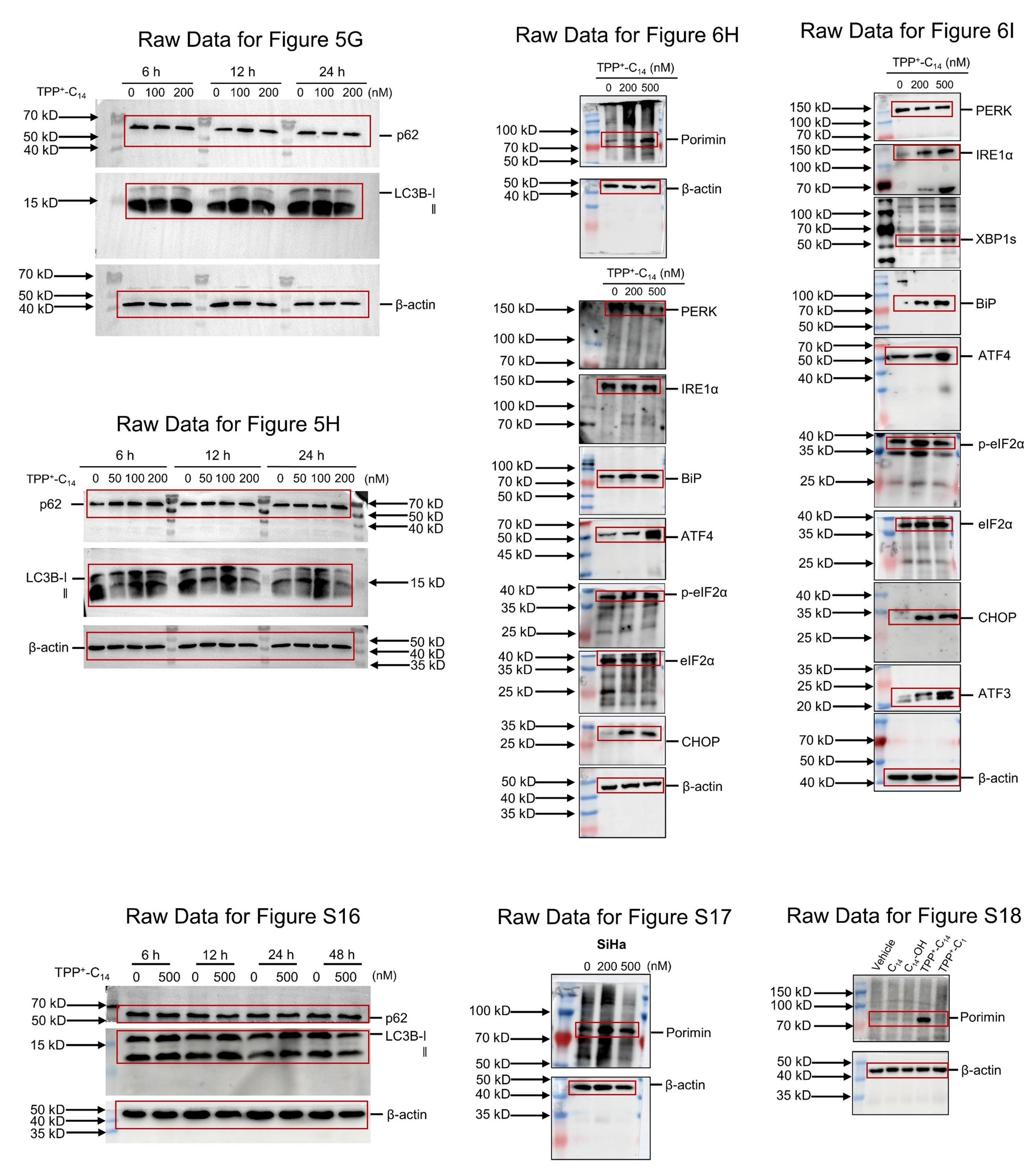


**Figure S78.** Raw western blot data for Figures 5G, 5H, 6H, 6I, S16, S17, and S18.

**Table S1.** IC_50_ values of each alkylTPP^+^ derivative against different cervical cancer cells upon 72-h treatment.

| Compound | IC_50_ (μM ) | | | |
| --- | --- | --- | --- | --- |
|  | HeLa | SiHa | Ca Ski | HeLa 229 |
| TPP^+^-C_1_ | 25.16±5.96 | 75.90±5.37 | 32.02±2.12 | 58.22±4.29 |
| TPP^+^-C_2_ | 20.04±1.19 | 55.52±2.17 | 19.22±1.09 | 23.21±0.91 |
| TPP^+^-C_3_ | 14.11±0.36 | 35.38±3.48 | 14.57±1.59 | 24.22±8.51 |
| TPP^+^-C_4_ | 7.75±1.25 | 29.60±1.81 | 8.89±0.53 | 18.80±0.69 |
| TPP^+^-C_5_ | 4.07±1.32 | 16.93±1.32 | 6.39±0.77 | 10.77±0.35 |
| TPP^+^-C_6_ | 1.93±0.26 | 6.52±1.13 | 3.08±0.48 | 4.17±0.16 |
| TPP^+^-C_7_ | 1.24±0.04 | 1.80±0.07 | 0.90±0.14 | 0.73±0.05 |
| TPP^+^-C_8_ | 0.64±0.02 | 1.17±0.16 | 0.79±0.06 | 0.74±0.08 |
| TPP^+^-C_9_ | 0.43±0.04 | 0.56±0.01 | 0.60±0.08 | 0.57±0.06 |
| TPP^+^-C_10_ | 0.22±0.01 | 0.54±0.02 | 0.41±0.02 | 0.32±0.02 |
| TPP^+^-C_11_ | 0.26±0.01 | 0.41±0.03 | 0.34±0.01 | 0.25±0.01 |
| TPP^+^-C_12_ | 0.18±0.003 | 0.40±0.10 | 0.38±0.05 | 0.34±0.03 |
| TPP^+^-C_13_ | 0.25±0.03 | 0.39±0.04 | 0.20±0.01 | 0.16±0.01 |
| TPP^+^-C_14_ | 0.20±0.003 | 0.30±0.02 | 0.18±0.01 | 0.18±0.02 |
| TPP^+^-C_16_ | 0.26±0.01 | 0.38±0.07 | 0.29±0.01 | 0.21±0.01 |
| TPP^+^-C_18_ | 0.46±0.10 | 0.58±0.03 | 0.40±0.03 | 0.33±0.03 |

**Table S2.** Binding parameters to cardiolipin of representative alkylTPP^+^ derivatives.

| Group | KD (μΜ) | K_on_ (1/Ms) | K_off_ (1/s) | Full R^2 |
| --- | --- | --- | --- | --- |
| NAO | 11.6±0.17 | 14350±165.8 | 0.168±0.0015 | 0.980 |
| TPP^+^-C_1_ | NA | NA | NA | NA |
| TPP^+^-C_4_ | NA | NA | NA | NA |
| TPP^+^-C_8_ | NA | NA | NA | NA |
| TPP^+^-C_9_ | 1.22±0.04 | 66900±1520 | 0.81±0.02 | 0.99 |
| TPP^+^-C_14_ | 6.58±0.09 | 7970±91 | 0.052±0.003 | 0.97 |
| TPP^+^-C_18_ | NA | NA | NA | NA |

**Table S3.** Primers used in qPCR studies.

| Primers for qPCR | |
| --- | --- |
| B2M F | AGATGAGTATGCCTGCCGTG |
| B2M R | GCGGCATCTTCAAACCTCCA |
| ATF4 F | CCCTTCACCTTCTTACAACCTC |
| ATF4 R | TGCCCAGCTCTAAACTAAAGGA |
| XBP1 F | CCCTCCAGAACATCTCCCCAT |
| XBP1 R | ACATGACTGGGTCCAAGTTGT |
| CHOP F | GGAAACAGAGTGGTCATTCCC |
| CHOP R | CTGCTTGAGCCGTTCATTCTC |
| BiP F | CATCACGCCGTCCTATGTCG |
| BiP R | CGTCAAAGACCGTGTTCTCG |
| CHAC1 F | CCTGAAGTACCTGAATGTGCGAGA |
| CHAC1 R | GCAGCAAGTATTCAAGGTTGTGGC |
| ADM2 F | CTGAGCCCCATCTGAAGCC |
| ADM2 R | CAGCACTGCGTGTAGACCAG |
| STC-2 F | GGGTGTGGCGTGTTTGAATG |
| STC-2 R | TTTCCAGCGTTGTGCAGAAAA |
